# Supplementary material for: A large-scale study across the avian clade identifies ecological drivers of neophobia
Source: PLoS Biol. 2025 Oct 14;23(10):e3003394. doi: 10.1371/journal.pbio.3003394 (PMC12520376; doi:10.1371/journal.pbio.3003394)
Supplement: S1 Text — Table A: Ecological factor coding. Table B: Inter-rater reliability. Table C: Repeatability (±95%CI) of birds’ responses at different grouping levels across control, novel object test and difference scores. Table D (a–c): Generalized linear mixed models using Markov chain Monte Carlo estimation methods (MCMCglmm models), testing the effect of avian order on the latency to touch familiar food when no novel object was present (i.e., raw data) on (a) control, (b) novel object test conditions or the (c) difference scores (novel object minus control values of latency to touch familiar food). Table E: Generalized linear mixed models using Markov chain Monte Carlo estimation methods (MCMCglmm models), testing the effect of predictors on birds’ difference scores in nondomesticated species only (seven domesticated species removed from data set; Model B). Table F: Generalized linear mixed models using Markov chain Monte Carlo techniques (MCMCglmm models), testing the effect of predictors on the difference (novel object minus control values) between control and novel object test latencies to touch familiar food (Model D). Table G: Site descriptions. Table H: Acknowledgements. Table I: Author contributions. Table J: Example test schedule and object (“obj”) counterbalancing across subjects. Fig A (a–c): The mean ± SE latency of birds to touch food in (a) control and (b) novel object test conditions, for each avian order. The data underlying this figure can be found in https://doi.org/10.6084/m9.figshare.27324972. Fig B: Mean+SE latency to touch food in control (orange) and novel object test (blue) conditions, in relation to habitat complexity (1 = high density; 3 = low density). The data underlying this figure can be found in https://doi.org/10.6084/m9.figshare.27324972. Fig C: Mean ± SE latency to touch food in control (orange) and novel object test (blue) conditions in relation to habitat use diversity, the number of habitat categories where a species was found (i.e., more hab [file pbio.3003394.s001.docx]

**S1 Text: Supporting Information**

**ManyBirds Project et al**

**A large-scale study across the avian clade identifies ecological drivers of neophobia**

**Table A: Ecological factor coding**

Full species list with ecological and other factors coded: Figshare 10.6084/m9.figshare.27324972

**Table B: Inter-rater reliability**

Inter-rater reliability: Figshare 10.6084/m9.figshare.27324972

**Table C: Repeatability (+95%CI) of birds’ responses at different grouping levels across control, novel object test and difference scores**. Repeatability was tested using the *rptR* function with individual ID fitted as a random effect for individual repeatability, or with individual ID as a nested random effect for species, family or order repeatability (e.g. for species - 1|animal/subject, with rpt group in the model, so extracted grname=”animal” returns repeatability of the species, see Stoffel et al., 2017 and <https://cran.r-project.org/web/packages/rptR/vignettes/rptR.html>).

| **Condition** | **Parameter** | **Repeatability** | **95%CI** |
| --- | --- | --- | --- |
| Control | Individual | 0.429 | 0.382/0.478 |
| Control | Species | 0.332 | 0.252/0.398 |
| Control | Family | 0.207 | 0.123/0.291 |
| Control | Order | 0.086 | 0.027/0.157 |
| Novel Object Test | Individual | 0.521 | 0.471/0.564 |
| Novel Object Test | Species | 0.499 | 0.417/0.565 |
| Novel Object Test | Family | 0.386 | 0.272/0.478 |
| Novel Object Test | Order | 0.252 | 0.132/0.361 |
| Difference score | Individual | 0.241 | 0.186/0.299 |
| Difference score | Species | 0.391 | 0.310/0.459 |
| Difference score | Family | 0.235 | 0.138/0.324 |
| Difference score | Order | 0.210 | 0.099/0.319 |

**Table D (*a, b, c*):** **Generalised linear mixed models using Markov chain Monte Carlo estimation methods (MCMCglmm models), testing the effect of avian order on the latency to touch familiar food when no novel object was present (i.e. raw data) on (a) control, (b) novel object test conditions or the (c) difference scores (novel object minus control values of latency to touch familiar food)**. *p*_MCMC_ is twice the posterior probability that the estimate is negative or positive (whichever probability is smallest), L. CI = lower 95% credible interval, U. CI = upper 95% credible interval. Significant predictors are indicated in bold. Eff. samp is the effective sample size of a Monte Carlo computation. Individual ID was the sole random effect in models.

| (a) **Latency to touch familiar food (control conditions)** | | | | | |
| --- | --- | --- | --- | --- | --- |
| Parameter | Post. mean | L. 95%CI | U. 95%CI | Eff. Samp. | pMCMC |
| (Intercept) | 519.15 | 411.15 | 635.44 | 1980 | **<0.001** |
| Anseriformes | -363.61 | -491.69 | -220.36 | 1980 | **<0.001** |
| Bucerotiformes | -245.77 | -407.81 | -69.60 | 1980 | **0.003** |
| Cariamiformes | -317.99 | -510.16 | -108.80 | 1980 | **0.002** |
| Casuariiformes | -419.20 | -642.87 | -193.58 | 1980 | **<0.001** |
| Cathartiformes | -326.96 | -586.25 | -34.46 | 1803 | **0.013** |
| Charadriiformes | -353.35 | -475.49 | -235.57 | 1980 | **<0.001** |
| Ciconiiformes | -306.83 | -508.33 | -88.95 | 1970 | **0.007** |
| Coliiformes | -326.76 | -652.09 | 4.66 | 1980 | 0.059 |
| Columbiformes | -330.22 | -569.84 | -117.62 | 1965 | **0.006** |
| Coraciiformes | -181.82 | -538.30 | 182.28 | 1980 | 0.344 |
| Cuculiformes | -316.08 | -650.37 | -37.42 | 1980 | **0.048** |
| Eurypygiformes | -463.11 | -1017.16 | 47.66 | 1915 | 0.084 |
| Falconiformes | -448.25 | -611.38 | -307.16 | 1980 | **<0.001** |
| Galliformes | -405.96 | -528.13 | -287.75 | 1980 | **<0.001** |
| Gruiformes | -400.26 | -608.84 | -195.13 | 1980 | **<0.001** |
| Musophagiformes | -73.71 | -280.02 | 137.84 | 1980 | 0.466 |
| Passeriformes | -321.40 | -431.57 | -201.90 | 1980 | **<0.001** |
| Pelecaniformes | -394.76 | -557.50 | -229.09 | 1825 | **<0.001** |
| Phoenicopteriformes | -49.95 | -189.52 | 87.03 | 1980 | 0.488 |
| Piciformes | -451.05 | -941.64 | 25.04 | 1980 | 0.071 |
| Podicipediformes | -339.52 | -956.59 | 285.77 | 1980 | 0.267 |
| Psittaciformes | -84.16 | -203.28 | 33.67 | 1980 | 0.186 |
| Rheiformes | -324.15 | -527.34 | -109.24 | 1980 | **0.004** |
| Sphenisciformes | -177.45 | -305.84 | -33.73 | 1980 | **0.012** |
| Strigiformes | -134.77 | -357.36 | 118.22 | 1846 | 0.283 |

| (b) **Latency to touch familiar food (novel object test conditions)** | | | | | |
| --- | --- | --- | --- | --- | --- |
| Parameter | Post. mean | L. 95%CI | U. 95%CI | Eff. Samp. | pMCMC |
| (Intercept) | 830.95 | 686.08 | 961.57 | 1980 | **<0.001** |
| Anseriformes | -120.91 | -295.55 | 40.30 | 1980 | 0.138 |
| Bucerotiformes | -474.37 | -680.31 | -257.25 | 1980 | **<0.001** |
| Cariamiformes | -222.73 | -473.74 | 40.33 | 1814 | 0.100 |
| Casuariiformes | -308.78 | -580.35 | -30.19 | 1980 | **0.035** |
| Cathartiformes | -246.93 | -573.06 | 76.11 | 1980 | 0.135 |
| Charadriiformes | -425.86 | -581.08 | -268.51 | 2178 | **<0.001** |
| Ciconiiformes | -197.77 | -428.10 | 82.21 | 1980 | 0.142 |
| Coliiformes | -504.83 | -912.05 | -114.86 | 1980 | **0.016** |
| Columbiformes | -337.81 | -631.82 | -78.19 | 1980 | **0.021** |
| Coraciiformes | 48.12 | -373.20 | 465.79 | 1980 | 0.843 |
| Cuculiformes | -112.74 | -471.58 | 276.65 | 1980 | 0.566 |
| Eurypygiformes | -613.23 | -1294.91 | 18.46 | 1980 | 0.073 |
| Falconiformes | -676.81 | -856.65 | -478.53 | 1980 | **<0.001** |
| Galliformes | -702.82 | -851.24 | -562.17 | 1980 | **<0.001** |
| Gruiformes | 131.96 | -124.05 | 382.63 | 1980 | 0.325 |
| Musophagiformes | 81.17 | -215.82 | 354.58 | 1980 | 0.565 |
| Passeriformes | -418.19 | -554.33 | -271.98 | 1980 | **<0.001** |
| Pelecaniformes | 55.46 | -155.37 | 262.02 | 1980 | 0.611 |
| Phoenicopteriformes | 239.62 | 83.31 | 424.20 | 1980 | **0.012** |
| Piciformes | -787.39 | -1353.95 | -183.32 | 2021 | **0.009** |
| Podicipediformes | 364.24 | -406.80 | 1136.62 | 1843 | 0.347 |
| Psittaciformes | -281.15 | -448.34 | -137.36 | 1980 | **<0.001** |
| Rheiformes | 17.16 | -231.98 | 291.10 | 1980 | 0.937 |
| Sphenisciformes | -58.00 | -220.12 | 114.22 | 1980 | 0.525 |
| Strigiformes | -224.72 | -538.85 | 74.65 | 2240 | 0.154 |

| (c) **Difference between control and novel object test conditions** | | | | | |
| --- | --- | --- | --- | --- | --- |
| Parameter | Post. mean | L. 95%CI | U. 95%CI | Eff. Samp. | pMCMC |
| (Intercept) | 351.63 | 238.02 | 480.09 | 1980 | **<0.001** |
| Anseriformes | 224.65 | 78.26 | 369.05 | 1980 | **0.004** |
| Bucerotiformes | -222.34 | -425.33 | -37.63 | 1805 | **0.023** |
| Cariamiformes | 58.15 | -157.01 | 313.61 | 1980 | 0.620 |
| Casuariiformes | 120.40 | -135.33 | 361.45 | 1980 | 0.368 |
| Cathartiformes | 37.80 | -224.89 | 337.20 | 1980 | 0.827 |
| Charadriiformes | -196.54 | -342.09 | -60.89 | 1980 | **0.003** |
| Ciconiiformes | -2.10 | -219.55 | 240.27 | 1980 | 0.975 |
| Coliiformes | -204.88 | -593.05 | 230.31 | 1846 | 0.325 |
| Columbiformes | -38.77 | -283.95 | 197.86 | 1927 | 0.748 |
| Coraciiformes | 197.04 | -186.57 | 608.02 | 2419 | 0.327 |
| Cuculiformes | 160.44 | -161.37 | 527.94 | 2134 | 0.352 |
| Eurypygiformes | -199.47 | -763.42 | 454.62 | 1980 | 0.541 |
| Falconiformes | -261.91 | -423.69 | -103.81 | 1980 | **0.002** |
| Galliformes | -318.15 | -448.78 | -184.62 | 1980 | **<0.001** |
| Gruiformes | 532.06 | 270.27 | 783.28 | 1980 | **<0.001** |
| Musophagiformes | 257.48 | -4.61 | 538.32 | 1980 | 0.064 |
| Passeriformes | -128.78 | -251.46 | -3.38 | 1980 | **0.036** |
| Pelecaniformes | 400.40 | 203.14 | 594.13 | 1980 | **<0.001** |
| Phoenicopteriformes | 265.79 | 115.29 | 437.55 | 2417 | **0.001** |
| Piciformes | -352.82 | -885.77 | 103.14 | 1980 | 0.152 |
| Podicipediformes | 688.66 | 123.90 | 1279.62 | 1980 | **0.017** |
| Psittaciformes | -202.20 | -338.61 | -68.37 | 1980 | **0.002** |
| Rheiformes | 309.93 | 85.36 | 548.88 | 1980 | **0.009** |
| Sphenisciformes | 43.36 | -102.91 | 185.83 | 1980 | 0.581 |
| Strigiformes | -131.39 | -387.41 | 148.41 | 1980 | 0.358 |

**Table E: Generalised linear mixed models using Markov chain Monte Carlo estimation methods (MCMCglmm models), testing the effect of predictors on birds’ difference scores in non-domesticated species only (seven domesticated species removed from data set; Model B).** Individual ID, nested within site, and phylogeny were included in the model as random effects to control for non-phylogenetic and phylogenetic non-independence, respectively, among individuals. *p*_MCMC_ is twice the posterior probability that the estimate is negative or positive (whichever probability is smallest), L. CI = lower 95% credible interval, U. CI = upper 95% credible interval. For migratory patterns, the 0 intercept in this 3-level factor is migratory species. Dietary breadth, habitat complexity and habitat use diversity were included in the model as continuous variables. Significant predictors are indicated in bold. Eff. samp is the effective sample size of a Monte Carlo computation.

|  | **post.mean** | **L. CI** | **U. CI** | **Eff. samp** | ***p*_MCMC_** |
| --- | --- | --- | --- | --- | --- |
| **Intercept** | 817.03 | 133.51 | 1580.90 | 1980 | **0.025** |
| **Social Context** | 87.73 | 15.28 | 164.46 | 1980 | **0.019** |
| Test Order (Novel Object Test Presented Second) | -43.55 | -88.31 | -0.19 | 1779 | 0.051 |
| Test Order (Both Same Day) | -12.25 | -165.11 | 126.02 | 1980 | 0.877 |
| Body Mass (log) | -38.43 | -97.40 | 18.96 | 1931 | 0.198 |
| **Dietary Breadth** | -58.00 | -104.83 | -9.27 | 1980 | **0.023** |
| Habitat use diversity | -12.91 | -53.40 | 28.65 | 1980 | 0.562 |
| Anthropogenic Habitat Use (Exploiter) | 64.55 | -51.24 | 183.62 | 1713 | 0.277 |
| habitat complexity | 92.52 | -7.62 | 212.07 | 1928 | 0.098 |
| Territoriality (Seasonal) | 8.89 | -192.56 | 205.18 | 1662 | 0.913 |
| Territoriality (Year-Round) | -86.99 | -303.37 | 147.86 | 2158 | 0.466 |
| Migration (Nomadic) | -227.55 | -592.14 | 176.71 | 1980 | 0.234 |
| **Migration (Non-migrant)** | -138.62 | -275.07 | -2.93 | 1980 | **0.047** |

**Table F: Generalised linear mixed models using Markov chain Monte Carlo techniques (MCMCglmm models), testing the effect of predictors on the latency to touch familiar food (i.e. raw data) in non-domesticated species only (seven domesticated species removed from data set; Model D).** Individual ID was nested within the study site, and species and phylogeny were included in the model as random effects to control for non-phylogenetic and phylogenetic non-independence, respectively, among individuals. *p*_MCMC_ is twice the posterior probability that the estimate is negative or positive (whichever probability is smallest), L. CI = lower 95 credible interval, U. CI = upper 95 credible interval. Significant predictors are indicated in bold.

|  | **post.mean** | **L. CI** | **U. CI** | **Eff. samp** | ***p*_MCMC_** |
| --- | --- | --- | --- | --- | --- |
| (Intercept) | 421.73 | -153.24 | 975.70 | 1932 | 0.141 |
| Condition (novel object test/control) | 97.79 | -85.54 | 282.97 | 1919 | 0.276 |
| Social Context | -1.80 | -56.26 | 60.37 | 1980 | 0.932 |
| Trial Order (second trial) | 16.14 | -17.79 | 57.24 | 1980 | 0.409 |
| Trial Order (both same day) | -54.99 | -157.04 | 51.28 | 1980 | 0.316 |
| Body Mass (log, grams) | -25.72 | -73.39 | 19.29 | 1980 | 0.293 |
| Dietary Breadth | 11.26 | -27.95 | 45.74 | 1980 | 0.563 |
| habitat complexity | 3.00 | -77.62 | 88.80 | 2176 | 0.919 |
| Anthropogenic Habitat Use | -12.66 | -104.75 | 76.73 | 1798 | 0.788 |
| Habitat use diversity | 26.10 | -8.07 | 58.55 | 1980 | 0.124 |
| Territoriality (Seasonal) | -94.50 | -231.53 | 71.22 | 1980 | 0.217 |
| Territoriality (Year-Round) | -85.03 | -258.53 | 86.57 | 1980 | 0.356 |
| Migration (Nomadic) | 191.25 | -99.07 | 482.65 | 1980 | 0.198 |
| Migration (Non-migrant) | -82.21 | -184.29 | 21.44 | 2122 | 0.134 |
| **Condition x Social Context** | 229.20 | 182.00 | 278.37 | 2361 | **<0.001** |
| Condition x Trial Order (second trial) | 45.37 | -12.65 | 105.55 | 1980 | 0.142 |
| Condition x Trial Order  (both same day) | 86.91 | -11.11 | 174.78 | 1980 | 0.076 |
| Condition x Body Mass | -0.09 | -11.26 | 12.80 | 1980 | 0.971 |
| **Condition x Dietary Breadth** | -20.08 | -37.29 | -2.72 | 1980 | **0.016** |
| **Condition x habitat complexity** | 53.89 | 9.71 | 89.11 | 2617 | **0.012** |
| Condition x Anthropogenic Habitat Use | -24.55 | -81.32 | 27.81 | 1980 | 0.378 |
| Condition x Habitat use diversity | -8.92 | -24.52 | 6.50 | 1980 | 0.256 |
| **Condition x Territoriality (Seasonal)** | 115.29 | 37.44 | 197.92 | 1980 | **0.004** |
| Condition x Territoriality (Year-Round) | 68.06 | -3.43 | 148.81 | 1802 | 0.080 |
| Condition x Migration (Nomadic) | -125.01 | -283.79 | 28.62 | 1980 | 0.131 |
| **Condition x Migration (Non-migrant)** | -79.82 | -140.88 | -21.31 | 1980 | **0.007** |

**Table G: Site descriptions**

Site descriptions including ethical approval per each site’s primary collaborator: Figshare 10.6084/m9.figshare.27324972

**Table H: Acknowledgements**

We thank the following organisations and people for facilitating this study by providing access to data collection sites or other contributions.

| **Name** | **Site** | **(Primary) Collaborator involved** | **Contribution** |
| --- | --- | --- | --- |
| Ian Edmans | Waddesdon Manor, Rothschild Foundation, National Trust, UK | Rachael Miller | Access to birds |
| Polly Bramham | Birdworld, Farnham, UK | Rachael Miller | Access to birds |
| Helen Hitchman | Cotswold Wildlife Park & Gardens, UK | Rachael Miller | Access to birds |
| Erica Caching | Zoo Marine, Italy | James Davies | Facilitating testing with birds |
| Stefan Stadler | Zoo Frankfurt, Germany | Kai R. Caspar | Access to birds |
| Kerstin Ternes | Zoo Duisburg, Germany | Kai R. Caspar | Access to birds |
| Alexander Nolte | Zoo Duisburg, Germany | Kai R. Caspar | Access to birds |
| Antje Bauersfeld | Zoo Dortmund, Germany | Kai R. Caspar | Access to birds |
| Stephanie Zech | Zoo Dortmund, Germany | Kai R. Caspar | Access to birds |
| Sandra Dollhäupl | Zoo Duisburg | Kai R. Caspar | Access to birds |
| Bernd Marcordes | Zoo Köln, Germany | Kai R. Caspar | Access to birds |
| Alexander Sliwa | Zoo Köln, Germany | Kai R. Caspar | Access to birds |
| Arne  Lawrenz | Zoo Wuppertal, Germany | Dominik Fischer | Access to birds |
| Karl  Fischer | Raptor Center & Wildlife Zoo Hellenthal | Dominik Fischer | Access to birds |
| Wolfgang Kiessling | Loro Parque & Loro Parque Fundacion, Spain | Auguste von Bayern & Anastasia Krasheninnikova | Access to birds |
| Christoph Kiessling | Loro Parque & Loro Parque Fundacion, Spain | Auguste von Bayern & Anastasia Krasheninnikova | Access to birds |
| Jessica Borer | Zoo Basel, Switzerland | Vanessa Wilson | Access to birds |
| David Auz, Courtney Pike, Sabine Tebbich, Irm Teschke,  CDRS staff | Charles Darwin Research Station | Paula Ibáñez de Aldecoa | Access to birds, facilitating testing, resources |
| Marije van de Poll |  | Jorg Massen | Code videos for IRR test |
| Natalia Demergassi | Fundación Temaikèn | Jimena Lois-Milevicich | Access to birds & birds’ info, facilitating  testing |
| Andrés Suárez | Fundación Temaikèn | Jimena Lois-Milevicich | Access to birds & birds’ info, facilitating  testing |
| Zookeepers from CRET, ELA & “Patagonia” (Sector 4) | Fundación Temaikèn | Jimena Lois-Milevicich | Access to birds & birds’ info, facilitating  testing |
| Bettina Mahler | IEGEBA, CONICET | Jimena Lois-Milevicich | Resources (filming camera) |
| Štěpán Drda | University of South Bohemia | Petr Veselý | Facilitating testing |
| Margeaux Apple | Cambridge University Botanic Gardens | Julia Mackenzie | Facilitating site access |
| Grim Stjerndahl | Ystad Djurpark | Stephan A. Reber | Access to birds |
| The Pole Evans Family | Saunders Island, Falkland Islands | Katie Harrington | Facilitating site access, Resources |
| Nora Tabea Kopsch |  | Katie Harrington | Coded videos for IRR test |
| Daniel Issel |  | Kai R. Caspar | Coded videos for IRR test |
| Paul Emile Victor Institute | Kerguelen Islands (Verte Island) | Samara Danel | Facilitating site access and testing |
| Andrea Bračko | Zagreb Zoo | Elena Racevska | Access to birds |
| Innes Bliznac | Zagreb Zoo | Elena Racevska | Facilitating testing |
| Valentino Macan | Zagreb Zoo | Elena Racevska | Facilitating testing |
| Herbert Linnemann | Heimat-Tierpark Olderdissen, Bielefeld, Germany | Kai-Philipp Gladow | Access to birds |
| Markus Hinker | Heimat-Tierpark Olderdissen, Bielefeld, Germany | Kai-Philipp Gladow | Access to birds |
| Benjamin Aschmann | Adlerwarte Berlebeck, Detmold, Germany | Kai-Philipp Gladow & Kai R. Caspar | Access to birds |
| Stefan Schierhuber |  | Theresa Rössler & Alice Auersperg | Coded videos for IRR |
| Mariah Monsour | Pittsburg State University Natural History Reserve, Pittsburg, USA | Laurent Prétôt | Coded videos for IRR, Assisted with testing |

**Table I: Author contributions**

Full list of authors, affiliations, funding and author contributions: Figshare 10.6084/m9.figshare.27324972 (private link:<https://figshare.com/s/046099ac8313ae194abf>)

**Table J:** **Example test schedule and object (“obj”) counterbalancing across subjects.** *Or day 3 (within 48 hours of first presentation) and was consistent across all subjects for the duration of the study. Note that all subjects were tested with the same object in Round 1.

| **Week** | **Day** | **Round** | **Bird 1** | **Bird 2** | **Bird 3** | **Bird 4** | **Bird 5** | **Bird 6** |
| --- | --- | --- | --- | --- | --- | --- | --- | --- |
| 1 | 1 | 1 | Obj 1 | Obj 1 | Obj 1 | Control | Control | Control |
|  | 2* |  | Control | Control | Control | Obj 1 | Obj 1 | Obj 1 |
| 4 | 1 | 2 | Obj 2 | Obj 3 | Obj 2 | Control | Control | Control |
|  | 2* |  | Control | Control | Control | Obj 3 | Obj 2 | Obj 3 |
| 6 | 1 | 3 | Obj 3 | Obj 2 | Obj 3 | Control | Control | Control |
|  | 2* |  | Control | Control | Control | Obj 2 | Obj 3 | Obj 2 |

**
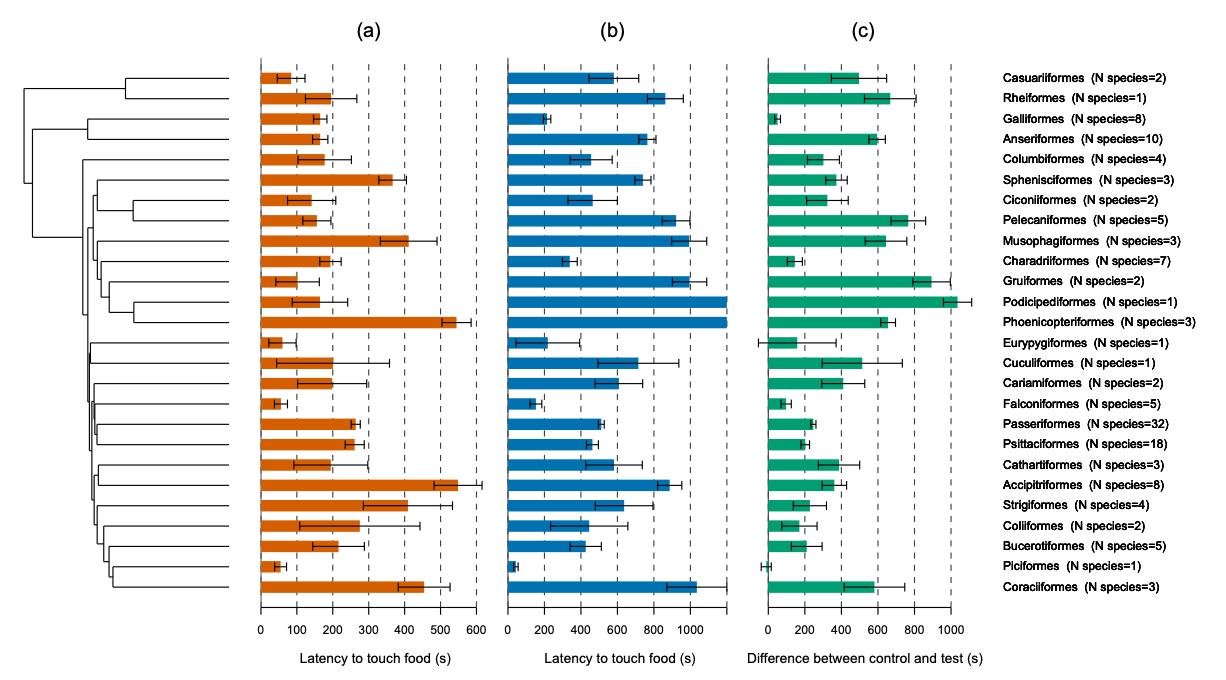
**

**Fig A. *a, b and c*: The mean+SE latency of birds to touch food in (a) control and (b) novel object test conditions, for each avian order.** The mean+SE difference (individual difference) between control and novel object test conditions (c) for each order are shown. The data underlying this figure can be found in DOI: 10.6084/m9.figshare.27324972


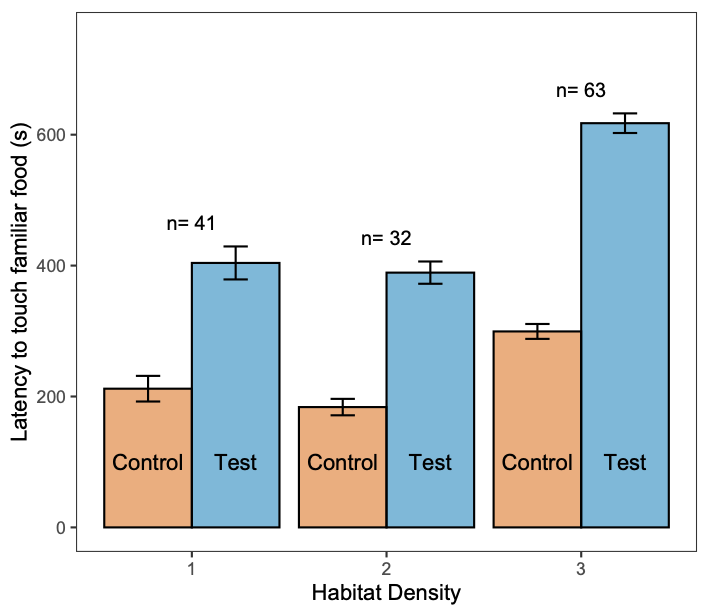


**Fig B. Mean+SE latency to touch food in control (orange) and novel object test (blue) conditions, in relation to habitat complexity (1 = high complexity; 3 = low complexity; raw data; all species).** Sample sizes are given above the bars. The data underlying this figure can be found in DOI: 10.6084/m9.figshare.27324972


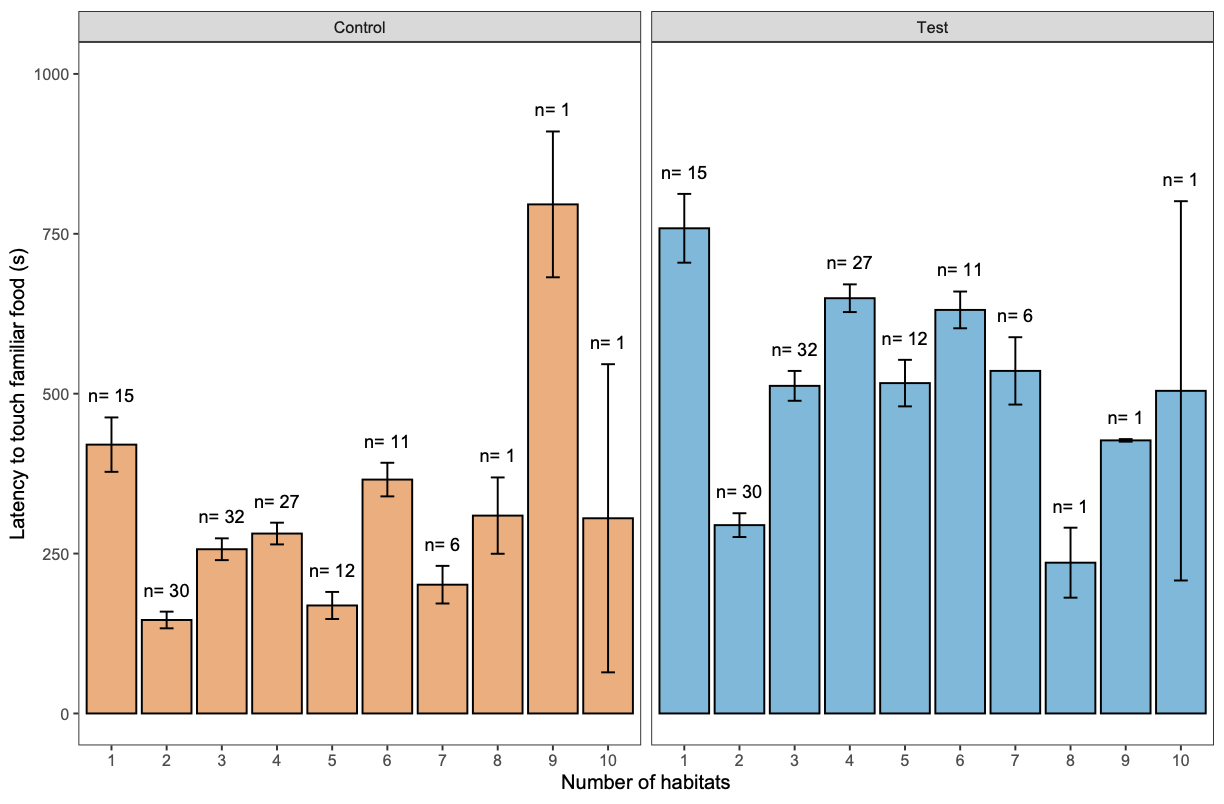


**Fig C. Mean+SE latency to touch food in control (orange) and novel object test (blue) conditions in relation to habitat use diversity, the number of habitat categories where a species was found (i.e. more habitat generalists vs specialists; raw data; all species).** Sample sizes are given above the bars. The data underlying this figure can be found in DOI: 10.6084/m9.figshare.27324972

**
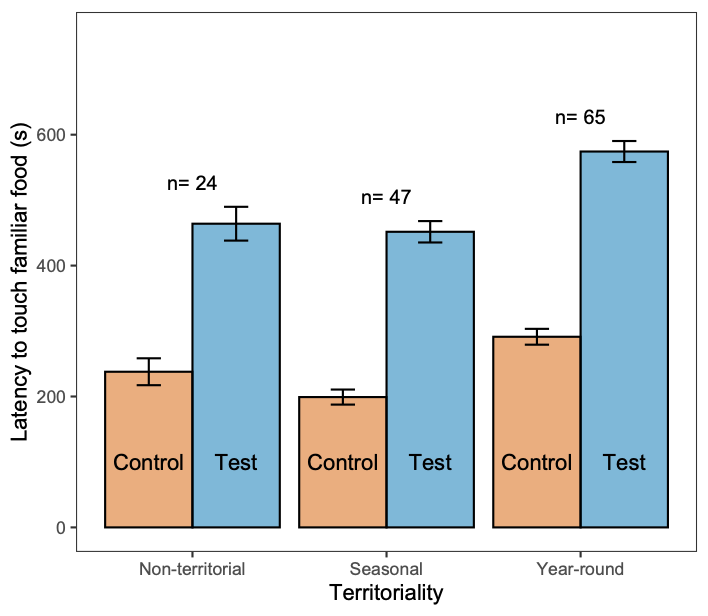
**

**Fig D. Mean+SE latency to touch food in control (orange) and novel object test (blue) conditions in territorial systems (non-territorial, seasonal or year-round territories) in non-domesticated species only (raw data).** Sample sizes are given above the bars. The data underlying this figure can be found in DOI: 10.6084/m9.figshare.27324972

**Information A: Avian order comparisons**

The phylogenetic signal (overall novel object test and control conditions models; Pagel’s lambda) calculated from the Bayesian GLMM model was 0.64 (0.55/0.71) and for difference scores it was 0.68 (0.60/0.76), indicating an intermediate phylogenetic signal. Avian order affected the latency to touch familiar food in the control (Table D*a* in S1 Text; Fig A in S1 Text. a) and the novel object test conditions (Table D*b* in S1 Text; Fig A in S1 Text. b). In the novel object test condition, Phoenicopteriformes (flamingos) and Podicipediformes (grebes, represented only by the little grebe, *Tachybaptus ruficollis*) had the longest latencies to touch food (and many did not touch food at all when the novel item was present), whereas Piciformes (represented only by the black-necked aracari, *Pteroglossus aracari*), Falconiformes (falcons) and Galliformes (pheasants and allies) were quickest (Fig A*b* in S1 Text). In the control condition, the longest latencies to touch food were found in Accipitriformes (hawks and allies) and Phoenicopteriformes (flamingos), whereas Falconiformes (falcons), Piciformes and Eurypygiformes (represented by the sunbittern, *Eurypyga helias*) were quickest to touch food (Fig A*a* in S1 Text). Overall, we found a difference between control and novel object test latencies across avian orders (Table D*c* in S1 Text; Fig A in S1 Text. c), with the biggest overall change between conditions observed (i.e. indicating higher neophobia) in Podicipediformes and Gruiformes (cranes and allies) and the smallest change (i.e. indicating lower neophobia) in Falconiformes, Piciformes and Galliformes (Fig A*c* in S1 Text). **​**
