# Supplementary material for: A large-scale study across the avian clade identifies ecological drivers of neophobia
Source: PLoS Biol. 2025 Oct 14;23(10):e3003394. doi: 10.1371/journal.pbio.3003394 (PMC12520376; doi:10.1371/journal.pbio.3003394)
Supplement: Attachment — Submitted filename: PBIOLOGY-D-24-03430_R1_annotated.pdf [file pbio.3003394.s003.pdf]

// PLOS Biology

Evolutionary drivers of neophobia across the avian clade

--Manuscript Draft--

**Manuscript Number:**	PBIOLOGY-D-24-03430R1
**Full Title:**	Evolutionary drivers of neophobia across the avian clade
**Short Title:**	Neophobia in birds
**Article Type:**	Initial Research Submission
**Keywords:**	object neophobia; response to novelty; birds; socio-ecological drivers; big team science; open science; behavioural flexibility
**Abstract:**	Neophobia, or aversion to novelty, is important for adaptability and survival as it influences the ways in which animals navigate risk and interact with their environments. Across taxa, species and individuals, neophobia is found to vary considerably, but exploring the wider evolutionary drivers of neophobia is hampered by a lack of comparative multi-species studies using standardised methods. Here, we utilised the ManyBirds Project, a Big Team Science large-scale collaborative open science framework, to pool efforts and resources of 129 collaborators at 77 institutions from 23 countries worldwide across six continents. We examined both difference scores (between novel object test and control conditions) and raw data of latency to touch familiar food in the presence (test) and absence (control) of a novel object among 1439 subjects from 136 bird species across 25 taxonomic orders incorporating lab, field and zoo sites. We tested effects of 1) taxonomic order differences; 2) socio-ecological factors including diet, sociality, habitat and range, while accounting for phylogeny; and 3) individual repeatability in object neophobia. We found that at the order level, neophobia was markedly affected by phylogeny. At the species level, using difference scores, species with more specialist diets were more neophobic than those with more generalist diets, providing strong support for the Neophobia Threshold Hypothesis. Migratory species were also more neophobic than non-migratory species, which supports the Dangerous Niche Hypothesis. Finally, we found individual repeatability across time (i.e. across test rounds) and contexts (i.e. controls and novel object tests). Using the largest-scale multi-order study of neophobia to date, our approach and results show that neophobia is a stable behavioural trait predicted by phylogeny. We have identified evolutionary drivers of neophobia across the avian clade and demonstrated the potential of Big Team Science research to reveal broad patterns of bird behaviour.
**Additional Information:**	
**Question**	**Response**
**Financial Disclosure**<br><br>Enter a financial disclosure statement that describes the sources of funding for the work included in this submission. Review the submission guidelines for detailed requirements. View published research articles from *PLOS Biology* for specific examples.<br><br>This statement is required for submission and **will appear in the published article** if the submission is accepted. Please make sure it is accurate.	Yes

Powered by Editorial Manager® and ProduXion Manager® from Aries Systems Corporation

**Funded studies**<br>Enter a statement with the following details:<br>• Initials of the authors who received each award<br>• Grant numbers awarded to each author<br>• The full name of each funder<br>• URL of each funder website<br>• Did the sponsors or funders play any role in the study design, data collection and analysis, decision to publish, or preparation of the manuscript?<br><br>Did you receive funding for this work?	
Please add funding details.<br>as follow-up to "**Financial Disclosure**<br><br>Enter a financial disclosure statement that describes the sources of funding for the work included in this submission. Review the submission guidelines for detailed requirements. View published research articles from *PLOS Biology* for specific examples.<br><br>This statement is required for submission and **will appear in the published article** if the submission is accepted. Please make sure it is accurate.<br><br>**Funded studies**<br>Enter a statement with the following details:<br>• Initials of the authors who received each award<br>• Grant numbers awarded to each author<br>• The full name of each funder<br>• URL of each funder website<br>• Did the sponsors or funders play any role in the study design, data collection and analysis, decision to publish, or preparation of the manuscript?<br><br>Did you receive funding for this work?"	Research Support Development Grant, Anglia Ruskin University to Rachael Miller. Other funding that directly supported contribution to this study through individually acquired awards is outlined in S6 Table.
Please select the country of your main research funder (please select carefully as in some cases this is used in fee calculation).	UNITED KINGDOM - GB

as follow-up to "**Financial Disclosure**<br><br>Enter a financial disclosure statement that describes the sources of funding for the work included in this submission. Review the submission guidelines for detailed requirements. View published research articles from *PLOS Biology* for specific examples.<br><br>This statement is required for submission and **will appear in the published article** if the submission is accepted. Please make sure it is accurate.<br><br>**Funded studies**<br>Enter a statement with the following details:<br>• Initials of the authors who received each award<br>• Grant numbers awarded to each author<br>• The full name of each funder<br>• URL of each funder website<br>• Did the sponsors or funders play any role in the study design, data collection and analysis, decision to publish, or preparation of the manuscript?<br><br>Did you receive funding for this work?"	
**Competing Interests**<br><br>You are responsible for recognizing and disclosing on behalf of all authors any competing interest that could be perceived to bias their work, acknowledging all financial support and any other relevant financial or non-financial competing interests.<br><br>Do any authors of this manuscript have competing interests (as described in the PLOS Policy on Declaration and Evaluation of Competing Interests)?	The authors have declared that no competing interests exist.

Powered by Editorial Manager® and ProduXion Manager® from Aries Systems Corporation

**If yes**, please provide details about any and all competing interests in the box below. Your response should begin with this statement: *I have read the journal's policy and the authors of this manuscript have the following competing interests:*<br><br>**If no** authors have any competing interests to declare, please enter this statement in the box: "*The authors have declared that no competing interests exist.*"<br><br>* typeset<br><br>This statement is required for submission and **will appear in the published article** if the submission is accepted. Please make sure it is accurate and that any funding sources listed in your Funding Information later in the submission form are also declared in your Financial Disclosure statement.	
**Ethics Statement**<br><br>You must include an Ethics Statement in the Methods section of your manuscript if your study involved:<br>• Human participants<br>• Human specimens or tissue<br>• Vertebrate animals or cephalopods<br>• Vertebrate embryos or tissues<br>• Field research<br><br>**Not including a statement when required will delay the review process.**<br><br>General guidance is provided below. Please consult the guidelines on human subjects research and animal research for detailed instructions.	I confirm that I have read and understood PLOS' requirements for an Ethics Statement.

**Format for specific study types**<br><br>**Human Subject Research (involving human participants and/or tissue)**<br>• Give the name of the institutional review board or ethics committee that approved the study<br>• Include the approval number and/or a statement indicating approval of this research<br>• Indicate the form of consent obtained (written/oral) or the reason that consent was not obtained (e.g. the data were analyzed anonymously)<br><br>**Animal Research (involving vertebrate animals, embryos or tissues)**<br>• Provide the name of the Institutional Animal Care and Use Committee (IACUC) or other relevant ethics board that reviewed the study protocol, and indicate whether they approved this research or granted a formal waiver of ethical approval<br>• Include an approval number if one was obtained<br>• If the study involved *non-human primates*, add *additional details* about animal welfare and steps taken to ameliorate suffering<br><br>**Field Research**<br><br>Include the following details if this study involves the collection of plant, animal, or other materials from a natural setting:<br>• Field permit number<br>• Name of the institution or relevant body that granted permission<br><br>Please check the box to confirm your understanding of this policy, then read and agree to one of the statements.	
Please respond:<br>as follow-up to "**Ethics Statement**<br><br>You must include an Ethics Statement in	My study requires an ethics statement. I confirm I have included my statement in the Methods section of my uploaded manuscript file.

the Methods section of your manuscript if your study involved:<br>• Human participants<br>• Human specimens or tissue<br>• Vertebrate animals or cephalopods<br>• Vertebrate embryos or tissues<br>• Field research<br><br>**Not including a statement when required will delay the review process.**<br><br>General guidance is provided below. Please consult the guidelines on human subjects research and animal research for detailed instructions.	

**Format for specific study types**<br><br>**Human Subject Research (involving human participants and/or tissue)**<br>• Give the name of the institutional review board or ethics committee that approved the study<br>• Include the approval number and/or a statement indicating approval of this research<br>• Indicate the form of consent obtained (written/oral) or the reason that consent was not obtained (e.g. the data were analyzed anonymously)<br><br>**Animal Research (involving vertebrate animals, embryos or tissues)**<br>• Provide the name of the Institutional Animal Care and Use Committee (IACUC) or other relevant ethics board that reviewed the study protocol, and indicate whether they approved this research or granted a formal waiver of ethical approval<br>• Include an approval number if one was obtained<br>• If the study involved *non-human primates*, add *additional details* about animal welfare and steps taken to ameliorate suffering<br><br>**Field Research**<br><br>Include the following details if this study involves the collection of plant, animal, or other materials from a natural setting:<br>• Field permit number<br>• Name of the institution or relevant body that granted permission<br><br>Please check the box to confirm your understanding of this policy, then read and agree to one of the statements. "	
**Data Availability**<br><br>PLOS journals require authors to make all data underlying the findings described in their manuscript fully available, without restriction and from the time of	Yes - all data are fully available without restriction

publication, with only rare exceptions to address legal and ethical concerns (see the PLOS Data Policy and FAQ for further details). When submitting a manuscript, authors must provide a Data Availability Statement that describes where the data underlying their manuscript can be found.<br><br>Your answers to the following constitute your statement about data availability and will be included with the article in the event of publication. **Please note that simply stating 'data available on request from the author' is not acceptable.** ***If, however, your data are only available upon request from the author(s), you must answer "No" to the first question below, and explain your exceptional situation in the text box provided.***<br><br>Do the authors confirm that all data underlying the findings described in their manuscript are fully available without restriction?	
Please describe where your data may be found, writing in full sentences. **Your answers should be entered into the box below and will be published in the form you provide them, if your manuscript is accepted.** If you are copying our sample text below, please ensure you replace any instances of XXX with the appropriate details.<br><br>• If your data are all contained within the paper and/or Supporting Information files, please state this in your answer below. For example, "All relevant data are within the paper and its Supporting Information files."<br>• If your data are held or will be held in a public repository, include URLs, accession numbers or DOIs. For example, "All XXX files are available from the XXX database (accession	The full data set (including species with 600 second trial cut off), final analysis data set (removing species with 600 second trial cut off) and R coding script are available on: Figshare 10.6084/m9.figshare.27324972 (private link: https://figshare.com/s/046099ac8313ae194abf)

number(s) XXX, XXX)." If this information will only be available after acceptance, please indicate this by ticking the box below.<br>• If neither of these applies but you are able to provide details of access elsewhere, with or without limitations, please do so in the box below. For example:<br><br>"Data are available from the XXX Institutional Data Access / Ethics Committee for researchers who meet the criteria for access to confidential data."<br><br>"Data are from the XXX study whose authors may be contacted at XXX."<br><br>* typeset	
Additional data availability information:	

Powered by Editorial Manager® and ProduXion Manager® from Aries Systems Corporation

Manuscript Click here to access/download;Manuscript;MB1 Manuscript.pdf

Evolutionary drivers of neophobia across the avian clade

ManyBirds Project*, Rachael Miller[1,2*&^], Vedrana Šlipogor[3,4,28&], Kai R. Caspar[5,6,7&], Jimena Lois-Milevicich[8&], Carl Soulsbury[9&], Stephan A. Reber[10&], Claudia Mettke-Hofmann[11&], Megan Lambert[12*&^], Benjamin J. Ashton[13], Alice M.I. Auersperg[12], Melissa Bateson[77], Solenne Belle[14], Boris Bilčík[15], Laura M. Biondi[16], Francesco Bonadonna[14], Desiree Brucks[17,18], Michael W. Butler[19], Samuel P. Caro[14], Marion Charrier[20], Tiffany Chatelin[20], Johnathan Ching[21], Nicola S. Clayton[1], Benjamin J. Cluver[19], Ella Cochran[22], Francesca Cornero[1], Emily Danby[1], Samara Danel[14,23], Martina Darwich[24], James R. Davies[1], Alicia de la Colina[25], Dominik Fischer[26,27], Ondřej Fišer[28], Florencia Foitzick[8], Edward C. Galluccio[29], Clara Garcia-Co[30], Elias Garcia-Pelegrin[31], Isabelle George[20], Kai-Philipp Gladow[32], Raúl O. Gómez[33], Anna Grewer[34], Katie Grice[35], Lauren M. Guillette[36], Devon C. Hallihan[19], Katie J. Harrington[12], Frauke Heer[26], Chloe Henry[20], Vladimira Hodova[15], Marisa Hoeschele[24], Cécilia Houdelier[20], Paula Ibáñez de Aldecoa[37,38], Oluwaseun Serah Iyasere[18,39], Yuka Kanemitsu[40], Mina Khodadadi[41], Duc Khong[21], Melanie G. Kimball[22], Ariana N. Klappert[42], Lucy N. Koch[12,27], Uta U. König von Borstel[17], Lubor Košťál[15], Anastasia Krasheninnikova[43,44], Lubica Kubikova[15], Connor T. Lambert[36], Daan W. Laméris[30,45], Courtenay G. Lampert[19], Oceane Larousse[46], Christine R. Lattin[22], Zhongqiu Li[47], Michael Lindenmeier[42], Delia A. Lister[48], Julia A. Mackenzie[2], Selina Mainz[49], Danna Masri[22], Jorg J.M. Massen[50,51], Laurenz Mohr[49], Wendt Müller[30], Paul M. Nealen[52], Andreas Nieder[53], Aurèle Novac[54], Nínive Paes Cavalcante[8], Kristina Pascual[77], Carla Pascual-Guàrdia[54], Ayushi Patel[22], Katarína Pichová[15], Cristina Pilenga[55], Laurent Prétôt[56], John L. Quinn[35,57], Elena Racevska[58], Juan C. Reboreda[8], Sam Reynolds[2], Amanda Ruth Ridley[29], Theresa Rössler[12,37], Francisco Ruiz-Raya[59,60], Marina Salas[45], Beatriz C. Saldanha[61,62], Sebastián M. Santiago[8], Nikola Schlöglová[12], Gia Seatriz[21], Eva Serrano-Davies[63], Eva G. Shair Ali[12], Janja Sirovnik[64], Zuzana Skalná[15], Katie E. Slocombe[65], Masayo Soma[40], Tiziana Srdoc[66], Stefan Stanescu[36], Michaela Syrová[28], Alex H. Taylor[67,68,69], Christopher N. Templeton[21,70], Karlie Thompson[52], Sandra Trigo[61,62], Camille A. Troisi[71,72], Utku Urhan[63,73], Maurice Valbert[44], Kees van Oers[63], Alberto Velando[60], Frederick Verbruggen[71], Jorrit W. Verkleij[50], Alizée Vernouillet[71], Jonas Verspeek[30,45], Petr Veselý[28], Auguste M.P. von Bayern[43,44], Eline Waalders[2], Benjamin A. Whittaker[36], Ella R. Williamson[65], Vanessa A. D. Wilson[54,74,75], Michelle Winfield[21], Neslihan Wittek[41], Karen K.L. Yeung[36], Jade A. Zanutto[50,76]

1 Department of Psychology, University of Cambridge, Cambridge, UK
2 School of Life Sciences, Anglia Ruskin University, Cambridge, UK
3 Department of Ecology and Evolution, University of Lausanne, Lausanne, Switzerland

4 The Sense Innovation and Research Centre, Lausanne & Sion, Lausanne, Switzerland
5 Department of Cell Biology, Heinrich-Heine-University Düsseldorf, Düsseldorf, Germany
6 Department of Game Management and Wildlife Biology, Czech University of Life Sciences, Prague, Czech Republic
7 Zoo Frankfurt, Frankfurt, Germany
8 Departamento de Ecología, Genética y Evolución & IEGEBA - CONICET, Facultad de Ciencias Exactas y Naturales, Universidad de Buenos Aires, Buenos Aires, Argentina
9 School of Natural Sciences, University of Lincoln, Lincoln, UK
10 Department of Cognitive Science, Lund University, Lund, Sweden
11 School of Biological and Environmental Sciences, Liverpool John Moores University, Liverpool, UK
12 Messerli Research Institute, University of Veterinary Medicine Vienna, Vienna, Austria
13 College of Science and Engineering, Flinders University, Adelaide, Australia
14 Centre for Functional and Evolutionary Ecology, Univ Montpellier, CNRS, EPHE, IRD, Montpellier, France
15 Institute of Animal Biochemistry and Genetics, Centre of Biosciences, Bratislava, Slovak Republic
16 Instituto de Investigaciones Marinas y Costeras - CONICET, Universidad Nacional de Mar del Plata, Buenos Aires, Argentina
17 Department of Animal Breeding and Genetics, University of Giessen, Giessen, Germany
18 Department of Crop and Animal Sciences, Thaer-Institute of Agricultural and Horticultural Sciences, Humboldt-Universität zu Berlin, Berlin, Germany
19 Department of Biology, Lafayette College, Easton, USA
20 UMR6552-Ethologie Animale et Humaine, Universite´ Rennes1-CNRS, Rennes, France
21 Biology Department, Pacific University, Forest Grove, USA
22 Department of Biological Sciences, Louisiana State University, Baton Rouge, USA
23 Department of Brain and Cognitive Sciences, University of Rochester, Rochester, USA
24 Acoustics Research Institute, Austrian Academy of Sciences, Vienna, Austria
25 Departamento de Conservación e Investigación, Fundación Temaikèn, Buenos Aires, Argentina
26 Zoo Wuppertal, Wuppertal, Germany
27 Raptor Center & Wildlife Zoo Hellenthal, Hellenthal, Germany
28 Department of Zoology, University of South Bohemia, České Budějovice, Czech Republic
29 School of Biological Sciences, University of Western Australia, Perth, Australia
30 Department of Biology, Antwerp University, Antwerp, Belgium
31 Department of Psychology, National University of Singapore, Singapore
32 Department of Animal Behaviour, Bielefeld University, Bielefeld, Germany

33 CONICET-Departamento de Biodiversidad y Biología Experimental, Facultad de Ciencias Exactas y Naturales, Universidad de Buenos Aires, Buenos Aires, Argentina
34 Zoo Krefeld gGmbH, Krefeld, Germany
35 School of Biological, Earth and Environmental Sciences, University College Cork, Cork, Ireland
36 Department of Psychology, University of Alberta, Alberta, Canada
37 Department of Behavioural and Cognitive Biology, University of Vienna, Vienna, Austria
38 Institute for Advanced Study in Toulouse, University of Toulouse 1 Capitole, Toulouse, France
39 Department of Animal Physiology, Federal University of Agriculture, Abeokuta, Nigeria
40 Department of Biological Sciences, Hokkaido University, Sapporo, Japan
41 Department of Biopsychology, Ruhr University Bochum, Bochum, Germany
42 Zoological Research Museum Koenig, University of Bonn, Bonn, Germany
43 Department of Behavioural Neurobiology, Max-Planck-Institute for Biological Intelligence, Seewiesen, Germany
44 Max-Planck Comparative Cognition Research Station, Loro Parque Fundacíon, Tenerife, Spain
45 Antwerp Zoo Centre for Research and Conservation, Royal Zoological Society of Antwerp, Antwerp, Belgium
46 Faculté des Sciences, Université Paris-Saclay, Gif-sur-Yvette, France
47 Lab of Animal Behavior & Conservation, School of Life Sciences, Nanjing University, Nanjing, China
48 Department of Biology, Pittsburg State University, Pittsburg, USA
49 Department of General Zoology, University of Duisburg-Essen, Essen, Germany
50 Department of Behavioural Biology, University of Utrecht, Utrecht, The Netherlands
51 Avifauna Bird Park, Alphen a/d Rijn, The Netherlands
52 Department of Biology, Indiana University of Pennsylvania, Indiana, USA
53 Animal Physiology, Institute of Neurobiology, University of Tuebingen, Tuebingen, Germany
54 Department of Comparative Cognition, University of Neuchâtel, Neuchâtel, Switzerland
55 Zoomarine Italia, Rome, Italy
56 Department of Psychology and Counseling, Pittsburg State University, Pittsburg, USA
57 Environmental Research Institute, University College Cork, Cork, Ireland
58 Zagreb Zoo, Zagreb, Croatia
59 School of Biodiversity, One Health & Veterinary Medicine, University of Glasgow, Glasgow, UK
60 Centro de Investigación Mariña, Universidade de Vigo, Vigo, Spain
61 CIBIO Centro de Investigação em Biodiversidade e Recursos Genéticos, InBIO Laboratório Associado, Universidade do Porto, Vairão, Portugal

62 BIOPOLIS Program in Genomics, Biodiversity and Land Planning, Vairão, Portugal
63 Department of Animal Ecology, Netherlands Institute of Ecology (NIOO-KNAW), Wageningen, The Netherlands
64 Department of Farm Animals and Veterinary Public Health, University of Veterinary Medicine Vienna, Vienna, Austria
65 Department of Psychology, University of York, York, UK
66 Department of Philosophy, University of Vienna, Vienna, Austria
67 Institut de Neurociències, Universitat Autònoma de Barcelona, Bellaterra, Barcelona, Spain
68 ICREA, Barcelona, Spain
69 School of Biological Sciences, University of Canterbury, Christchurch, New Zealand
70 Department of Biology, Western Washington University, Bellingham, USA
71 Centre for Research on Ecology, Cognition and Behaviour of Birds, Ghent University, Gent, Belgium
72 Department of Experimental Psychology, Ghent University, Gent, Belgium
73 Department of Biology, Lund University, Lund, Sweden
74 Department of Comparative Language Science, University of Zurich, Zurich, Switzerland
75 School of Psychology and Social Work, University of Hull, Hull, UK
76 Faculté des Sciences et Ingénierie, Sorbonne Université, Paris, France
77 Biosciences Institute, Newcastle University, Newcastle, UK

* **Corresponding authors**: Rachael Miller: rmam3@cam.ac.uk; Megan Lambert: Megan.Lambert@vetmeduni.ac.at; ManyBirds Project: manybirdsproject1@gmail.com

[&] These authors represent the **ManyBirds Study 1 Leadership team** (wider authorship team listed in alphabetical order)
^ These two authors are the **ManyBirds Project Founders and Project Leads**

Keywords: object neophobia, response to novelty, birds, socio-ecological drivers, big team science, open science, behavioural flexibility

Short title: Neophobia in birds

Abstract

Neophobia, or aversion to novelty, is important for adaptability and survival as it influences the ways in which animals navigate risk and interact with their environments. Across taxa, species and individuals, neophobia is found to vary considerably, but exploring the wider evolutionary drivers of neophobia is hampered by a lack of comparative multi-species studies using standardised methods. Here, we utilised the ManyBirds Project, a Big Team Science large-scale collaborative open science framework, to pool efforts and resources of 129 collaborators at 77 institutions from 23 countries worldwide across six continents. We examined both difference scores (between novel object test and control conditions) and raw data of latency to touch familiar food in the presence (test) and absence (control) of a novel object among 1439 subjects from 136 bird species across 25 taxonomic orders incorporating lab, field and zoo sites. We tested effects of 1) taxonomic order differences; 2) socio-ecological factors including diet, sociality, habitat and range, while accounting for phylogeny; and 3) individual repeatability in object neophobia. We found that at the order level, neophobia was markedly affected by phylogeny. At the species level, using difference scores, species with more specialist diets were more neophobic than those with more generalist diets, providing strong support for the Neophobia Threshold Hypothesis. Migratory species were also more neophobic than non-migratory species, which supports the Dangerous Niche Hypothesis. Finally, we found individual repeatability across time (i.e. across test rounds) and contexts (i.e. controls and novel object tests). Using the largest-scale multi-order study of neophobia to date, our approach and results show that neophobia is a stable behavioural trait predicted by phylogeny. We have identified evolutionary drivers of neophobia across the avian clade and demonstrated the potential of Big Team Science research to reveal broad patterns of bird behaviour.

Introduction

In a ~~constantly~~ changing environment, the ability to adjust behaviour to current conditions is critical for fitness and survival [1]. This ability, known as behavioural flexibility, has been linked to socio-ecological factors, including a species' social organisation and environmental variability [2]. Previous studies suggest that individuals from more socially complex bird, ungulate and primate species show greater behavioural flexibility than individuals from more solitary species [3-5]. Furthermore, birds living in more dynamic environments (i.e. with more pronounced seasonality and resource variability) show greater behavioural flexibility than individuals from more stable environments [6].

Patterns of variation in behavioural flexibility are likely to be driven by both costs and benefits [7] and these are linked to taxon-specific ecological and social factors [8]. Behavioural flexibility is advantageous when animals encounter novelty, as it allows for appropriate responses to new stimuli, food, information and conspecifics [9]. One type of behavioural flexibility is neophobia, an aversion to novelty that is often measured as latency (i.e. time taken) to approach or use novelty (e.g. objects or food resources) compared to familiar stimuli [10]. Neophobic responses affect how individuals interact with changing environments, as they can protect an individual from potential risks, but may also decrease opportunities to exploit novel resources, such as unknown food or nesting sites [11]. Neophobia impacts adaptability and survival [12,13], and can affect ecological processes [14], including invasiveness (cane toads, *Rhinella marina* [15]; common mynas, *Acridotheres tristis* [16]), range and niche expansion (small mammals [17]; Eurasian tree sparrows, *Passer montanus* [18]).

Individuals, populations and species differ in their propensity for neophobia [19,20]. Individuals may show repeatability (i.e. consistency) in their neophobic responses (e.g. mice, *Mus musculus* [21]; ungulates [5]; birds [22,23]) or alternatively, flexibility (e.g. a shift in responses, inconsistency or lack of repeatability, e.g. pinyon jays, *Gymnorhinus cyanocephalus*, and Clark's nutcrackers, *Nucifraga columbiana* [24]). Indeed, neophobia can be plastic, such as when house sparrows (*Passer domesticus*) learn to be less neophobic from conspecifics [25], and rooks (*Corvus frugilegus*) overcome neophobia in groups [26]. Although the drivers of this variability are relatively unknown, there are at least two non-mutually exclusive hypotheses for between-species variation in neophobia. The Neophobia Threshold Hypothesis [27-29] states that neophobia acts as a proximate mechanism for the evolution of niche specialisation, as it changes the probability of a species exploring new stimuli in the environment, thereby shifting its diet breadth or landscape use. This hypothesis is supported by findings that habitat generalists typically demonstrate lower neophobia than habitat specialists [27,28]. The Dangerous Niche Hypothesis [11,30] suggests that neophobia increases with the level of environmental danger and protects against risks, predicting species that utilise unfamiliar or riskier habitats (e.g. higher predator density) will show

higher neophobia. In support of this hypothesis, fish and amphibians from higher predation-risk environments had increased neophobia levels [31]. Furthermore, migration can lead to increased exposure to predation risks and/or unfamiliar habitats [32].

Birds provide an excellent taxon in which to investigate the evolutionary drivers of neophobia. They vary widely in their diet (including foraging behaviour) and sociality [33], thereby making them a suitable group for examining how life history and socio-ecological traits co-vary with neophobia. Previous research on birds has found correlations between neophobic behaviours and dietary breadth [27,28,34,35] and with degree of sociality [19]. Neophobia towards novel objects in 10 corvid species was influenced by urban habitat use, adult social group size, maximum flock size and caching behaviour, though not geographic range, foraging differences or genus [22]. The degree of ecological generalisation has been found to display a negative correlation with neophobia in multiple bird taxa [35,36]. Similarly, sociality can also influence neophobia by moderating risk; for instance, the presence of conspecifics can either enhance or reduce neophobia (e.g. zebra finches, *Taeniopygia guttata,* [19]; common ravens, *Corvus corax* and carrion crows, *Corvus corone,* [37]). In corvids, for example, species living in family groups showed lower object neophobia than those living in territorial pairs [22].

Neophobia has also been linked to birds' ability to exploit novel habitat types such as urban environments. Across more than 8000 bird species, a phylogenetically controlled analysis showed that only 9% of species inhabit urban environments and are characterised by using a broad array of natural habitats [38]. Resources in urban environments may therefore represent an extension of the varied resources that a habitat generalist typically encounters in natural environments. On an individual level, common myna living in urban environments showed lower neophobia towards novel food resources than those living in rural areas [39]. Across 12 (primarily passerine) bird species, individuals were quicker to approach human litter in an urban environment compared to a rural environment [40].

Despite intriguing links between variation in neophobia and different ecological and evolutionary factors, it is unknown how generalisable results are across avian taxa. Few studies have compared neophobia using the same methodology across many species that vary in socio-ecological factors such as sociality and habitat use. Typically, multi-species studies have focussed on closely related species only. For example, a study examining 61 parrot species found that neophobia was positively related to an insect-rich diet and negatively related to a diet of leaves [20] and highlighted that comparative analysis can provide a powerful tool for exploring the evolutionary drivers of behaviour. Other large-scale multi-species studies have been pioneered through Big Team Science collaborative approaches, also known as 'ManyX' projects. Driven by early efforts from psychology to address the replication crisis (Open Science Collaboration; [41,42]), other fields have followed suit, including comparative cognition (ManyPrimates: [43,44]; ManyBirds: [45]; ManyDogs: [46]; ManyManys: [47]). These collaborations

provide large-scale replications of existing studies and allow researchers to ask questions that individual labs do not have the resources to address [48].

Big Team Science collaborations in comparative behaviour and cognition aim to facilitate more diverse species representation and larger sample sizes while granting phylogenetic comparisons that are often lacking in small-scale studies [45,49,50]. For example, in a review of avian cognition and behaviour research from 2015 to 2020, Lambert et al. [45] identified that only ~1% of all bird species were represented in cognitive studies. The subjects represented only four taxonomic orders and originated largely from four countries (UK, USA, Canada and Austria). Most (75%) subjects were from laboratory sites, with field sites and zoos being underrepresented. The present study sought to increase the representation of bird orders, species and individuals to capture wider variation across the avian clade and examine generalisability, as well as include more researchers worldwide.

Using a Big Team Science approach, we aimed to establish whether neophobia is a stable trait within individuals predicted by phylogeny and test predictions of the Neophobia Threshold and Dangerous Niche Hypotheses [11]. We assayed "object neophobia" (following terminology outlined in [51]) using a standardised approach for 1439 subjects across 136 species and 25 taxonomic orders (Fig. 1 and Fig. 2), as Study 1 of the ManyBirds Project (www.themanybirds.com; [45]). Data were collected from labs, zoos and field sites to increase species representation, and because zoos in particular provide access to some rare species less represented in existing literature [52]. We measured latency to touch familiar food presented alongside a novel object and compared this with latency to touch the same familiar food without a novel object present. We analysed both: 1) difference scores (novel object minus control latency values) and 2) raw data (control and test latencies separately). We explored three main research objectives: 1) taxonomic order differences in neophobia; 2) socio-ecological drivers of neophobia at the species level, while accounting for phylogeny (Table 1), and 3) individual repeatability in neophobia. For objective 1, we expected to find variation across bird orders in neophobia, given the vast diversity in social and ecological conditions across the avian clade [45] and previously observed species differences in neophobia (e.g. parrots [20]; corvids [22]).

Table 1. Socio-ecological and other factors assessed for correlations with object neophobia in birds. All factors were coded at species level. For domesticated forms, information on the respective ancestral species from the wild was coded.

Category	Factor	Coding and definition	Data source
**Diet**	**Dietary Breadth**	7 categories (coded: 1-7). Species	Wilman et al.

	(i.e. more feeding specialist versus generalist)	coded on a scale from 1 (one food category consumed; specialist) to 7 (seven food categories consumed; generalist).	[53]
**Sociality**	**Territoriality**	3 categories (coded: non-territorial, seasonally territorial, year-round territorial)	Tobias et al. [54]
	**Social Bond** (i.e. duration of a species' pair-bonds)	3 categories (coded: solitary - no consistent association between partners, short-term - association restricted to a single breeding event/season, long-term - association beyond the previously mentioned duration)	Tobias et al. [54]
**Habitat and range**	**Habitat density**<br>(i.e. structural complexity)	3 categories (coded: 1-3). Structural habitat complexity ranged from 1 (high complexity, e.g. forest / dense shrubland) to 3 (low complexity, e.g. grasslands, deserts, or open water)	Tobias et al. [55]
	**Habitat use**<br>(i.e. more habitat specialist versus generalist)	Occupation of different habitat types (coded: 1-10 types; as defined by BirdLife International (level 1 habitats),<br>species coded on a scale from 1 (one habitat type used; specialist)	BirdLife International [56]

		to 10 (ten habitat types used; generalist).	
	**Anthropogenically modified habitat use** (i.e. altered by human settlements and infrastructure, excluding plantation areas based on their Urban Association Index, UAI)	2 categories (coded: urban avoider or exploiter). The global average UAI, derived from data on 3768 bird species, is 1.14. Species coded as avoiders when UAI below 1.14 and exploiters if above. UAI available for 88 species in our sample. For the remaining 33 species, we relied on additional primary literature and collective expertise of our collaborators to code them.	Neate-Clegg et al. [57]; Sol et al. [58]
	**Migratory habits**	3 categories - migratory (a significant part of the species' populations regularly travels beyond their breeding areas, following a predictable timing and specific routes each season), nomadic (populations are not resident but may constantly move following the availability of sporadic (in time and distribution) resources. In contrast to migratory species, routes and timing of movements cannot be adequately predicted in the long term), non-migratory (movement patterns do not comply to definitions outlined above, resident populations)	IUCN [59]

**Other**	**Body Mass**	Continuous - species' average body mass in grams (g).	Tobias et al. [55]
	**Domestication status**	2 categories - domesticated or non-domesticated	Domestication status reported by respective collaborators

Each factor was coded per species using or informed by peer-reviewed or reputable sources, where available (referenced in Data Source column). Full species coding sheet available in S1 Table.

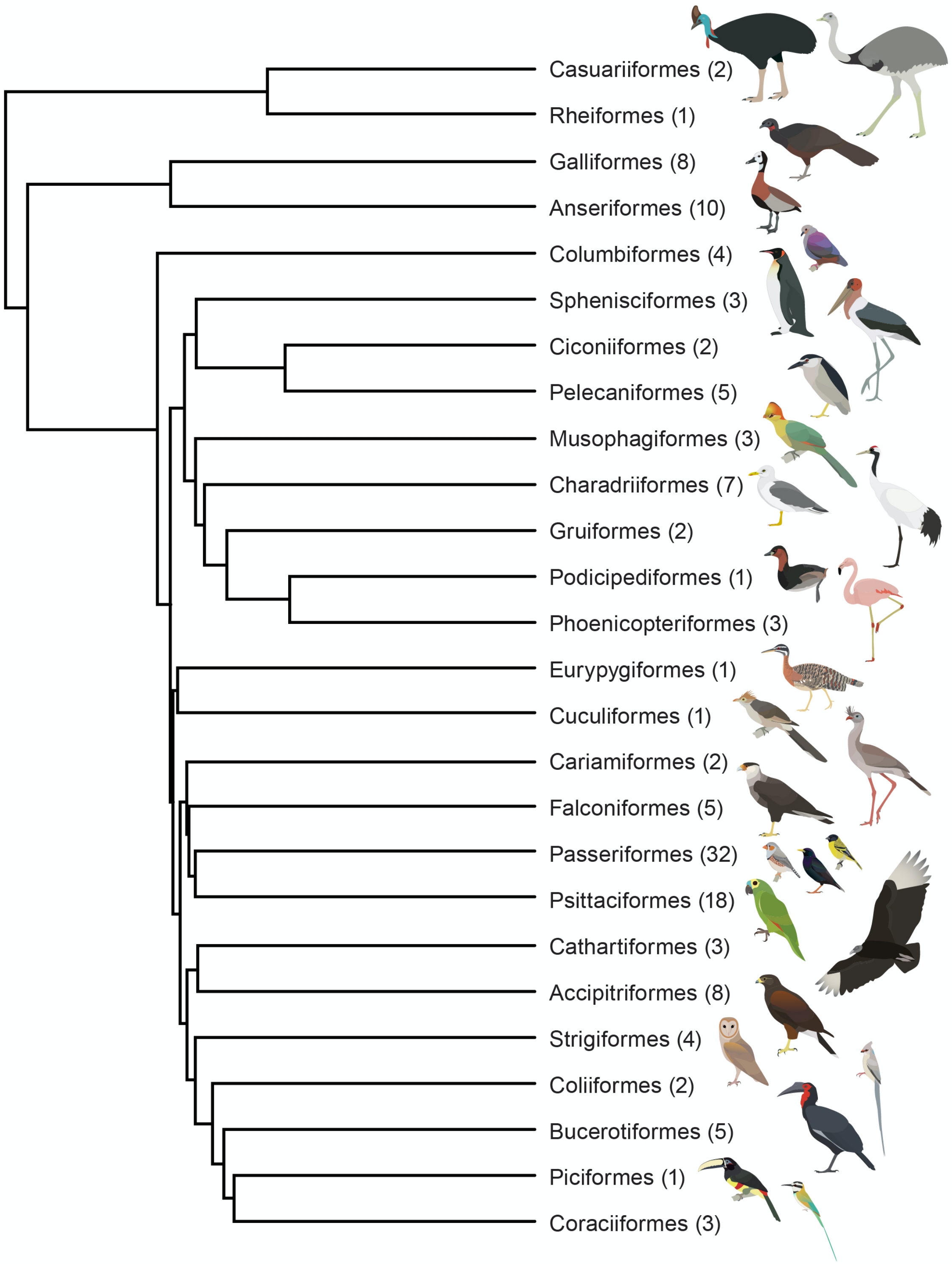

Fig. 1. **Phylogeny of extant bird orders represented in ManyBirds study 1.** Numbers in parentheses indicate the number of species in the given order representing 136 species in total across 25 orders. Phylogeny based on Jetz et al. [60]. Bird drawings created by Raúl O. Gómez (co-author) and shared with permission.

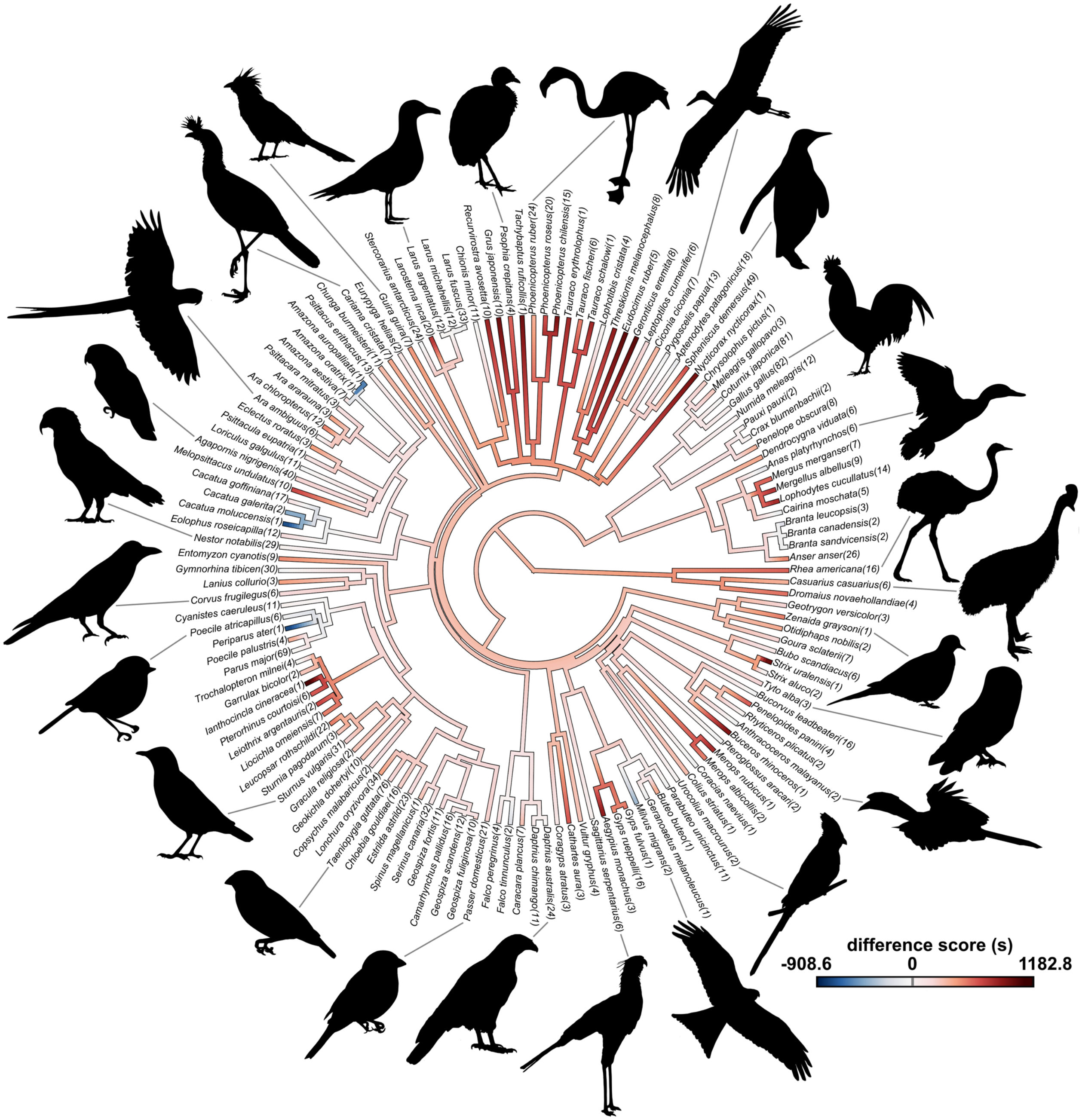

Fig 2. Colour-coded latency difference scores (latency to touch food in novel object condition minus latency in the control condition; measured in seconds) for all species included in the analyses (n = 136). Scores reconstructed for nodes represent maximum likelihood estimates. Sample sizes (total number of individuals and/or flocks tested) are provided in brackets. Silhouettes are in public domain and derive from PhyloPic. The colour-coded tree was created with the *contMap()* function from the *phytools* package [61].

For objective 2, we expected socio-ecological factors, including diet, sociality, habitat and range to influence neophobia, as found in single-species and smaller multi-species studies [3-5]. Specifically, we tested for differences in neophobia between species varying in habitat and range, including feeding and habitat generalists versus specialists. In line with the Neophobia Threshold Hypothesis [27,28], we expected that neophobia would be lower in generalists than specialists, and in migratory rather than non-migratory species, due to increased exposure to a wide variety of novel stimuli and environments. Alternatively, if increased exposure to novelty results in increased risk, we would expect to find the opposite patterns, consistent with the Dangerous Niche Hypothesis [27,28].

We also expected that social factors would influence neophobia, following previous findings in birds and other taxa (e.g. [19,37,62]). For example, territorial species may perceive novelty as a potential threat to their established territories, thereby showing higher neophobia, while non-territorial species may have more fluid or overlapping home ranges and show lower neophobia. Other potential notable influencing factors include body size/mass and domestication. Body size has been linked to the pace-of-life-syndrome and we thus may expect larger birds to have a slower pace of life and be more neophobic [63,64]. Alternatively, larger body size has been linked to lower predation pressure, which may result in lower neophobia in larger species [65]. With domestication, lower reactivity and reduced fear or stress responses are often considered desirable traits among domestic animals, allowing them to cope and reproduce more effectively in human-controlled environments [66-69].

Finally, in addition to exploring the phylogenetic patterns of variation, and testing hypotheses about socio-ecological evolutionary drivers of this variation over time, we wanted to establish the potential for microevolution in contemporary populations by estimating individual repeatability of neophobia, which are a prerequisite for heritable variation. The repeatability of behaviour has been well studied and meta-analysis shows it varies considerably depending on a variety of factors [70]. However, a meta-analysis of neophobia specifically has never been undertaken to this extent. For objective 3, we expected that individuals would be largely repeatable in their neophobic responses, given the relatively short time intervals between different test rounds (~2 weeks), as suggested by a meta-analysis finding larger repeatability in short- than long-term object neophobia studies [51]. Thus, this study enables us to identify the evolutionary drivers of neophobia in the avian clade, and to demonstrate the utility of the ManyBirds Project as a framework for future comparative studies on the evolution of phenotypic variation in birds.

Results

Taxonomic order comparisons of neophobia

Bird taxonomic order affected the latency to touch familiar food in the control (Wald χ^2_{25}=275.79, P<0.001; Fig 3a) and the novel object test conditions (Wald χ^2_{25}=371.46, P<0.001; Fig. 3b). In the test condition, Phoenicopteriformes (flamingos) and Podicipediformes (grebes, represented only by the little grebe, *Tachybaptus ruficollis*) had the longest latencies to touch food (and many did not touch food at all when the novel item was present), whereas Piciformes (represented only by the black-necked aracari, *Pteroglossus aracari*), Falconiformes (falcons) and Galliformes (pheasants and allies) were quickest (Fig. 3b). In the control condition, the longest latencies to touch food were found in Accipitriformes (hawks and allies) and Phoenicopteriformes (flamingos), whereas Falconiformes (falcons), Piciformes and Eurypygiformes (represented by sunbittern, *Eurypyga helias*) were quickest to touch food (Fig. 3a). Overall, we found a difference between control and test latencies across bird orders (Wald χ^2_{25}= 348.46, P<0.001; Fig. 3c), with the biggest overall change between conditions observed (i.e. indicating higher neophobia) in Podicipediformes and Gruiformes (cranes) and the smallest change (i.e. indicating lower neophobia) in Falconiformes, Piciformes and Galliformes (Fig. 3c). The phylogenetic signal (overall test and control conditions models; Pagel's lambda) calculated from the Bayesian GLMM model was 0.64 (0.55/0.71) and for difference scores 0.68 (0.60/0.76), indicating an intermediate phylogenetic signal.

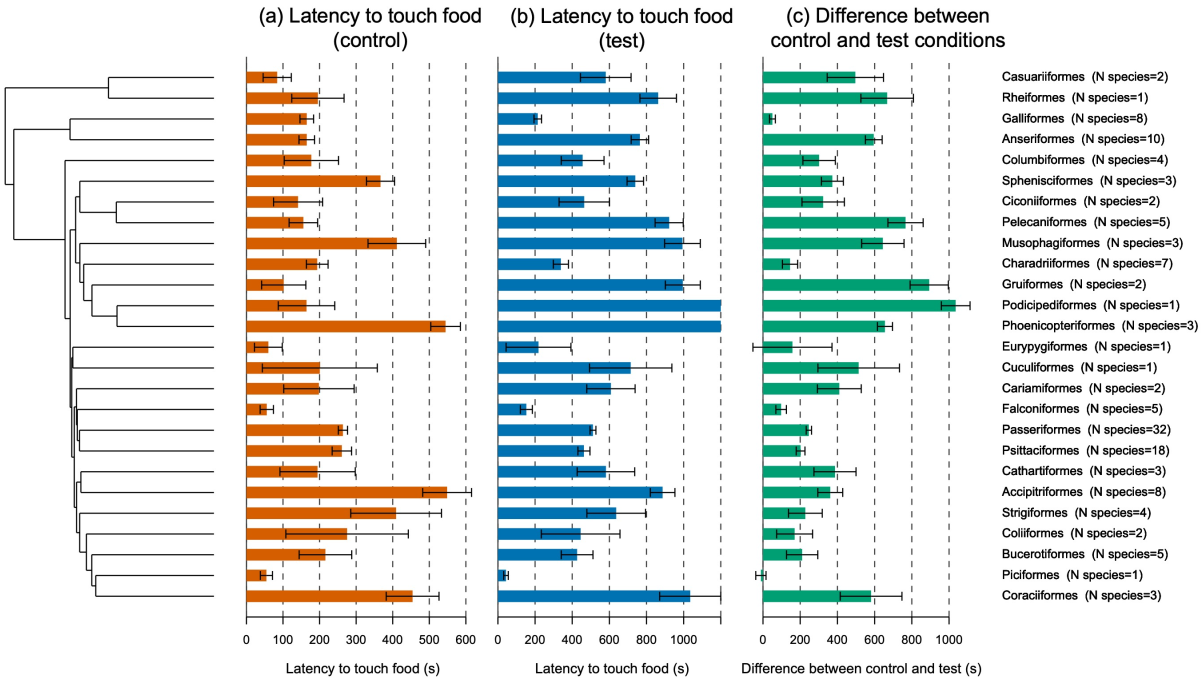

Fig. 3. a, b, c: The mean±SE latency to touch food in (a) control and (b) test conditions, for each taxonomic order. The mean±SE difference (individual difference) between control and test conditions

(c) for each order are shown.

Socio-ecological drivers of neophobia

Across species, using individual difference scores (novel object test values minus control values, i.e. not raw data), dietary breadth and migratory pattern had an effect on neophobia for both Model A, which included the full data set, and Model B, which included non-domesticated species only (Table 2; removing seven domesticated lineages). In line with the Neophobia Threshold Hypothesis, species that fed on fewer dietary categories and were classified as more feeding specialists had higher difference scores (i.e. higher object neophobia) than feeding generalist species with broader diets (Fig. 4). In line with the Dangerous Niche Hypothesis, migratory species had higher difference scores (i.e. higher neophobia) than non-migratory species (Fig. 4).

Table 2. Generalised linear mixed models using Markov chain Monte Carlo estimation methods (MCMCglmm models), testing the effect of predictors on the difference scores (novel object minus control values of latency to touch familiar food).

	**post.mean**	**L. CI**	**U. CI**	**Eff. samp**	p_{MCMC}
	**Model A**				
**Model A (Intercept)**	617.98	-136.49	1384.02	19980	0.108
Body Mass (log)	-6.51	-66.27	51.93	19980	0.832
**Dietary Breadth**	-62.64	-116.26	-7.65	19980	**0.022**
Habitat Use	-5.80	-45.18	33.79	19980	0.777
Anthropogenic Habitat Use (Exploiter)	98.38	-16.31	215.10	19980	0.096
Habitat Density	54.38	-46.67	160.19	19418	0.305
Social Bond (Short-term)	-24.92	-155.69	109.34	19980	0.712
Social Bond (Solitary)	424.36	-540.82	1443.79	19980	0.393
Territoriality (Seasonal)	13.94	-185.19	214.87	20420	0.887

Territoriality (Year-Round)	-92.55	-316.17	144.49	19980	0.431
Migration (Nomadic)	-132.28	-515.02	262.29	19980	0.511
**Migration (Non-migrant)**	-140.36	-277.47	-4.91	20486	**0.045**
Domestication Status	3.07	-220.05	225.84	20217	0.966
	**Model B**				
**Model B (Intercept)**	823.52	67.18	1585.75	19980	**0.035**
Body Mass (log)	-33.23	-95.60	29.14	19546	0.293
**Dietary Breadth**	-64.25	-116.37	-10.92	19980	**0.017**
Habitat Use	-20.19	-61.70	23.13	19980	0.347
Anthropogenic Habitat Use (Exploiter)	67.89	-51.36	193.86	19980	0.271
Habitat Density	91.73	-22.33	204.05	19980	0.111
Social Bond (Short-term)	-1.42	-140.57	124.51	19980	0.983
Social Bond (Solitary)	446.14	-489.09	1447.20	19980	0.364
Territoriality (Seasonal)	-5.77	-215.36	207.56	20489	0.957
Territoriality (Year-Round)	-109.79	-356.61	131.60	20939	0.376
Migration (Nomadic)	-159.51	-552.18	242.40	19980	0.423
**Migration (Non-migrant)**	-144.28	-285.34	-3.24	19980	**0.044**

Individual ID, nested within site, and phylogeny were included in the model as random effects to control for non-phylogenetic and phylogenetic non-independence, respectively, among individuals. Model A includes all data, whereas Model B contains only non-domesticated species. p_{MCMC} is twice the posterior probability that the estimate is negative or positive (whichever probability is smallest), L. CI = lower 95% credible interval, U. CI = upper 95% credible interval. For migratory patterns, the 0 intercept in this 3-level factor is migratory species. Significant predictors are indicated in bold. Eff. samp is the effective sample size of a Monte Carlo computation.

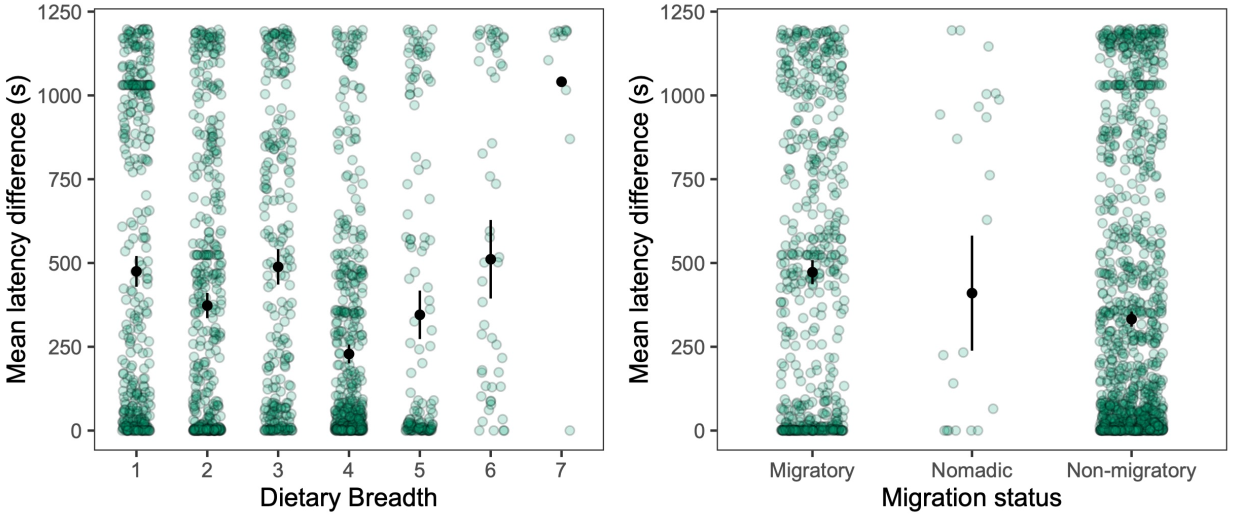

Fig. 4. Mean±SE latency on the mean latency difference scores (novel object minus control values) to touch familiar food in relation to a) dietary breadth and b) migratory pattern. A higher difference score indicates a (higher) neophobic response to novel object presence. Circles represent individual difference scores. For a) dietary breadth, note that categories 1 to 5 represent 133 species, thus driving this effect (categories 6 and 7 contain two and one species, respectively).

Using raw data (control and test latencies separately), the addition of a novel object overall increased the latency of birds to touch familiar food (Table 3). Neophobia conditions interacted with 1) dietary breadth, 2) migratory patterns, 3) body mass, 4) social bond and 5) domestication status. The dietary breadth and migration patterns findings reported for the difference scores (Fig. 4) were also recorded for the raw data (Fig. 5 and Fig. 6). No effects of territoriality, habitat density, habitat use or anthropogenically modified habitat use were detected. Therefore, under novel object conditions, species that were more restricted (versus broader) in dietary breadth (Fig. 5), migratory (versus non-migratory; Fig 6), heavier in body mass (versus lighter; S1 Fig), solitary (versus social; S2 Fig) and non-domesticated (versus domesticated) showed higher object neophobia - i.e. they took longer to touch familiar food in the presence of novel objects (Table 3). Under control conditions, migratory (versus non-migratory; Fig 6) and with lower body mass (versus heavier) species took longer to touch familiar food when a novel object was not present (S1 Fig; Table 3).

Table 3: Generalised linear mixed models using Markov chain Monte Carlo estimation methods (MCMCglmm models), testing the effect of predictors on the latency to touch familiar food when no novel object was present (i.e. raw data).

	post.mean	L. CI	U. CI	Eff. samp	p_{MCMC}
(Intercept)	95.78	-483.97	650.32	18638	0.737
**Condition (test/control)**	225.18	84.34	371.81	19980	**0.002**
Body Mass (log, grams)	-0.46	-45.49	43.31	19980	0.980
Dietary Breadth	3.71	-36.40	45.12	19980	0.854
Habitat Density	-1.31	-78.49	75.59	19980	0.965
Anthropogenic Habitat Use (Exploiter)	10.46	-78.88	101.25	19980	0.828
Habitat Use	16.75	-12.01	47.82	19980	0.272
Social Bond (Short-term)	97.59	-5.01	203.05	19980	0.068
Social Bond (Solitary)	145.11	-589.06	889.27	19980	0.702
Territoriality (Seasonal)	-68.45	-211.45	85.93	19980	0.358
Territoriality (Year-Round)	-24.79	-198.29	144.65	20474	0.783
Migration (Nomadic)	233.80	-55.84	527.49	21142	0.118
Migration (Non-migrant)	-65.52	-170.85	35.63	20108	0.217
Domesticated	52.71	-117.58	221.29	19466	0.542
**Condition x Body Mass**	15.95	5.74	26.46	19980	**0.004**
**Condition x Dietary Breadth**	-47.05	-65.33	-28.97	19980	**<0.001**
Condition x Habitat Density	24.75	-8.84	59.46	20821	0.156
Condition x Anthropogenic Habitat Use (Exploiter)	-14.34	-63.99	31.10	20443	0.557
Condition x Habitat Use	4.37	-7.97	16.82	19980	0.492
Condition x Social Bond (Short-term)	-29.48	-81.37	21.23	19980	0.265
**Condition x Social Bond (Solitary)**	486.23	260.27	712.80	19980	**<0.001**
Condition x Territoriality (Seasonal)	46.19	-18.60	112.45	21474	0.170

Condition x Territoriality (Year-Round)	-63.92	-132.43	-0.25	21148	0.058
Condition x Migration (Nomadic)	-37.93	-197.71	115.25	19422	0.643
**Condition x Migration (Non-migrant)**	-107.59	-164.76	-48.39	19980	**<0.001**
**Condition x Domesticated (Domesticated)**	157.37	106.06	210.54	19991	**<0.001**

Individual ID was nested within the study site, and species and phylogeny were included in the model as random effects to control for non-phylogenetic and phylogenetic non-independence, respectively, among individuals. p_{MCMC} is twice the posterior probability that the estimate is negative or positive (whichever probability is smallest), L. CI = lower 95 credible interval, U. CI = upper 95 credible interval. Significant predictors are indicated in bold. Eff. samp is the effective sample size of a Monte Carlo computation.

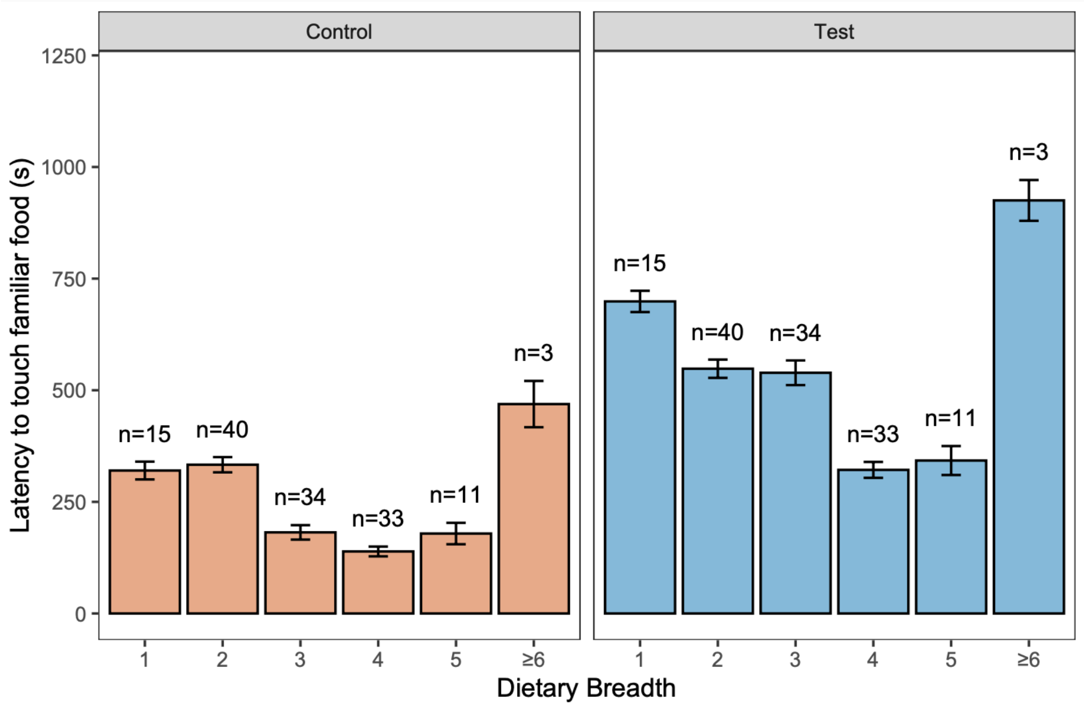

Fig. 5. Mean±SE latency to touch food in control (orange) and novel object (blue) conditions, in relation to dietary breadth, the number of food categories consumed. Species sample sizes for each dietary breadth category are given above the bars.

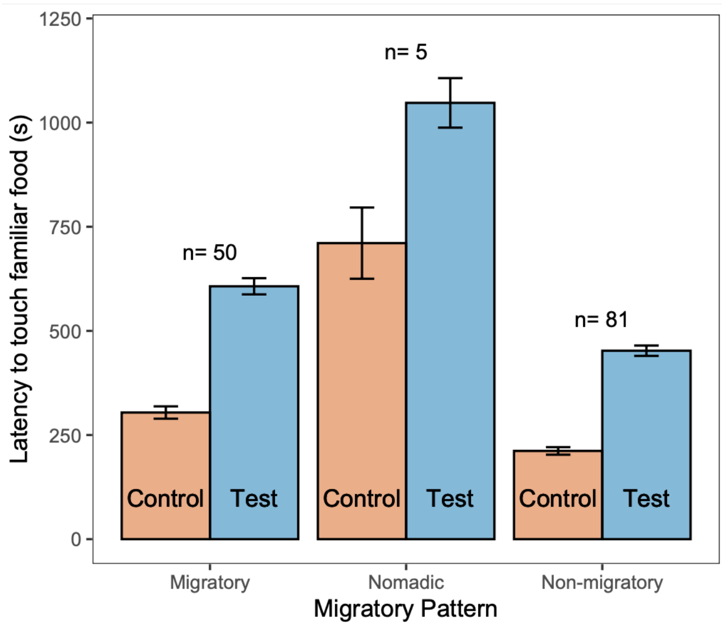

Fig. 6. Mean±SE latency to touch food in control (orange) and test (blue) conditions, in relation to migratory pattern. Species sample sizes for each type of migratory pattern are given above the bars.

Focusing the analysis on a reduced data set of non-domesticated species only (i.e. excluding seven domesticated lineages; raw data across species) did not change the findings on dietary breadth, social bond or migratory pattern (S3 Table). However, there was no longer an effect of body mass on latency to touch food in control conditions, and instead there were interacting effects of condition and two habitat variables (habitat density and habitat use). Specifically, species from less complex habitats took longer to touch food in both control and test conditions than those from more complex habitats (habitat density; S3 Table; S3 Fig). Species found in fewer habitat categories (i.e. more habitat specialists) had longer latencies to touch food under test conditions than species found in more habitat categories (i.e. more habitat generalists) (habitat use; S3 Table; S4 Fig). There was also a non-significant trend for urban avoider species to have longer latencies to touch food than urban exploiter species under test conditions (anthropogenically modified habitat use; $p_{MCMC} = 0.067$).

Individual repeatability

Individuals' responses to novel stimuli were temporally repeatable across test rounds (1–2) and contextually repeatable across control and novel object test conditions (repeatability estimate: R= 0.443 [0.41/0.48], p<0.001; raw data). In addition, individual responses were temporally repeatable within each condition (control: R= 0.429 [0.38/0.48], p<0.001; novel object test: R= 0.521 [0.48/0.56], p<0.001).

Discussion

In the largest multi-species standardised comparative study on neophobia, conducted through the ManyBirds Project, we aimed to establish whether neophobia is a stable trait within individuals predicted by phylogeny and to identify the evolutionary drivers of neophobia across the avian clade. In line with our predictions, we found that object neophobia related to 1) phylogeny, 2) socio-ecological factors relating to diet, sociality, habitat and range, as well as domestication status and body mass, and 3) was individually repeatable across time and context. Specifically, latency to touch familiar food in the presence of a novel object varied across the 25 bird orders represented. Across all 136 species tested, using difference scores (control latencies subtracted from novel object latencies), we found that species with a more restricted dietary breadth and migratory species were more object neophobic than species with a broader dietary breadth and non-migratory habits, respectively. Using raw data, neophobia was more pronounced among species that were feeding specialists, migratory, heavier in mass and non-domesticated than among species that were feeding generalists, non-migratory, lighter in mass and domesticated. When we removed seven domesticated lineages from the sample, most of these findings also held for non-domesticated species, and habitat factors became important. Specifically, species from less complex habitats and that were more habitat specialists were more neophobic than species with more complex habitats or more habitat generalists.

Socio-ecological drivers of neophobia

Diet

We expected feeding generalists to be less neophobic than specialists, as they are usually more likely to forage in unfamiliar situations and interact with a wider range of stimuli than specialists [27,71,72]. Indeed, feeding generalists are typically faster to explore and exploit different food types [20,27,73,8]. Our findings, based on both raw data and difference scores, support this prediction as specialists were found to exhibit higher levels of neophobia, and neophobia decreased with generalism. Specialised species experience fewer variations in environmental conditions and as a consequence may perceive changes as more threatening than generalists. We note that only three of the included bird species had very high dietary breadths, receiving classifications of 6 and 7, therefore, the trend was driven by the 133 species with dietary breadth 1-5.

Sociality

We expected but did not find differences between species with short- versus long-term social bonds, related to the flexibility of social structures in these bonds (see S2 Fig for further discussion of

social bond findings). We also expected territoriality to play a role, whereby territorial species might be more likely to perceive new stimuli in their territory as a threat (as in corvids; [20]), but did not find evidence to support this. If territoriality does generally covary with neophobia, then potentially our assay does not reflect representative stimuli. However, our findings are in line with a study on pigeons and doves that showed that social organisation did not affect neophobia [74].

Habitat and Range

Migratory species were more neophobic than non-migratory species in both analyses. This finding may relate to increased risk associated with interacting with a wide range of novel items and environments, or increased predation risk that migratory species may encounter. Increased neophobia may be beneficial in species that encounter more unfamiliar sites, as migratory species only stay for short periods in one particular area, while non-migratory species are more familiar with changes and threats in their territorial range throughout the year [30,75]. This is also in line with some previous studies in closely related species; for instance, neophobia was higher in the migratory garden warbler (*Sylvia borin*) than in the resident Sardinian warbler (*Curruca melanocephala*) [75], and in migratory New World blackbirds (Icteridae) than in resident species of the same taxonomic family [30].

In addition, we expected generalist versus specialist habitat use and habitat density to affect neophobia. Using raw data and focusing on non-domesticated species only, species with less complex habitats and that used fewer habitat categories (i.e. more specialist species) were more neophobic than species with more complex habitats and that used more habitat categories (i.e. more generalist species). Anthropogenic habitat use was examined separately, and these habitats may be a special case of complex habitats, due to typically higher predictability and food abundance. In these habitats, neophilia (i.e. attraction to novelty) may be more adaptive for survival. However, the association between neophilia and neophobia in urban environments has not been adequately investigated [76]. In our study, we found no effect of anthropogenically-modified habitat use on object neophobia (note that the defined ‘anthropogenically modified habitat’ is not synonymous with ‘urban habitat use’), but there was a trend for more neophobia in urban avoiders, which was consistent with some previous findings (e.g. house sparrows [6]; corvids [20]). This finding did not appear to be due to a lack of representation of urban exploiters, which represented 63% of species in our sample. However, we classified anthropogenic habitat use in birds into only two categories, which may have limited the resolution of our analysis.

Other factors

We included domestication status and body mass as other factors that may influence neophobia. Within species, higher predation risk can result in lower body mass (lizards [77]) and neophobia can

increase with changes in predator diversity (fish [78]). We found that, in the novel object test condition, heavier species were more neophobic - a finding that requires further investigation to, for instance, explore the relation to species differences in predation risk, or alternatively linked with the pace-of-life syndrome [63]. Domesticated species were less neophobic than non-domesticated species, which was expected since neophilia is often favoured by the domestication process, or by the indirect effects of selective breeding of certain individual characteristics, like reduced reactivity, on behaviour more broadly [69] .

Neophobia Threshold and Dangerous Niche Hypotheses

We found evidence both in support of the Neophobia Threshold Hypothesis, including effects of dietary breadth and habitat use, and the Dangerous Niche Hypothesis, including an effect of migratory pattern [27]. For instance, the finding that habitat generalists showed lower neophobia than habitat specialists is consistent with predictions of the Neophobia Threshold Hypothesis [25,26]. To our knowledge, this is one of few studies to investigate these two hypotheses at the same time, and the only study to do so in such a large sample size. In relation to a previous related study, Miller et al. [20] identified four socio-ecological correlates of object neophobia in 10 corvid species. Although the examined factors differed between this and the corvid study, we similarly found effects that were broadly related to diet, sociality, habitat and range. However, because the tested corvid taxa have largely similar diets, degree of dietary specialisation could not be examined relative to neophobia as it could in the present study. Furthermore, given the broader representation of species and orders in this study, we were able to control for phylogeny in analyses, which allowed us to test the broader evolutionary drivers of neophobia.

Individual repeatability

Individual responses to familiar food and to familiar food in presence of novel objects were repeatable over two test rounds. In previous studies, individuals either showed consistency or flexibility (e.g. shift in responses, inconsistency or lack of repeatability) to novelty [22,79]. This variation in neophobia repeatability may be due to a range of factors, including developmental and social influences [21,24,80]. We used a consistent methodology across species, and while subjects varied in source (e.g. wild versus captive) and whether they were tested socially versus alone, this was held constant within individuals. Our results suggest that neophobic responses reflect a stable behavioural trait that is consistently different among individuals, which may reflect underlying individual differences in neurobiology or endocrinology ([81,82]) and is a prerequisite for heritable variation.

Limitations and future directions

Large-scale collaborations provide multiple advantages [47,50] but also pose challenges, such as increasing the amount of uncontrollable variance in the data set through differences in testing conditions based on site-specific factors (e.g. social versus testing alone; test area size) and the prior history of study subjects (e.g. whether they were wild or captive-bred; subject age) [83]. For instance, although we were open to including neophobia data from free-living animals, 90% of our subjects were sampled from lab and zoo captive settings rather than field settings, which may have introduced potential biases (see below). We controlled for this variance wherever possible in the analyses, by including factors like domestication status and by analysing difference scores as well as raw data. We also provided as many details as possible for each study site in S4 Table, allowing for possible future investigations into the effects of site-specific factors not considered in the present analyses.

Regardless of the inclusive approach taken to sample selection and authorship, some avian taxa were substantially more represented than others, due to increased access to those species rather than *a priori* species selection. Therefore, some factor coding categories were somewhat unbalanced, such as social bonds, where solitary birds are represented by one species only (S2 Fig). We remain conservative in our interpretation in these instances, and care should be taken with wider generalisation of these findings. Even with a clear and relatively simple protocol, ensuring that all data were collected in as comparable a manner as possible was a challenge. All collaborators were required to submit pilot videos of their testing conditions and photograph examples of their novel objects for approval by the leadership team prior to data collection (e.g. to check camera angles and positioning of familiar foods and objects). Novel objects were required to follow the same approximate principals regarding colours and textures and were adjusted for species size. In field or some zoo settings, it was difficult to identify individuals; in these cases, individual data points were not included in the repeatability analysis or were only tested in one round. As it has been found that captivity and seasonality can influence neophobia [84], other limitations of this study include the potential influence of captive living, which includes variation in subject source and rearing conditions, and testing across different seasons or breeding stages of subjects.

To facilitate standardisation by multiple researchers across many species and sites, we focused on object neophobia only. Future studies may expand to other types of neophobia, such as novel foods, predators and environments [84]. We have focussed primarily on ultimate explanations for neophobia [85] but there are many potential proximate drivers of neophobia that were not possible to assess with this study and sample, such as age effects [86], whereby younger birds may be less neophobic than adults, [37,87], and hormone effects, whereby higher levels of prenatal testosterone and lower levels of adult corticosterone are often linked with lower neophobia [88-91]. Future studies could also further examine wild versus captive differences both within and between species, and the relation between neophobia and

other cognitive and behavioural measures, such as problem-solving/ innovation [29]. Available evidence suggests that captive bird populations may differ drastically from wild ones regarding various aspects of behaviour and cognition [92], placing an important caveat on studies that, just like ours, rely heavily on captive-bred subjects from zoos and laboratories. Future ManyBirds projects may include more targeted species selection, as well as increasing the number of individuals per species as appropriate for the specific research question, with a planned consensus-based approach for study selection.

Conclusion

Neophobia, and behavioural flexibility more broadly, plays a role in adaptability and can influence survival [2]. It is therefore a behaviour of interest and importance for both fundamental and applied research questions, such as understanding how animals respond to new and changing environments, and it is crucial for informing conservation actions like species reintroductions [93,94]. This large-scale collaborative study through the ManyBirds Project enabled a standardised approach to testing object neophobia across many species and orders across the avian clade. We established that neophobia is a stable behavioural trait predicted by phylogeny and identified major socio-ecological drivers relating to diet, sociality, habitat and range that support both the Neophobia Threshold Hypothesis and Dangerous Niche Hypothesis. Further, these drivers of neophobia in birds are found in other taxa, like mammals [5]. We identified several avenues for future research, including a focus on both ultimate and proximate causes of neophobia.

Materials and Methods

Subjects, Species and Data Collection Sites

This study aimed to include data from as many bird species available as possible, across labs, zoos and field sites with an open call for data contributions from January 2022 to April 2023. This study included 1439 subjects and 136 species, across 25 taxonomic orders (Fig. 1 and Fig. 2; S1 Table). The taxonomy and nomenclature of the Clements checklist of Birds of the World [95] was applied. We included as many subjects as possible, with no limitations on bird sex or age. It was useful, though not essential, for individuals to be identifiable (e.g. via leg rings, wing bands, cable ties, plumage colouration, or body size differences) and to be able to be tested while alone (e.g. temporary visual isolation). The majority of data collected constitutes new data, i.e. it was collected for this study. Some data were collected using the same protocol and published separately. Specifically, we included a dataset on

neophobia in five hornbill species (genus *Bucerotidae*) [52] and in Bali myna, *Leucopsar rothschildi* [94] with permission or collaboration with relevant authors. Finally, we included some additional published and new data (dataset available via Figshare 10.6084/m9.figshare.27324972), but excluded these data from analysis due to having a maximum trial length of 600 seconds per trial, shorter than other trials (previously published data on 10 corvid species, [22], as well as new data on four species - rock dove, *Columba livia,* Japanese quail, *Coturnix japonica,* budgerigar, *Melosopittacus undulatus* and zebra finch, *Taeniopygia guttata*).

Materials

There were two experimental conditions: control (familiar food alone) and novel object test (familiar food placed beside the novel object). The familiar food, placed in a familiar food bowl, was species-appropriate and varied between bird groups, depending on the regular diet from each site. There were one to three types of novel objects (depending on number of test rounds conducted per site and species) with the same visual novelty properties (i.e. not auditory or olfactory novelty). Novel objects were made of multiple items and textures, with no part that could look like eyes (to avoid resembling predators), and all contained the colours blue, yellow, green, and red [79]. Parts of the objects were also shiny. The objects were between one third and one half of the subject's size, so that the object size varied with species (Fig. 7). Objects were constructed by the primary collaborator with a photograph shared for checking and approval by the ManyBirds study 1 team prior to data collection commencement. Where possible, the birds were tested in a familiar area (e.g. the regular feeding location) or following necessary habituation prior to testing. The test sites gave the birds as much room as possible to avoid or approach stimuli. In a few rare cases, for five zoo-housed species at Basel Zoo (kea, *Nestor notabilis,* gentoo penguin, *Pygoscelis papua*, king penguin, *Aptenodytes patagonicus*, African penguin, *Spheniscus demersus*, Southern ground-hornbill, *Bucorvus leadbeateri*), the novel object was placed in a transparent box to prevent the birds from touching it, a zoo-specific requirement. These birds were thus given additional trials presenting the empty box without a novel object beside food to habituate them to the presence of the box.

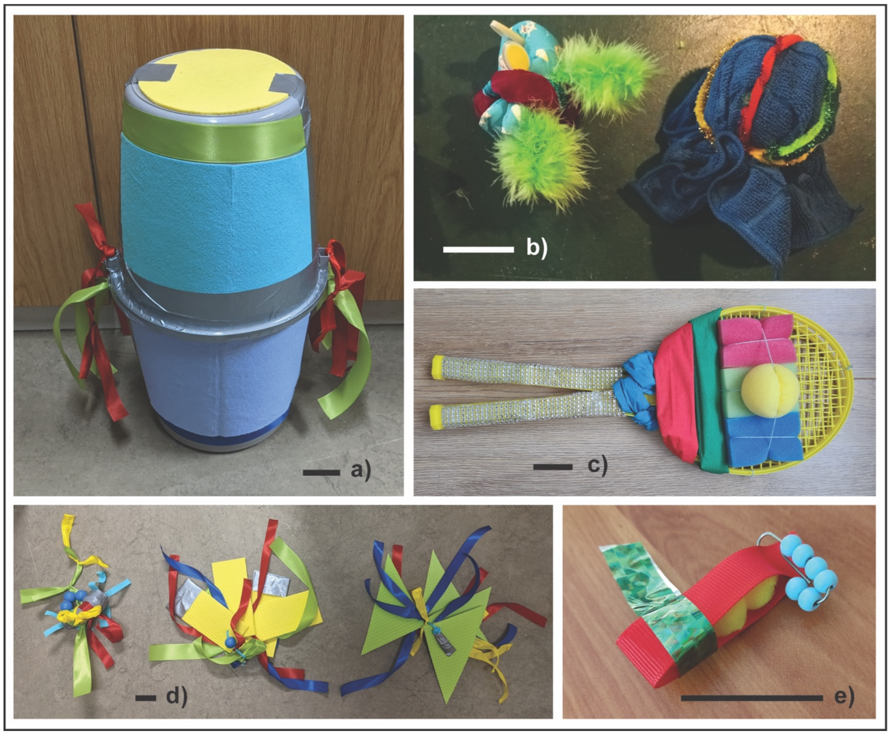

Fig. 7. Examples of novel objects used. Each black/white bar is 5 cm long. The objects ranged in size from a third to half the size of the subjects: a) Southern cassowary, *Casuarius casuarius*, b) Moluccan eclectus, *Eclectus roratus*, c) Rüppell's vulture, *Gyps rueppelli*, d) grey-winged trumpeter, *Psophia crepitans*, e) common waxbill, *Estrilda astrild*.

Procedure

The tests involved measuring behavioural responses to novel objects placed beside familiar food. The controls involved measuring behavioural responses to familiar food only. Testing occurred either a) in the presence or b) absence (i.e. temporary visual separation or isolation) of conspecifics, with 50% of subjects tested socially (i.e. conspecifics/hetero-specifics present) and 50% of subjects tested alone. For captive subjects, presence or absence of others was consistent within individuals and sites. Where separation was not possible, the number of test stimuli (i.e. novel objects present per trial) and familiar food items were increased respectively to the number of subjects. For example, if two subjects were present, two separate test sites were offered, each site with a familiar food bowl and a novel object. Although species were not typically food deprived in a standardised manner (because this was not possible for subjects in the field sites), testing took place either in the morning or alongside the usual main daily feeding (i.e. first or main feed of the day for captive subjects) to ensure that birds showed similar hunger and motivation levels for obtaining food.

The novel object was placed beside the familiar food-filled dish (distance: ~20 cm for larger

species, such as Madagascar ibis, *Lophotibis cristata*; ~10 cm for smaller species, such as smaller passerines), with items placed in the same location (e.g. a table/platform/mesh wall – large enough so that the bird could approach it slowly from more than a body length away) for all tests and individuals within each species. The stimuli were either presented before the subject entered the testing area or introduced together with the food while the subjects were present. The test trial started when the subject entered the testing area, or the experimenter left the test area. Trial length was determined by a pilot control trial with familiar food only to check when each individual/species would usually approach their daily feed. This was expected to be a maximum of 20 minutes based on previous research (e.g.[94]). Each trial ended after the maximum trial length in order to code all variables and all individuals but only if it was appropriate for the given site or species tested. For example, if an individual was temporarily separated from conspecifics for testing, the full trial length only ran provided the individual was not stressed by this extended separation time. Whenever it was not possible to reach the maximum trial length, trials ended when the subject touched the familiar food (i.e. with beak or body) (this occurred at 24 sites; S4 Table).

Each test round consisted of one novel object test and one control, a total of two trials. Every test round was conducted between one and three times (1 trial per condition per round, 6 trials in total) - the latter allowed for individual repeatability testing. For example, only one test round was conducted in cases where individuals were not individually identifiable (e.g. some zoo and field sites), as the individual-level responses could not be accurately compared between rounds. The control trial was conducted within 48 hours of the novel object test trial to ensure that hunger/motivation levels were as comparable as possible between the conditions. Test rounds were repeated after approximately 2 weeks, e.g. week 1: control-object, week 3: control-object, week 5: control-object. Therefore, testing took between 1 and 6-8 weeks to complete per species and site.

At sites where more than one type of novel object was created for testing (i.e. more than 1 test round run), the same type of novel object was used for test round 1 across all individuals, to ensure that test round 1 was comparable between sites. Exceptions were tests of blue-faced honeyeaters (*Entomyzon cyanotis*; *n* = 5), Fischer's turaco (*Tauraco fischeri*; *n* =3), Marabou storks (*Leptoptilos crumenifer; n* = 2), Southern cassowaries (*Casuarius casuarius; n* = 2), and Visayan hornbills (*Penelopides panini*, *n* = 2) at Zoo Frankfurt, where the presentation order of novel objects was fully randomized. The order of presentation of the novel objects as well as the object types in test round 2 and 3 was, however, counterbalanced across subjects and species, e.g. subject 1, round 1 – novel object type 1, round 2 – type 2, round 3 – type 3; subject 2, round 1 – type 1, round 2 – type 3, round 3 – type 2, etc. The testing schedule for half of the subjects was control-object in every round and for the other half object-control in every round per group (Table 5).

Table 5. Example test schedule and object ("obj") counterbalancing across subjects.

Week	Day	Round	Bird 1	Bird 2	Bird 3	Bird 4	Bird 5	Bird 6
1	1	1	Obj 1	Obj 1	Obj 1	Control	Control	Control
	2*		Control	Control	Control	Obj 1	Obj 1	Obj 1
4	1	2	Obj 2	Obj 3	Obj 2	Control	Control	Control
	2*		Control	Control	Control	Obj 3	Obj 2	Obj 3
6	1	3	Obj 3	Obj 2	Obj 3	Control	Control	Control
	2*		Control	Control	Control	Obj 2	Obj 3	Obj 2

*Or day 3 (within 48 hours of first presentation) and was consistent across all subjects for the duration of the study. Note that all subjects were tested with the same object in Round 1.

Our main measure assessed was latency to touch familiar food, which signifies how long the subject took to touch a familiar, desirable food in the presence of a novel object (during test trials) or without novel objects (during control trials). Any avoidance of the novel object (and thus familiar food) was then interpreted as neophobia [11]. We used latency to touch familiar food rather than latency to eat familiar food to control for any potential bias in terms of whether the bird swallowed the food. Example video trials can be found at: https://youtu.be/xGPQ6lcRGpE. Pre-registration with protocol and analysis plan was published in January 2022: https://osf.io/vdbks/?view_only=3d1a68898a0145c0b0f9c5a1b7b30333 .

Data analysis

Socio-ecological and other factor coding

As outlined in Table 1 (Data Source column) and via the full species list (socio-ecological coding in S1 Table**),** socio-ecological and other factors were coded for each species using or informed by peer-reviewed or reputable sources, where available. The selection of these factors was determined by the final species representation in the dataset, the reliability of coding each factor (e.g. available peer-reviewed sources) and were hypothesis driven.

Filming and coding

All trials were video recorded with high resolution cameras. Camera position was the same across trials, at least within the same subject. The familiar food and, in the test trials, the novel object were visible on screen at all times. Recordings were coded frame by frame, but at least with a sampling frequency of 1.0 frame per second. Coding software included Microsoft Excel, Solomon Coder (https://solomoncoder.com) or BORIS [96]. Prior to data collection, each primary collaborator submitted a pilot video (familiar food only) to the Study 1 leadership team for approval. The start of the trial was clearly denotable and, although it varied between different sites depending on species and setup, was consistent between trials. For instance, this was either when i) the animal entered the testing area by itself, ii) the animal was released into a testing cage or iii) the experimenter left the test area. Under the responsibility of each primary collaborator, 12% of video trials were coded, for each species and enclosure, by a second coder and checked to ensure significant and sufficiently strong inter-rater reliability (i.e. min of 0.8) prior to coding the remaining data (S2 Table).

We considered both individual birds and "flocks" for the analyses. Flocks were only coded when the individual identification of a bird within a group was ambiguous. In that case, the first interaction of any individual within the flock with the familiar food (or novel object) was coded. Hence, latency to approach/touch food/novel objects were not necessarily determined from the same individual across different trials. For the analyses, flock-related data were dealt with analogously to those from individual birds. Thus, data from a flock were weighted as if they corresponded to a single bird. In total, flocks were coded for nine species. In five of those species only, all data came from flocks (speckled mousebird, *Colius striatus*, Griffon vulture, *Gyps fulvus*, carmine bee-eater, *Merops nubicus*, black-crowned night heron, *Nycticorax nycticorax*, blue-naped mousebird, *Urocolius macrourus*). In the other four species (Cinereous vulture, *Aegypius monachus*, white stork, *Ciconia ciconia*, white-faced whistling duck, *Dendrocygna viduata*, Inca tern, *Larosterna inca*), data from flocks and individual birds (i.e. where it was possible to identify individuals consistently) were pooled.

Statistical analysis

For all between-species and order comparisons, we tested the full data set using raw data (control and test latencies separately) and difference scores with the same model parameters and both fixed and random effects, with interaction with neophobia conditions (control/novel object test) (Objective 1 and 2 below). Difference scores, i.e. latencies of control responses (only familiar food) subtracted from the latencies of novel object tests aimed to help in standardising latencies across sites as well as control for baseline

neophobia and current motivational state [11,22]. Raw data analyses tested for effects within each condition, i.e. within novel object and control trials, while difference scores used a composite score across conditions. For within-species comparisons at the individual level (Objective 3 below), only raw data were used (as per [22]). For each objective, we carried out analyses in R version 4.3.1 [97]. Given the nature of the dataset, e.g. testing multiple individuals per species at multiple sites, we used mixed models (Objective 1) or Bayesian mixed models (Objective 2) to account for complex random effects in phylogenetic dependence (Objective 2). In the following paragraphs, we outline the statistical analysis.

Objective 1 was a taxonomic order comparison of neophobia: that is, we tested whether “order” was a predictor of the obtained data. The response variable was “latency to touch familiar food”, with the main effect of “order” and the random effect of “individual”. Latency to touch familiar food was log transformed to improve model residuals. Due to the large number of species represented (N=136), we selected bird order, rather than species (as listed in the pre-registration) for Objective 1, to facilitate capture of broad-scale differences and aid in interpretation of results. We used a linear mixed effects model (LMM) and analysed each condition (control or novel object tes[illegible]nd the individual difference between control and test conditions in each trial separately. LMMs were run using the *lme4* and *lmerTest* packages [98,99].

Objective 2 was to examine the effects of socio-ecological and other factors on neophobia across species. The final selection of nine factors was dependent on the species representation in the final sample and driven by our hypotheses (Table 1). The response variable was the “latency to touch familiar food”, the main effects were aforementioned factors, each in interaction with condition (control, novel object test). The random effects included were: “species”, with “individual ID” nested within “study site”, and “phylogeny” to control for non-phylogenetic and phylogenetic non-independence, respectively, among individuals. Models were run using Markov chain Monte Carlo (MCMC GLMM) estimation methods implemented in the MCMCGLMM package [100]. Model parameters included 1,000,000 iterations, 1,000 burn-in and a chain thinning of 50. We tested 1) the full dataset and 2) a reduced dataset of non-domesticated species only (removing the seven domesticated species), as we expected and found domestication status to have an effect on neophobia. Domestication was one of two additional factors (and body mass) included that were not socio-ecological variables, as domesticated species traits descend from their wild counterparts, thus domesticated species data may influence the other variables. Further, habitat variables for domesticated species were coded in line with their wild counterparts, as they no longer have truly natural habitats, so differed to coding of non-domesticated species in this aspect.

For both models, we generated a single phylogenetic tree from birdtree.org [60]; whilst more updated bird phylogenies have been presented (e.g. [101]), none of them has yet presented a species-level resolution that can be used with the species in our dataset, causing an issue for the analyses. Whilst

differences between newer bird trees and birdtree.org are small, the placement of some orders (most notably *Musophagiformes*, turacos) do differ; however, the number of potentially ambiguous species (N=3) was small, and our findings were robust to these differences in tree structure. The final data set had 13 species tested in the field and 123 species tested in captivity (lab, zoo, temporary captivity). With the limited number of species tested in the field, we did not include "site type" (field/captivity) in the analysis, though we did include "site" (i.e. institution/organisation name) ~~in the random effects~~. A simple "wild" versus "captive" category was also not possible to reliably code, as some 'captive' subjects were wild-caught, first or second generation wild-caught, or of unknown origin. Similarly, for field and some zoo sites, sex (N=544), age (N=322) and rearing history (N=694) were unknown or unreliable, therefore these factors were not included in the models. In sum, 65% of subjects touched the familiar food in the presence of a novel object in round 1 (75% in round 2). In round 1, 1439 subjects were tested; in round 2, 639 subjects were tested; in round 3, 326 subjects were tested.

Objective 3 was to test for repeatability of neophobia within subjects at the individual temporal (i.e. test round) and contextual (i.e. novel object test and control condition) levels. We extracted "R" (repeatability) estimates from models with individual as a random effect and ran a bootstrap (e.g. 1,000 samples) to generate 95% confidence intervals around the estimates (R package rptR [102], using rpt() function). For contextual repeatability, we included "condition" in the model and for temporal repeatability, we included "round", with the random effects as in Objectives 1 and 2 (as per [22]). We finally compared individual repeatability within each condition (i.e. control and test conditions separately).

We presented results of both 1) difference scores and 2) raw data and found consistent effects of dietary breadth and migration pattern across both approaches. From an open science perspective, we felt it was important to present both sets of findings for full transparency and highlight that the selection of different analyses, or the inclusion or removal of certain species, like removing domesticated species, can result in different outcomes. Habitat factors only affected neophobia in non-domesticated species and only using raw data.

Acknowledgements

Thank you to all site managers, where appropriate, for providing access to birds for data collection purposes. Please see S5 Table for a full list.

Additional Information

The authors declare no competing interests.

Author Contributions

Please see S6 Table for a full author contributions list. There were: 54 primary collaborators (i.e. someone who is "in charge" of a species/ site data collection and so takes the main responsibility for this data contribution to MB1, e.g. a lab leader and/or supervisor); 75 contributors (i.e. usually someone who is funded and/or directly supervised in their MB1 contribution by a primary collaborator) and 129 authors in total. Authorship was determined following the ManyBirds Project guidelines: https://themanybirds.com/authorship-guidelines-and-ethics-guidelines, which follow an adapted CRediT taxonomy (https://casrai.org/credit/).

Funding

Funding listed refers to funds obtained specifically for this study: Research Support Development Grant, Anglia Ruskin University to Rachael Miller. Other funding that directly supported contribution to this study through individually acquired awards is outlined in S6 Table.

Ethics Statement

It was the responsibility of each primary collaborator to ensure that the appropriate ethical approval was obtained prior to data collection in relation to this study. Furthermore, we had an overarching ethical approval for the wider study protocol under a University of Cambridge non-regulated procedure (NR2023/12) to Rachael Miller. Ethics approval for each site is outlined in S4 Table.

Data Availability

The full data set (including species with 600 second trial cut off), final analysis data set (removing species with 600 second trial cut off) and R coding script are available on: Figshare 10.6084/m9.figshare.27324972 (private link: https://figshare.com/s/046099ac8313ae194abf)

References

1. Beever EA, Hall LE, Varner J, Loosen AE, Dunham JB, Gahl MK, et al. Behavioral flexibility as a mechanism for coping with climate change. Frontiers in Ecology and the Environment. 2017;15(6): 299–308.

2. Coppens CM, de Boer SF, Koolhaas JM. Coping styles and behavioural flexibility: towards underlying mechanisms. Philosophical Transactions of the Royal Society B: Biological Sciences. 2010;365: 4021–4028.

3. Bond AB, Kamil AC, Balda RP. Serial reversal learning and the evolution of behavioral flexibility in three species of North American corvids (*Gymnorhinus cyanocephalus, Nucifraga columbiana, Aphelocoma californica*). Journal of Comparative Psychology. 2007;121(4): 372.

4. Amici F, Aureli F, Call J. Fission-fusion dynamics, behavioral flexibility, and inhibitory control in primates. Current Biology. 2008;18(18): 1415–1419.

5. Schaffer A, Caicoya AL, Colell M, Holland R, von Fersen L, Widdig A, et al. Neophobia in 10 ungulate species—a comparative approach. Behavioral Ecology and Sociobiology. 2021;75: 102.

6. Vardi R, Berger-Tal O. Environmental variability as a predictor of behavioral flexibility in urban environments. Behavioral Ecology. 2022;33(3): 573–581.

7. Dall SR, Houston AI, McNamara JM. The behavioural ecology of personality: consistent individual differences from an adaptive perspective. Ecology Letters. 2004;7(8): 734–739.

8. Sol D, Timmermans S, Lefebvre L. Behavioural flexibility and invasion success in birds. Animal Behaviour. 2002;63(3): 495–502.

9. Mazza V, Šlipogor V. Behavioral flexibility and novel environments: integrating current perspectives for future directions. Current Zoology. 2024;70(3): 304–309.

10. Kimball MG, Lattin CR. The “seven Deadly Sins” of neophobia experimental design. Integrative and Comparative Biology. 2023;64(1): 38-54.

11. Greenberg R, Mettke-Hofmann C. Ecological aspects of neophobia and neophilia in birds. Current Ornithology. 2001;16: 119–178.

12. Ferrari MC, McCormick MI, Meekan MG, Chivers DP. Background level of risk and the survival

of predator-naive prey: can neophobia compensate for predator naivety in juvenile coral reef fishes? Proceedings of the Royal Society B: Biological Sciences. 2015;282(1799): 20142197.

13. Dingemanse NJ, Both C, Drent PJ, Tinbergen JM. Fitness consequences of avian personalities in a fluctuating environment. Proceedings of the Royal Society of London. Series B: Biological Sciences. 2004;271(1541): 847–852.

14. Samuel L, Arnesen C, Zedrosser A, Rosell F. Fears from the past? The innate ability of dogs to detect predator scents. Animal Cognition. 2020;23: 721–729.

15. Candler S, Bernal XE. Differences in neophobia between cane toads from introduced and native populations. Behavioral Ecology. 2015;26(1): 97–104.

16. Cohen TM, Kumar RS, Nair M, Hauber ME, Dor R. Innovation and decreased neophobia drive invasion success in a widespread avian invader. Animal Behaviour. 2020;163: 61–72.

17. Mazza V, Czyperreck I, Eccard JA, Dammhahn M. Cross-context responses to novelty in rural and urban small mammals. Frontiers in Ecology and Evolution. 2021;9: 661971.

18. Krajcir KJ, Kelly TR, Kimball MG, Cochran EB, Stansberry KR, Dusang BA, et al. Eurasian tree sparrows are more food neophobic and habituate to novel objects more slowly than house sparrows. Biological Invasions. 2024;26: 3677–3693.

19. Coleman SL, Mellgren RL. Neophobia when feeding alone or in flocks in zebra finches, *Taeniopygia guttata*. Animal Behaviour. 1994;48(4): 903–907.

20. Mettke-Hofmann C, Winkler H, Leisler B. The significance of ecological factors for exploration and neophobia in parrots. Ethology. 2002;108(3): 249–272.

21. Walker MD, Mason G. Female C57BL/6 mice show consistent individual differences in spontaneous interaction with environmental enrichment that are predicted by neophobia. Behavioural Brain Research. 2011;224(1): 207–212.

22. Miller R, Lambert ML, Frohnwieser A, Brecht KF, Bugnyar T, Crampton I, et al. Socio-

ecological correlates of neophobia in corvids. Current Biology. 2022;32(1): 74–85.

23. Kluen E, Brommer JE. Context-specific repeatability of personality traits in a wild bird: a reaction-norm perspective. Behavioral Ecology. 2013;24(3): 650–658.

24. Vernouillet A, Kelly DM. Individual exploratory responses are not repeatable across time or context for four species of food-storing corvid. Scientific Reports. 2020;10: 394.

25. Kelly TR, Kimball MG, Stansberry KR, Lattin CR. No, you go first: phenotype and social context affect house sparrow neophobia. Biology Letters. 2020;16: 20200286.

26. Greggor AL, Jolles JW, Thornton A, Clayton NS. Seasonal changes in neophobia and its consistency in rooks: the effect of novelty type and dominance position. Animal Behaviour. 2016;121: 11–20.

27. Greenberg R. The role of neophobia in determining the degree of foraging specialization in some migrant warblers. The American Naturalist. 1983;122(4): 444–453.

28. Greenberg R. Neophobia, aversion to open space, and ecological plasticity in song and swamp sparrows. Canadian Journal of Zoology. 1989;67(5): 1194–1199.

29. Greenberg R. The role of neophobia and neophilia in the development of innovative behaviour of birds. In: Reader SM, Laland SM, editors. Animal Innovation. New York, NY, US: Oxford University Press; 2003. p. 175–96.

30. Mettke-Hofmann C, Winkler H, Hamel PB, Greenberg R. Migratory New World blackbirds (icterids) are more neophobic than closely related resident icterids. PLoS ONE. 2013;8(2): e57565.

31. Brown GE, Ferrari MC, Elvidge CK, Ramnarine I, Chivers DP. Phenotypically plastic neophobia: a response to variable predation risk. Proceedings of the Royal Society B: Biological Sciences. 2013;280(1756): 20122712.

32. Sabal MC, Boyce MS, Charpentier CL, Furey NB, Luhring TM, Martin HW, et al. Predation landscapes influence migratory prey ecology and evolution. Trends in Ecology and Evolution.

2021;36(8): 737–749.

33. Emery NJ, Clayton NS. The mentality of crows: convergent evolution of intelligence in corvids and apes. Science. 2004;306(5703): 1903–1907.

34. Beissinger SR, Donnay TJ, Walton R. Experimental analysis of diet specialization in the snail kite: the role of behavioral conservatism. Oecologia. 1994;100: 54–65.

35. Webster SJ, Lefebvre L. Neophobia by the Lesser-Antillean Bullfinch, a foraging generalist, and the Bananaquit, a nectar specialist. The Wilson Bulletin. 2000;112(3): 424–427.

36. Greenberg R. Feeding neophobia and ecological plasticity: a test of the hypothesis with captive sparrows. Animal Behaviour. 1990;39(2): 375–379.

37. Miller R, Bugnyar T, Pölzl K, Schwab C. Differences in exploration behaviour in common ravens and carrion crows during development and across social context. Behavioral Ecology and Sociobiology. 2015;69: 1209–1220.

38. Ducatez S, Sayol F, Sol D, Lefebvre L. Are urban vertebrates city specialists, artificial habitat exploiters, or environmental generalists? Integrative and Comparative Biology. 2018;58(5): 929–938.

39. Sol D, Griffin AS, Bartomeus I, Boyce H. Exploring or avoiding novel food resources? The novelty conflict in an invasive bird. PloS ONE. 2011;6(5): e19535.

40. Greggor AL, Clayton NS, Fulford AJ, Thornton A. Street smart: faster approach towards litter in urban areas by highly neophobic corvids and less fearful birds. Animal Behaviour. 2016;117: 123–133.

41. Klein RA, Ratliff KA, Vianello M, Adams RB, Bahník Š, Bernstein MJ, et al. Investigating variation in replicability: A “Many Labs” replication project. Social Psychology. 2014;45(3): 142-152.

42. Moshontz H, Campbell L, Ebersole CR, IJzerman H, Urry HL, Forscher PS, et al. The Psychological Science Accelerator: Advancing psychology through a distributed collaborative network. Advances in Methods and Practices in Psychological Science. 2018;1(4): 501–515.

43. Many Primates, Altschul DM, Beran MJ, Bohn M, Call J, DeTroy S, et al. Establishing an infrastructure for collaboration in primate cognition research. PLoS ONE 2019;14(10): e0223675.

44. Many Primates, Aguenounon G, Allritz M, Altschul D, Ballesta S, Beaud A, et al. The evolution of primate short-term memory. Animal Behavior and Cognition. 2022;9(4): 428–516.

45. Lambert M, Farrar B, Garcia-Pelegrin E, Reber S, Miller R. ManyBirds: A multi-site collaborative Open Science approach to avian cognition and behavior research. Animal Behavior and Cognition. 2022;9(1): 133–152.

46. ManyDogs Project MP, Alberghina D, Bray EE, Buchsbaum D, Byosiere S, Espinosa J, et al. ManyDogs Project: A big team science approach to investigating canine behavior and cognition. Comparative Cognition and Behavior Reviews. 2023;18: 59–77.

47. Alessandroni N, Altschul D, Baumgartner HA, Bazhydai M, Brosnan S, Byers-Heinlein K, et al. Challenges and promises of big team comparative cognition. PsyArXiv [Preprint]; 2024. doi:10.31234/osf.io/5nykg.

48. Coles NA, Hamlin JK, Sullivan LL, Parker TH, Altschul D. Build up big-team science. Nature. 2022;601: 505–507.

49. Krasheninnikova A, Chow PKY, von Bayern AM. Comparative cognition: Practical shortcomings and some potential ways forward. Canadian Journal of Experimental Psychology/Revue canadienne de psychologie expérimentale. 2020;74(3): 160-169.

50. Alessandroni N, Altschul D, Bazhydai M, Byers-Heinlein K, Elsherif M, Gjoneska B, et al. Comparative cognition needs big team science: How large-scale collaborations will unlock the future of the field. Comparative Cognition and Behavior Reviews. 2024;19: 67–72.

51. Takola E, Krause ET, Müller C, Schielzeth H. Novelty at second glance: a critical appraisal of the novel object paradigm based on meta-analysis. Animal Behaviour. 2021;180: 123–142.

52. Garcia-Pelegrin E, Clark F, Miller R. Increasing animal cognition research in zoos. Zoo Biology. 2022;41(4): 281–291.

53. Wilman H, Belmaker J, Simpson J, de la Rosa C, Rivadeneira MM, Jetz W. EltonTraits 1.0: Species-level foraging attributes of the world's birds and mammals. Ecology. 2014;95(7): 2027-2027.

54. Tobias JA, Sheard C, Seddon N, Meade A, Cotton AJ, Nakagawa S. Territoriality, social bonds, and the evolution of communal signaling in birds. Frontiers in Ecology and Evolution. 2016;4: 74.

55. Tobias JA, Sheard C, Pigot AL, Devenish AJ, Yang J, Sayol F, et al. AVONET: morphological, ecological and geographical data for all birds. Ecology Letters. 2022;25(3): 581–597.

56. Birdlife International. Data Zone [Internet]. BirdLife International; 2024 [cited 2024 Oct 15]. Available from: https://datazone.birdlife.org/species/spchabalt.

57. Neate-Clegg MH, Tonelli BA, Youngflesh C, Wu JX, Montgomery GA, Şekercioğlu ÇH, et al. Traits shaping urban tolerance in birds differ around the world. Current Biology. 2023;33(9): 1677–1688.

58. Sol D, González-Lagos C, Moreira D, Maspons J, Lapiedra O. Urbanisation tolerance and the loss of avian diversity. Ecology Letters. 2014;17(8): 942–950.

59. IUCN. The IUCN Red List of Threatened Species. V2022–2 [Internet]. IUCN; 2022 [cited 2024 Oct 15]. Available from: https://www.iucnredlist.org.

60. Jetz W, Thomas GH, Joy JB, Hartmann K, Mooers AO. The global diversity of birds in space and time. Nature. 2012;491: 444–448.

61. Revell LJ. phytools: an R package for phylogenetic comparative biology (and other things). Methods in Ecology and Evolution. 2012;3(2): 217–23.

62. Katzir G. Relationships between social structure and response to novelty in captive jackdaws, *Corvus monedula* L., I. Response to novel space. Behaviour. 1982;81(2-4): 231-63.

63. Réale D, Garant D, Humphries MM, Bergeron P, Careau V, Montiglio P. Personality and the emergence of the pace-of-life syndrome concept at the population level. Philosophical Transactions of the Royal Society B: Biological Sciences. 2010;365(1560): 4051–4063.

64. Dammhahn M, Dingemanse NJ, Niemelä PT, Réale D. Pace-of-life syndromes: a framework for the adaptive integration of behaviour, physiology and life history. Behavioral Ecology and Sociobiology. 2018;72(62): 1–8.

65. Sinclair AR, Mduma S, Brashares JS. Patterns of predation in a diverse predator–prey system. Nature. 2003;425: 288–290.

66. Zeder MA. The domestication of animals. Journal of Anthropological Research. 2012;68(2): 161–190.

67. Price EO. Animal Domestication and Behavior. Wallingford, UK: CAB International; 2002.

68. Suzuki K, Ikebuchi M, Kagawa H, Koike T, Okanoya K. Effects of domestication on neophobia: A comparison between the domesticated Bengalese finch (*Lonchura striata var. domestica*) and its wild ancestor, the white-rumped munia (*Lonchura striata*). Behavioural Processes. 2021;193: 104502.

69. Moretti L, Hentrup M, Kotrschal K, Range F. The influence of relationships on neophobia and exploration in wolves and dogs. Animal Behaviour. 2015;107: 159–173.

70. Bell AM, Hankison SJ, Laskowski KL. The repeatability of behaviour: a meta-analysis. Animal Behaviour. 2009;77(4): 771–783.

71. Greenberg R. Differences in feeding neophobia in the tropical migrant wood warblers *Dendroica castanea* and *D. pensylvanica*. Journal of Comparative Psychology. 1984;98(2): 131-136.

72. Dridi S, Lehmann L. Environmental complexity favors the evolution of learning. Behavioral Ecology. 2016;27(3): 842–850.

73. Sih A, Christensen B. Optimal diet theory: when does it work, and when and why does it fail? Animal Behaviour. 2001;61(2): 379–390.

74. Seferta A, Guay P, Marzinotto E, Lefebvre L. Learning differences between feral pigeons and zenaida doves: the role of neophobia and human proximity. Ethology. 2001;107(4): 281–293.

75. Mettke-Hofmann C, Ebert C, Schmidt T, Steiger S, Stieb S. Personality traits in resident and migratory warbler species. Behaviour. 2005;142(9/10): 1357–1375.

76. Griffin AS, Netto K, Peneaux C. Neophilia, innovation and learning in an urbanized world: a critical evaluation of mixed findings. Current Opinion in Behavioral Sciences. 2017;16: 15–22.

77. Pérez-Tris J, Díaz JA, Tellería JL. Loss of body mass under predation risk: cost of antipredatory behaviour or adaptive fit-for-escape? Animal Behaviour. 2004;67(3): 511-21.

78. Crane AL, Feyten LE, Preagola AA, Ferrari MC, Brown GE. Uncertainty about predation risk: a conceptual review. Biological Reviews. 2024;99(1): 238-52.

79. Greggor AL, Masuda B, Flanagan AM, Swaisgood RR. Age-related patterns of neophobia in an endangered island crow: implications for conservation and natural history. Animal Behaviour. 2020;160: 61–68.

80. Miller R, Laskowski KL, Schiestl M, Bugnyar T, Schwab C. Socially driven consistent behavioural differences during development in common ravens and carrion crows. PLoS ONE. 2016;11(2): e0148822.

81. Bebus SE, Jones BC, Elderbrock EK, Small TW, Schoech SJ. Neophobic behavior in free-living birds is highly repeatable and related to stress-induced corticosterone. In [Conference]: Annual Meeting of the Society for Integrative and Comparative Biology. 2015;55: E12-E12. Available from: https://sicb.org/abstracts/neophobic-behavior-in-free-living-birds-is-highly-repeatable-and-related-to-stress-induced-corticosterone/.

82. Lattin CR, Kelly TR, Kelly MW, Johnson KM. Constitutive gene expression differs in three brain regions important for cognition in neophobic and non-neophobic house sparrows (*Passer domesticus*). PLoS ONE. 2022;17(5): e0267180.

83. Webster MM, Rutz C. How STRANGE are your study animals? Nature. 2020;582: 337–340.

84. Crane AL, Ferrari MC. Patterns of predator neophobia: a meta-analytic review. Proceedings of

the Royal Society B: Biological Sciences. 2017;284: 20170583.

85. Bateson P, Laland KN. Tinbergen's four questions: an appreciation and an update. Trends in Ecology and Evolution. 2013;28(12): 712–718.

86. Krueger K, Farmer K, Heinze J. The effects of age, rank and neophobia on social learning in horses. Animal cognition. 2014;17: 645–655.

87. O'Hara M, Mioduszewska B, Von Bayern A, Auersperg A, Bugnyar T, Wilkinson A, et al. The temporal dependence of exploration on neotic style in birds. Scientific Reports. 2017;7: 4742.

88. Tobler M, Sandell MI. Yolk testosterone modulates persistence of neophobic responses in adult zebra finches, *Taeniopygia guttata*. Hormones and Behavior. 2007;52(5): 640–645.

89. Bertin A, Arnould C, Moussu C, Meurisse M, Constantin P, Leterrier C, et al. Artificially increased yolk hormone levels and neophobia in domestic chicks. Animals. 2015;5(4): 1220–1232.

90. Kelly TR, Lynch KI, Couvillion KE, Gallagher JN, Stansberry KR, Kimball MG, et al. A transient reduction in circulating corticosterone reduces object neophobia in male house sparrows. Hormones and Behavior. 2022;137: 105094.

91. Prasher S, Thompson MJ, Evans JC, El-Nachef M, Bonier F, Morand-Ferron J. Innovative consumers: ecological, behavioral, and physiological predictors of responses to novel food. Behavioral Ecology. 2019;30(5): 1216–1225.

92. Crates R, Stojanovic D, Heinsohn R. The phenotypic costs of captivity. Biological Reviews. 2023;98(2): 434–449.

93. Greggor AL, Clayton NS, Phalan B, Thornton A. Comparative cognition for conservationists. Trends in Ecology and Evolution. 2014;29(9): 489–495.

94. Miller R, Garcia-Pelegrin E, Danby E. Neophobia and innovation in Critically Endangered Bali myna, *Leucopsar rothschildi*. Royal Society Open Science. 2022;9(7): 211781.

95. Clements JF, Rasmussen PC, Schulenberg TS, Iliff MJ, Fredericks TA, Gerbracht JA, et al. The eBird/Clements checklist of Birds of the World: V2023 [Internet]. CornellLab Clements Checklist; 2023 [cited 2024 Oct 15]. Available from: https://www.birds.cornell.edu/clementschecklist/download/.

96. Friard O, Gamba M. BORIS: a free, versatile open-source event-logging software for video/audio coding and live observations. Methods in ecology and evolution. 2016;7(11): 1325–1330.

97. R Core Team. R: A Language and Environment for Statistical Computing. Vienna, Austria: R Foundation for Statistical Computing; 2023. Available from: https://www.R-project.org/.

98. Bates D, Mächler M, Bolker B, Walker S. Fitting linear mixed-effects models using lme4. Journal of Statistical Software [Internet]. 2015 [cited 2024 Oct. 26];67(1): 1-48. Available from: https://www.jstatsoft.org/index.php/jss/article/view/v067i01

99. Kuznetsova A, Brockhoff PB, Christensen RHB. lmerTest Package: Tests in linear mixed effects models. Journal of Statistical Software [Internet]. 2017 [cited 2024 Oct. 26];82(13):1-26. Available from: https://www.jstatsoft.org/index.php/jss/article/view/v082i13

100. Hadfield JD. MCMCglmm: Markov chain Monte Carlo methods for generalised linear mixed models. Tutorial for MCMCglmm package in R [Internet]. 2010 [cited 2024 Oct. 26];125. Available from https://citeseerx.ist.psu.edu/document?repid=rep1&type=pdf&doi=6e96c3449f03a8805b721398bad73fe51cd3b29c

101. Prum RO, Berv JS, Dornburg A, Field DJ, Townsend JP, Lemmon EM, et al. A comprehensive phylogeny of birds (Aves) using targeted next-generation DNA sequencing. Nature. 2015;526: 569–573.

102. Stoffel MA, Nakagawa S, Schielzeth H. rptR: Repeatability estimation and variance decomposition by generalized linear mixed-effects models. Methods in Ecology and Evolution. 2017;8(11): 1639–1644.

Supporting Information Captions

S1 Table: Socio-ecological factor coding

S2 Table: Inter-rater reliability

S3 Table: Generalised linear mixed models using Markov chain Monte Carlo techniques (MCMCglmm models), testing the effect of predictors on the difference (novel object minus control values) between control and test latencies to touch familiar food.

S4 Table: Site descriptions

S5 Table: Acknowledgements

S6 Table: Author contributions

S1 Fig. Mean latency to touch food in control (orange) and test (blue) conditions for each species, in relation to species average (log) body mass (g).

S2 Fig. Mean±SE latency to touch food in control (orange) and test (blue) conditions, in relation to social bond classification. Species sample sizes are given above the bars.

S3 Fig. Mean±SE latency to touch food in control (orange) and test (blue) conditions, in relation to habitat density in non-domesticated species only (raw data; seven domesticated lineages removed from the data set). Sample sizes are given above the bars.

S4 Fig. Mean±SE latency to touch food in control (orange) and test (blue) conditions in relation to habitat use, the number of habitat categories where a species was found (i.e. more habitat generalists vs specialists) in non-domesticated species only (raw data; seven domesticated species removed from the data set). Sample sizes are given above the bars.

Supporting Information

S1 Table: Socio-ecological factor coding

Full species list with socio-ecological and other factors coded: Figshare 10.6084/m9.figshare.27324972 (private link: https://figshare.com/s/046099ac8313ae194abf)

S2 Table: Inter-rater reliability

Inter-rater reliability: Figshare 10.6084/m9.figshare.27324972 (private link: https://figshare.com/s/046099ac8313ae194abf)

S3 Table: Generalised linear mixed models using Markov chain Monte Carlo techniques (MCMCglmm models), testing the effect of predictors on the latency to touch familiar food (i.e. raw data) on non-domesticated species only (seven domesticated species removed from data set).

	post.mean	L. CI	U. CI	Eff. samp	p_{MCMC}
(Intercept)	229.22	-351.12	841.60	19980	0.447
**Condition (test/control)**	503.11	316.66	691.11	19980	**<0.001**
Body Mass (log, grams)	-12.60	-60.07	36.81	19980	0.603
Dietary Breadth	6.03	-35.96	46.68	19980	0.774
Habitat Density	-1.27	-84.58	87.61	19411	0.976
Anthropogenic Habitat Use	13.05	-82.45	109.11	20535	0.790
Habitat Use	21.61	-10.83	55.68	19980	0.203
Social Bond (Short-term)	88.51	-15.95	192.20	19980	0.094
Social Bond (Solitary)	143.52	-613.88	897.07	19980	0.711
Territoriality (Seasonal)	-77.54	-237.80	82.61	19980	0.348
Territoriality (Year-Round)	-51.39	-244.06	129.02	19980	0.586
Migration (Nomadic)	247.79	-62.33	549.93	19980	0.114
Migration (Non-migrant)	-45.51	-155.26	64.69	19980	0.417
Condition x Body Mass	3.48	-8.95	16.57	19980	0.593
**Condition x Dietary Breadth**	-52.54	-71.62	-32.16	20794	**<0.001**
**Condition x Habitat Density**	59.60	20.80	98.80	20309	**0.002**
Condition x Anthropogenic Habitat Use	-51.83	-104.26	4.37	19980	0.064
**Condition x Habitat Use**	-21.77	-37.46	-6.81	19980	**0.005**
Condition x Social Bond (Short-term)	25.59	-34.18	81.99	19837	0.384
**Condition x Social Bond (Solitary)**	519.90	281.38	749.09	19980	**<0.001**
Condition x Territoriality (Seasonal)	47.88	-30.97	126.47	19980	0.234

Condition x Territoriality (Year-Round)	-16.91	-95.02	60.79	19980	0.672
Condition x Migration (Nomadic)	-89.54	-251.20	78.10	19980	0.287
**Condition x Migration (Non-migrant)**	-151.67	-216.61	-85.23	19980	**<0.001**

Individual ID was nested within the study site, and species and phylogeny were included in the model as random effects to control for non-phylogenetic and phylogenetic non-independence, respectively, among individuals. p_{MCMC} is twice the posterior probability that the estimate is negative or positive (whichever probability is smallest), L. CI = lower 95 credible interval, U. CI = upper 95 credible interval. Significant predictors are indicated in bold.

S4 Table: Site descriptions

Site descriptions including ethical approval per each site's primary collaborator: Figshare 10.6084/m9.figshare.27324972 (private link: https://figshare.com/s/046099ac8313ae194abf)

S5 Table: Acknowledgements

We thank the following organisations and people for facilitating this study by providing access to data collection sites or other contributions.

Name	Site	(Primary) Collaborator involved	Contribution
Ian Edmans	Waddesdon Manor, Rothschild Foundation, National Trust, UK	Rachael Miller	Access to birds
Polly Bramham	Birdworld, Farnham, UK	Rachael Miller	Access to birds
Helen Hitchman	Cotswold Wildlife Park & Gardens, UK	Rachael Miller	Access to birds
Erica Caching	Zoo Marine, Italy	James Davies	Facilitating testing with birds

Stefan Stadler	Zoo Frankfurt, Germany	Kai R. Caspar	Access to birds
Kerstin Ternes	Zoo Duisburg, Germany	Kai R. Caspar	Access to birds
Alexander Nolte	Zoo Duisburg, Germany	Kai R. Caspar	Access to birds
Antje Bauersfeld	Zoo Dortmund, Germany	Kai R. Caspar	Access to birds
Stephanie Zech	Zoo Dortmund, Germany	Kai R. Caspar	Access to birds
Sandra Dollhäupl	Zoo Duisburg	Kai R. Caspar	Access to birds
Bernd Marcordes	Zoo Köln, Germany	Kai R. Caspar	Access to birds
Alexander Sliwa	Zoo Köln, Germany	Kai R. Caspar	Access to birds
Arne Lawrenz	Zoo Wuppertal, Germany	Dominik Fischer	Access to birds
Karl Fischer	Raptor Center & Wildlife Zoo Hellenthal	Dominik Fischer	Access to birds
Wolfgang Kiessling	Loro Parque & Loro Parque Fundacion, Spain	Auguste von Bayern & Anastasia Krasheninnikova	Access to birds
Christoph Kiessling	Loro Parque & Loro Parque Fundacion, Spain	Auguste von Bayern & Anastasia Krasheninnikova	Access to birds
Jessica Borer	Zoo Basel, Switzerland	Vanessa Wilson	Access to birds
David Auz,	Charles Darwin Research Station	Paula Ibáñez de	Access to birds,

Courtney Pike, Sabine Tebbich, Irm Teschke, CDRS staff		Aldecoa	facilitating testing, resources
Marije van de Poll		Jorg Massen	Code videos for IRR test
Natalia Demergassi	Fundación Temaikèn	Jimena Lois-Milevicich	Access to birds & birds' info, facilitating testing
Andrés Suárez	Fundación Temaikèn	Jimena Lois-Milevicich	Access to birds & birds' info, facilitating testing
Zookeepers from CRET, ELA & "Patagonia" (Sector 4)	Fundación Temaikèn	Jimena Lois-Milevicich	Access to birds & birds' info, facilitating testing
Bettina Mahler	IEGEBA, CONICET	Jimena Lois-Milevicich	Resources (filming camera)
Štěpán Drda	University of South Bohemia	Petr Veselý	Facilitating testing
Margeaux Apple	Cambridge University Botanic Gardens	Julia Mackenzie	Facilitating site access
Grim Stjerndahl	Ystad Djurpark	Stephan A. Reber	Access to birds
The Pole Evans	Saunders Island, Falkland Islands	Katie Harrington	Facilitating site

Family			access, Resources
Nora Tabea Kopsch		Katie Harrington	Coded videos for IRR test
Daniel Issel		Kai R. Caspar	Coded videos for IRR test
Paul Emile Victor Institute	Kerguelen Islands (Verte Island)	Samara Danel	Facilitating site access and testing
Andrea Bračko	Zagreb Zoo	Elena Racevska	Access to birds
Innes Bliznac	Zagreb Zoo	Elena Racevska	Facilitating testing
Valentino Macan	Zagreb Zoo	Elena Racevska	Facilitating testing
Herbert Linnemann	Heimat-Tierpark Olderdissen, Bielefeld, Germany	Kai-Philipp Gladow	Access to birds
Markus Hinker	Heimat-Tierpark Olderdissen, Bielefeld, Germany	Kai-Philipp Gladow	Access to birds
Benjamin Aschmann	Adlerwarte Berlebeck, Detmold, Germany	Kai-Philipp Gladow & Kai R. Caspar	Access to birds
Stefan Schierhuber		Theresa Rössler & Alice Auersperg	Coded videos for IRR
Mariah Monsour	Pittsburg State University Natural History Reserve, Pittsburg, USA	Laurent Prétôt	Coded videos for IRR, Assisted with testing

S6 Table: Author contributions

Full list of authors, affiliations, funding and author contributions: Figshare 10.6084/m9.figshare.27324972 (private link: https://figshare.com/s/046099ac8313ae194abf)

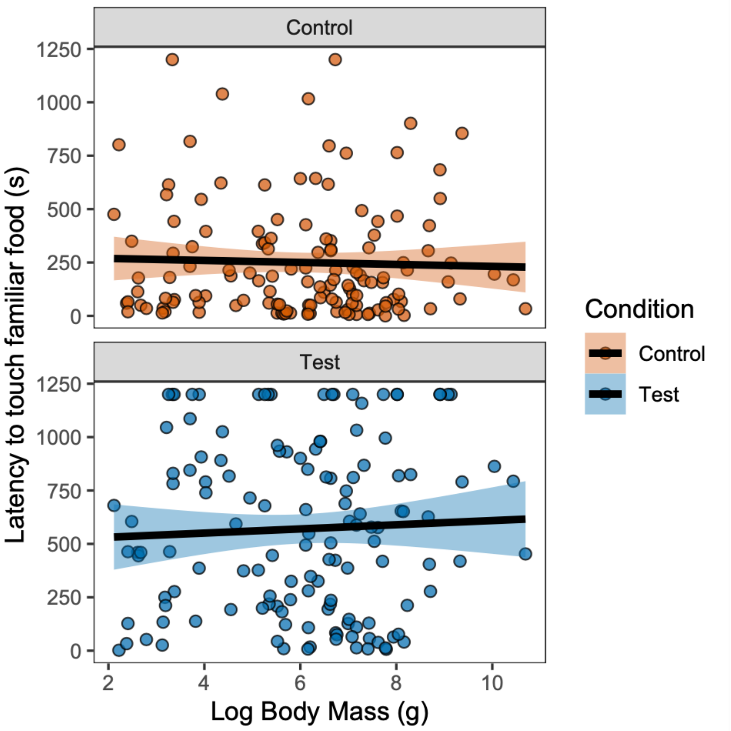

S1 Fig. Mean latency to touch food in control (orange) and test (blue) conditions for each species, in relation to species average (log) body mass (g).

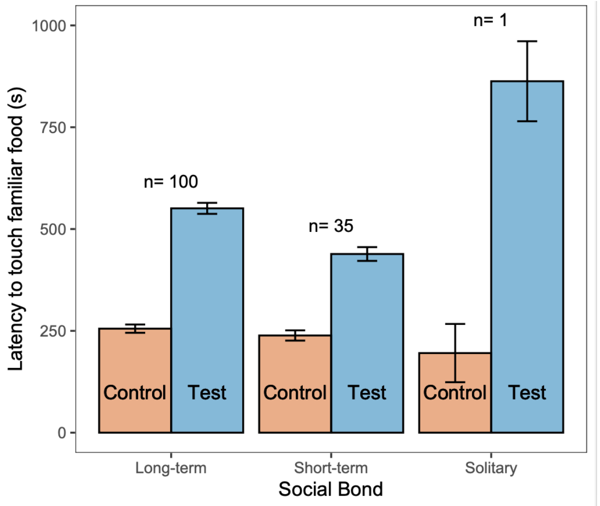

S2 Fig. Mean+SE latency to touch food in control (orange) and test (blue) conditions, in relation to social bond type. Species sample sizes are given above the bars.

We expected sociality to influence neophobia and found that, using raw data only, solitary species were

more neophobic than those with short- or long-term bonds. However, only one species (greater rhea, *Rhea americana*) was coded as having a “solitary” social bond (per [52]; i.e. pair bonds/ bi-parental care is non-existent/ restricted to courtship period), which may have driven this effect. We relied on peer-reviewed sources for consistency and to avoid potential coding bias. We followed [52] for social bond coding, however, we are concerned that greater rhea may not have been correctly classified as solitary in the original source, especially given that casuariform paleognaths were assigned a “short-term” status. Therefore, this finding should be treated with caution.

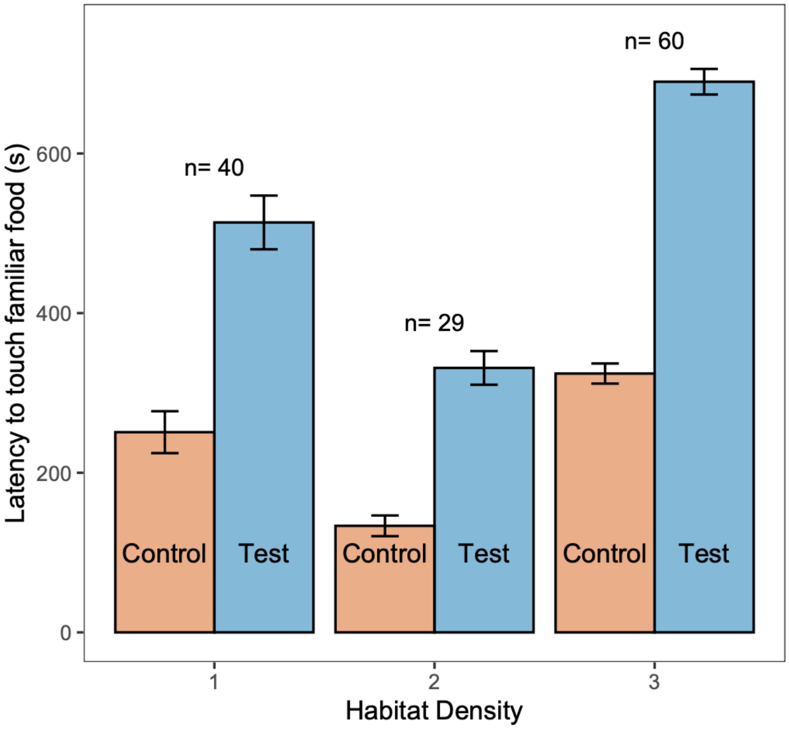

S3 Fig. Mean+SE latency to touch food in control (orange) and test (blue) conditions, in relation to habitat density (1 = high density; 3 = low density) in non-domesticated species only (raw data; seven domesticated lineages removed from the data set). Sample sizes are given above the bars.

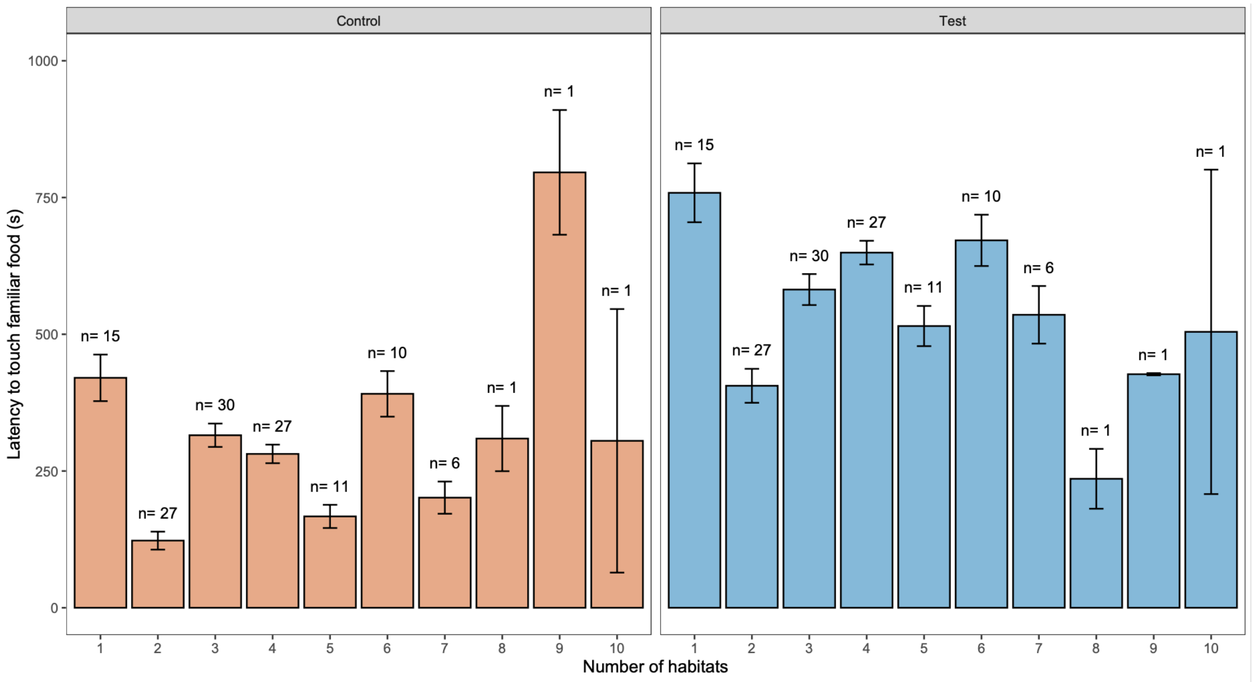

S4 Fig. Mean+SE latency to touch food in control (orange) and test (blue) conditions in relation to habitat use, the number of habitat categories where a species was found (i.e. more habitat generalists vs specialists) in non-domesticated species only (raw data; seven domesticated species removed from the data set). Sample sizes are given above the bars.
