# Supplementary material for: A large-scale study across the avian clade identifies ecological drivers of neophobia
Source: PLoS Biol. 2025 Oct 14;23(10):e3003394. doi: 10.1371/journal.pbio.3003394 (PMC12520376; doi:10.1371/journal.pbio.3003394)
Supplement: Attachment — Submitted filename: Response to Reviews.docx [file pbio.3003394.s004.docx]

**ManyBirds Study 1: PLOS BIOLOGY Response to Editor and Reviewers**

Thank you for your patience while your manuscript "Evolutionary drivers of neophobia across the avian clade" was peer-reviewed at PLOS Biology. Your manuscript has been evaluated by the PLOS Biology editors, an Academic Editor with relevant expertise, and by three independent reviewers.

As you will see in the reviewer reports, which can be found at the end of this email, although the reviewers find the work potentially interesting, they have also raised a substantial number of important concerns. Based on their specific comments and following discussion with the Academic Editor, it is clear that a substantial amount of work would be required to meet the criteria for publication in PLOS Biology. However, given our and the reviewers' interest in your study, we would be open to inviting a comprehensive revision of the study that thoroughly addresses all the reviewers' comments. Given the extent of revision that would be needed, we cannot make a decision about publication until we have seen the revised manuscript and your response to the reviewers' comments. Your revised manuscript would need to be seen by the reviewers again, but please note that we would not engage them unless their main concerns have been addressed.

You'll see that reviewer #1 says “the study overcomes an important short-coming in research addressing questions of this kind, namely, species specificity,” but notes a number of significant issues. The first is that the sheer breadth means that it’s much less focussed. S/he also thinks that the lack of clear hypotheses runs the risk of p-hacking and multiple testing problems, that the paper may be better presented as multiple studies (please do not do this - see my note below), that the fact that 90% of the birds are in captivity deserves significant comment, that the degree of consistency between species is unclear, and there is no surprising take-home. Reviewer #2 is more positive, saying, “The scope and ambition of this work are commendable, and the contribution has significant potential.” S/he suggests removing the first part of the Intro, spelling out the research goals more explicitly (including more alternative hypotheses), including better connection between the

results and the hypotheses. S/he also suggests changes to the analyses – include control latencies as a covariate, perform log-transformations, capitalise more on the potential of your Bayesian framework, and better clarify/justify your analytical choices. S/he also attached a marked up PDF. Reviewer #3 raises several concerns, some of which that overlap with the other reviewers, and says that in order to address your stated research questions, you need to better account for phylogeny and a range of life history trait variables. S/he also questions the connection between results and stated hypotheses (see reviewer #2).

Having discussed the reviews with the Academic Editor, we think you should try to make the framing of the paper more coherent, including clear up-front hypotheses (R2). There are also a number of specific requests for additional analyses and clearer defence of current analytical choices (R2). The captive nature of most of the subjects should be clearly flagged as a limitation (R1). IMPORTANT: After discussion with the Academic Editor, we encourage you NOT to split up the manuscript, as one of the reviewers suggests ("A split is unnecessary and would diminish the impact of the results. I think the results are interesting enough to justify allowing the authors to revise and respond to the comments").

We appreciate that these requests represent a great deal of extra work, and we are willing to relax our standard revision time to allow you 6 months to revise your study. Please email us (plosbiology@plos.org) if you have any questions or concerns, or envision needing a (short) extension. At this stage, your manuscript remains formally under active consideration at our journal; please notify us by email if you do not intend to submit a revision so that we may withdraw it.

Sincerely,

Roli Roberts

Roland Roberts, PhD

Senior Editor

PLOS Biology

[rroberts@plos.org](mailto:rroberts@plos.org)

**RESPONSE TO EDITORS:**

**Thank you very much for the feedback and comments. We have now made major changes to the paper following the Editor and Reviewer comments, addressed below and in the manuscript (submitted as a clean version and a tracked changes version, with red text to signify changes). Please note line numbers below correspond with the tracked changes manuscript version.**

**These changes included: clearer up-front hypotheses (e.g. new column in Table 1; expanded text in Introduction) and links to results (expanded text in Discussion; clearer links with Neophobia Threshold Hypothesis; Dangerous Niche Hypothesis; Life History Theory), new analyses (e.g. use of Bayesian models for all appropriate analyses; inclusion of new factors “social context” and “trial order”; repeatability analyses done across species, family and order) and clearer justification of existing analytical choices (e.g. moving taxonomic order analysis to Supplementary Materials; including model logistics information) and extensive Discussion of the captive nature of most subjects. We expanded the Discussion to highlight the ‘take-home’ messages and re-organised the research objectives as requested.**

**As recommended by the Editors, we did not split the paper. In line with this, to avoid further extending/ complicating this already extensive paper and as our primary goal was to explore ecological drivers on neophobia, we also did not include further life history traits in our analyses, though we include this decision more clearly as a future direction in Discussion points. In relation to this, we have changed our title to “Ecological drivers…” though we are very open to Editor thoughts on this too (title was previously “Evolutionary drivers…”).**

-----------------------------------------

**REVIEWER' COMMENTS:**

**Reviewer #1:**

This paper is the product of a large-scale study, on 136 bird species by 129 collaborators - the ManyBirdsProject. The coordination of such a study to the point of manuscript submission is extremely impressive and the authors deserve congratulations for pulling it off. The research question being addressed is - what are the evolutionary drivers of neophobia? - and the hypotheses being tested range from phylogenetic to social behaviour, migratory behaviour, ecology and diet. Each species in the study was tested for neophobic tendencies with the same experiment to allow for comparison across species.

Generally speaking, the study overcomes an important short-coming in research addressing questions of this kind, namely, species specificity. The authors note that when there have been attempts to compare neophobic behaviours across species, comparisons have been restricted to a group of species within the same family, for example, crows. While there are comparative methods for comparing data from across species - meta-analysis, for example - the authors here have gone one step further and organise efforts to replicate the same experiment; the theory being that this will provide directly comparable data. So, along with the effort, the aims are laudable, and the study has identified some clear patterns in the data.

The trade-off is that the bigger your study, the less focussed it is, the greater the risk of missing important details, eg species differences; the more you need to think about the risks of multiple comparisons (the need for Bonferroni correction for example).

**Thank you for your comments; please see our responses below. The changes were made in the revised manuscript, in the line numbers mentioned below. Please note line numbers correspond with the ‘tracked changes’ manuscript version.**

I've listed some points below that the authors may wish to consider or clarify:

-	I wasn't clear to me whether hypotheses were tested a priori, and if so, what those were. This is so important in a study like this because of the risk of p-hacking accusation and failing to account for multiple comparisons.

**Yes, we did have a-priori hypotheses to test, and we have now expressed these more clearly in the manuscript (particularly in the final four paragraphs of the Introduction outlining the research goals/ objectives; lines 275-318) and explicitly in Table 1 (new column - Neophobia Threshold Hypothesis; Dangerous Niche Hypothesis; Life History Theory).**

-	There may just be too much in this for one paper: any one of ecology, sociality, phylogeny, diet could be publishable alone - putting them all together is a little overwhelming and precludes in-depth examination of the data.

**We agree there is a tension between depth and breadth, particularly taking into account the word count limits of the journal. In this case the Editor asked us not to split the paper into two. We note that this request also supports not expanding this particular paper to include broader life history factors (Reviewer 3 suggestion), which could alternatively be a focus of a second/ future paper - a point we have now added to the Discussion (lines 636-645).**

-	I was astonished that 90% of the subjects were captive birds -this seems extremely high and I wanted to understand the implications of this a little more. To what extent is captivity expected to impact on neophobia and are some species likely to be impacted more than others: raptors vs budgies?

**We have now expanded this section of the Discussion substantially, including a discussion of why we might see this bias in our sample and the extent to which captivity in general may impact neophobia responses (lines 570-599). Following this comment and that of the Editor we conclude by explicitly acknowledging this limitation and encouraging future work to better understand its potential impact. We, however, also note in the Discussion that captive testing could be viewed as a strength of our study, given it is typically more controlled than field testing.**

**“For instance, although we were open to including neophobia data from wild animals, 90% of our subjects were sampled in lab and zoo captive settings rather than field settings, potentially reflecting logistic constraints in some cases (e.g. difficulties in identifying unique individuals in the field, obtaining permits for fieldwork, higher logistic costs, etc.). The large percentage of captive birds sampled can be viewed as a strength of the study, given that captive settings are more controlled and more easily standardised. While it is possible that individual differences in neophobic responses are less pronounced in captivity, neophobia has been shown in songbirds to be consistent between natural and captive settings [91].**

**Our study sample reflects a general over-representation of captive sites in avian cognition research but diverges from previous work by including a large number of zoo-housed subjects (see [41]). It may indicate a form of ‘self-selection’ by contributors, such that those with access to captive animals were more willing or able to collect the relevant data within the specified time frame, for example, through short-term student projects. We controlled for this variance wherever possible in the analyses, by including factors like domestication status and by analysing difference scores as well as raw data. We also provided as many details as possible for each study site (S7 Table), allowing for possible future investigations into the effects of site-specific factors not considered in the present analyses.**

**Nonetheless, in the present study, it was not possible to directly test the effects of captivity on object neophobia due to the relatively small sample of wild species tested (which were also taxonomically skewed). Available evidence suggests that captive bird populations may differ drastically from wild ones regarding various aspects of behaviour and cognition [92], placing an important caveat on studies that, like ours, rely heavily on captive-bred subjects from zoos and laboratories. There is also data suggesting marked differences between species regarding chronic stress in captive settings, which might be pronounced (even if the individuals in question have been raised in and used to captivity) and can be influenced by inconspicuous differences in housing conditions [93-95]. Our results should therefore be considered with these limitations in mind, and we encourage more comparative work explicitly testing effects of captivity on neophobia.”**

-	The experimental set-up, picture in Figure 7 made me wonder if the same thing really was tested across 136 species - is it a risk that some species will react to these objects differently from others in a way that obscures underlying neophobia, frugivores may be more comfortable with bright colours for example.

**We have now addressed the concern about the potential variability in species' responses to the novel object stimuli due to differences in ecological or perceptual factors (e.g., frugivores and colour preference) in the Discussion (lines 600-609)**

**“Another potential limitation of our study relates to the standardisation of the novel object stimuli across the broad taxonomic range of species tested. While every effort was made to ensure consistency in object construction and presentation (e.g. by using a standardised colour palette, shiny texture in part of the object, and scaled size), species may nonetheless differ in how they perceive or interpret specific object features due to species differences in sensory physiology or ecology. For example, frugivorous species might be more accustomed to or attracted by brightly coloured objects, potentially resulting in lower observed neophobia that may not reflect broader novelty responses [96]. While such perceptual or ecological differences are difficult to eliminate entirely in large comparative datasets, future studies should build on this work by testing responses to different object types that vary in specific features (e.g. colour, texture, shape) to further disentangle perceptual biases from general neophobic tendencies.”**

-	I needed some help from the authors to assess the significance of the findings. None of them seemed that surprising to me: neophobia has a phylogenetic signature (few traits don't); generalists are less neophobe than specialists.. did we need this study to tell us that? I was missing a sense of "now we know!" which you might expect from a study on this scale.

**We have now expanded the Conclusion section to assess the significance of the findings - please see below and lines 651-669.**

**“Our understanding of the evolutionary drivers of neophobia has been limited by a lack of taxonomically diverse comparative studies. Drawing on a sample of 136 bird species, we examined the repeatability of object neophobia at multiple taxonomic levels and then tested predictions regarding eight different ecological variables in relation to interspecific neophobic variance. Across four models drawing on both raw data and difference scores as well as data subset for non-domesticated species, our results revealed robust support for predictions of the Neophobia Threshold Hypothesis and Dangerous Niche Hypothesis, in that both dietary breadth and migratory habits were predictors of neophobia. We also found some evidence for additional effects of territoriality, habitat density and diversity, and domestication impacting neophobic responses.** **Why, then, do dietary breadth and migratory habits stand out as robust predictors of neophobic behaviour? Because these two ecological factors are supported by two distinct hypotheses (the Dangerous Niche Hypothesis for migration, and the Neophobia Threshold Hypothesis for feeding), different selection pressures are likely to affect each. Our results suggest that neophobia is under selection pressure in a migration context because of risk mitigation but is less important in other contexts such as navigating social situations. For feeding behaviour, neophobia may act more as a constraint, restricting the evolution of a generalist feeding strategy, and this may be stronger for feeding than for traits such as territoriality or habitat complexity. Our findings make clear advances in our understanding of the ecological drivers of neophobia, while providing support for two of the central hypotheses explaining the evolution of this behaviour. This study therefore lays a strong foundation for future neophobia studies to build from.”**

**Reviewer #2:**

NOTE: ALSO ATTACHED ANNOTATED PDF

This manuscript represents an impressive effort to compile high-resolution data on an important behavioral feature—neophobia—across a wide range of species, aiming to identify the socio-ecological drivers of evolutionary variation. The scope and ambition of this work are commendable, and the contribution has significant potential to advance our understanding of the adaptive nature of neophobic responses.

However, there are several aspects where the manuscript could be improved to strengthen its clarity, logical flow, interpretation of results, and overall impact. Below, I outline specific suggestions for improvement, organized into conceptual issues and methodological suggestions:

**Thank you for your comments, please see our responses below and in the lines of the revised manuscript. Please note line numbers correspond with the ‘tracked changes’ manuscript version.**

1. Conceptual Issues

First, the initial focus on behavioral flexibility in the introduction seems unclear and unnecessary, given that this link is not revisited later in the discussion. The central argument of the manuscript is that neophobia is stable enough for meaningful comparisons across species. Therefore, I suggest removing the first section of the introduction discussing flexibility and instead beginning directly with a definition of neophobia and its ecological and evolutionary significance. This would sharpen the manuscript's focus.

**We have addressed this comment by removing the focus on behavioural flexibility in the first paragraph and introducing neophobia immediately here instead (see lines 159-167).**

**Introduction: “Aversion to novelty, also known as neophobia, is often measured as the latency (i.e. time taken) to approach or interact with novel stimuli (e.g. objects or food) compared to familiar stimuli [1]. Neophobic responses can protect an individual from potential risks, but may also decrease opportunities to exploit novel resources, such as unknown food or nesting sites [2]. Thus, patterns of neophobic responses across taxa are likely to be driven by both costs and benefits [3], and these are linked to taxon-specific ecological and social factors [4]. Neophobia impacts adaptability and survival [5,5], and can affect ecological processes [7], including invasiveness (cane toads, *Rhinella marina* [8]; common mynas, *Acridotheres tristis* [9]), and range and niche expansion (small mammals [10]; Eurasian tree sparrows, *Passer montanus* [11]).”**

The research goals should be reorganized for better logical structure. The first step is to test for individual differences in neophobia, as this measures repeatability and directly assesses plasticity. This approach demonstrates that, despite substantial plasticity, there is a stable component within individuals. Note however that even if no stable component were found, species-level differences in neophobia would not be negated. The second step is to actually assess differences across species. This evaluates whether species differ significantly in neophobia despite plasticity (i.e. neophobia is a species feature), a major assumption of the comparative approach. Including phylogeny as a random effect can further help assess to what extent differences across species arise from shared ancestry (i.e. ravens are neophobic because they are corvids and hence share a common ancestor and similarities in ecological lifestyle). Finally, the third step is to evaluate whether these taxonomic differences are driven by socio-ecological factors. This is the most significant contribution of the manuscript but should logically follow the validation of interspecific differences in neophobia.

**We have re-ordered the research objectives as the reviewer suggested (lines 248-252), though in response to yours and other reviewer comments, we now combine objectives 2 (taxonomic order differences) and 3 (ecological drivers) and have moved all results/ analysis for the old objective 2 to the Supplementary Materials.**

**“We had two main objectives. The first was to examine whether neophobia was entirely plastic with no evidence of evolutionary divergence at any level or whether consistent differences in neophobia existed among individuals, among species and among other taxonomic levels in our dataset. We then examined what ecological factors-including diet, sociality, habitat and range-predicted neophobia, while accounting for phylogeny (Table 1).”**

The theoretical framework could be expanded to include additional hypotheses. Beyond the Neophobia Threshold Hypothesis (NTH) and Dangerous Niche Hypothesis (DNH), the manuscript could incorporate life history theory, which predicts that species with slow life histories (e.g., long reproductive lifespans) are risk-averse, while those with fast life histories (e.g., high fecundity) are likely risk-takers. This framework could explain substantial variation in neophobia and help interpret related results, such as the relationships between body size, innovativeness, invasiveness, and migration. Relevant work, such as studies on fear responses to humans, could be referenced (e.g., [https://doi.org/10.1007/s00265-018-2463-0)](https://eur03.safelinks.protection.outlook.com/?url=https%3A%2F%2Fdoi.org%2F10.1007%2Fs00265-018-2463-0)&data=05%7C02%7Crmam3%40universityofcambridgecloud.onmicrosoft.com%7C7bd0915c14024f5bdb6408dd54e6ed3a%7C49a50445bdfa4b79ade3547b4f3986e9%7C1%7C0%7C638760073519424876%7CUnknown%7CTWFpbGZsb3d8eyJFbXB0eU1hcGkiOnRydWUsIlYiOiIwLjAuMDAwMCIsIlAiOiJXaW4zMiIsIkFOIjoiTWFpbCIsIldUIjoyfQ%3D%3D%7C0%7C%7C%7C&sdata=JgXkNXOIcj%2FX1cRsrqaCUwnzwOvR9uVkFt01xm6x9YY%3D&reserved=0).

**Great suggestion. We agree with this Reviewer and have expanded on Life History Theory in the Introduction and Discussion (lines 186-192, 303-315 and lines 636-645)**

**Introduction: “Neophobia should also be shaped by life history traits. Life History Theory predicts that species with slow life histories (e.g. long-life expectancy, long reproductive lifespans, slower growth) would be more risk-averse and thus neophobic, while species with fast life histories (e.g. short life expectancy, high fecundity, faster growth)** **would be likely risk-takers [26,27]. This framework may help to explain associations between neophobia and species traits such as body size and domestication status [28,29], although a more thorough test of the predictions of Life History Theory would require comparing neophobia in a wider range of taxa than has been done thus far.”**

**Introduction: “Body size has been linked to Life History Theory, and we thus expect larger birds to have a slower pace of life and to be more neophobic [60,61]. However, larger body size has also been linked to lower predation pressure, which is expected to result in lower neophobia in larger species, as per the Dangerous Niche Hypothesis [62]. In this case, the two hypotheses about the relationship between body size and neophobia are mutually exclusive, although it is also possible that different ecological drivers may shape neophobic responses in different avian families. With domestication, lower reactivity and reduced fear or stress responses are often considered desirable traits among domestic animals, allowing them to cope and reproduce more effectively in human-controlled environments [63-66]. Domesticated animals are also bred to have more offspring and shorter generation times, and the anthropogenically modified environments they dwell in are safer than wild environments in terms of predation and food availability [67]. Therefore, both the Dangerous Niche Hypothesis and Life History Theory would predict that levels of neophobia should be lower in domesticated lineages compared to non-domesticated ones.”**

**Discussion: “Body mass and domestication status were the only life history-related predictors coded and tested with our full dataset, with no effect found for body mass and domesticated birds showing lower neophobia, as predicted by Life History Theory and previous work on domesticated birds [29]. Although additional variables (e.g. clutch size) could have been added to this analysis, there was a trade-off between maximizing the value of this particular dataset and the risk of finding false positives from running many analyses, especially considering that we were already examining raw data and differences score data for each variable, both with and without domesticated species. Future studies on object neophobia should focus more on the influence of life history factors such as longevity, developmental speed and reproductive strategy (r/K strategies). Furthermore, potential effects of neurological variables may be explored, which we did not consider here due to the ecological focus of this study.”**

Another missing hypothesis is the null hypothesis that neophobic responses are highly plastic, with no consistent differences across species. This would explicitly test whether species differences reflect inherent traits or environmental flexibility. On a side note, as the NTH and DNH are heavily based on Russell Greenberg's work, adding his name before the citation could be a meaningful homage.

**We have now added the null hypothesis in the Abstract and Introduction as follows (see lines 148-151, 277-281):**

**Abstract: “We first demonstrated that consistent differences in neophobia existed among individuals, among species and among other taxonomic levels in our dataset, rejecting the null hypothesis that neophobia is highly plastic at all taxonomic levels with no evidence for evolutionary divergence.”**

**Introduction: “We hypothesised that neophobic responses are consistent across individuals and species; alternatively, responses may be highly plastic, with no consistent differences across individuals, species, or even higher taxonomic groups. We thus tested this hypothesis, noting that species may differ in baseline neophobia despite individual plasticity (i.e. neophobia is a species feature).”**

**We also mention Greenberg by name in the Introduction as follows (lines 175-177): “Although the drivers of variation in neophobia are relatively unknown, there are at least two non-mutually exclusive hypotheses specifically addressing between-species variation in neophobia, which are inspired by Russell Greenberg’s seminal studies [19-21]; see also [22]”.**

The logic of the specific hypotheses tested in the study needs clearer explanation. While Table 1 outlines these hypotheses, it is not immediately clear what predictions they make or how they relate to the broader theoretical frameworks offered by the NTH and DNH. For example, it is unclear why body size or territorial behavior should influence neophobia. Some of these connections only become clear in the discussion, while others remain unresolved. Including these predictions directly in Table 1 would help clarify their rationale and strengthen the manuscript's presentation.

**We have now included the predictions related to NTH and DNH in Table 1 for all hypotheses.**

The manuscript needs stronger connections of the specific hypotheses of Table 1 and the NTH and DNH. Without this, it is unclear how the results support these theoretical frameworks. After reading the manuscript, I'm still unsure to what extent the specific hypotheses allow to disentangle between the NTH and DNH. For example, being an island dweller may reduce neophobia, as shown for parrots by Mettke-Hoffman. This is consistent with both the NTH and DNH, as islands harbour fewer predators and provide ecological opportunities should reduce neophobia. The authors do not test this particular hypothesis, perhaps due to the lack of data, but the issue I illustrate may apply to many of the specific hypotheses presented in Table

**We have expanded Table 1 to include predictions including showing relation to NTH and DNH. We also ensure results are more clearly tied to each hypothesis/ prediction in the Discussion for example (lines 457-459): “We found evidence in support of the Neophobia Threshold Hypothesis, including effects of dietary breadth and habitat use diversity [19,23], the Dangerous Niche Hypothesis, including an effect of migratory pattern [24], and Life History Theory, including an effect of domestication [29].”**

2. Methodological Suggestions

Regarding the response variables, the authors currently use two approaches: the difference between treatment and control conditions, and the raw latency data for each condition. However, variation in the latency to touch the food within and across species can be greatly influenced by motivation to feed. For example, species with higher metabolic rates need to forage more frequently, which may be reflected in shorter latencies for both the treatment and control.

**This is partly accounted for by the body mass factor (e.g. smaller birds may need to feed more often), an argument which we have now added into the Discussion (lines 537-540): “Body mass may also serve as a potential control for motivation as smaller species with higher metabolic rates may need to forage more frequently, and thus have shorter latencies for control and novel object test trials. However, we did not find any effect of body mass on neophobia.”.**

**In addition, the majority of subjects are captive and therefore fit within a regimen of regular feeding times and are therefore likely to be motivated to feed. It was not possible to directly correct for “motivation” as this is an individual transient state that we could not quantify or control. Our approach, with large sample sizes across species, still allows us to test higher order effects, whilst some unexplained variation in the model will remain from unknown variables such as “motivation”.**

This makes some results with raw data difficult to interpret. A better approach would be to include the control latencies as a covariate in the model.

**We respectfully disagree - we do conduct all analyses with both raw and difference score data, and we need to see how variables differ between the control and test conditions. However, adding control latencies would not allow this, but rather mirror the difference score analysis. Furthermore, it would make interpretation harder than it is. Additionally, Bayesian models do not produce residuals.**

**Please also see expanded justification for inclusion of raw and difference score data added to last paragraph of Introduction (lines 244-248): “Difference scores provide the change between the two conditions and reflect the actual neophobia elicited, and are independent of the control measure, i.e., how much longer an individual waited to feed with the novel object present. However, where this difference occurs (e.g. at fast or slow latencies) and its context is functionally meaningful, which is captured by the raw data”**

**And Methods (lines 907-914): “The difference scores are independent of the control latency and comparable between individuals and species. For example, two birds (A, B) may have a similar difference score (e.g. low neophobia), though bird B took longer to approach the familiar food than bird A (longer control latency), reflecting an individual/ species difference in general approach to food (which may be influenced by e.g. hunger, motivation, competition). It does not (necessarily) mean bird B is more neophobic than bird A, therefore both raw and difference score data is informative for the magnitude of difference as well as where this occurs. For example, with the dietary breadth result, both control and test latencies decline at greater breadth, but the declines are greater under test conditions.”**

Additionally, latency data often deviate from normal distributions, even after log-transformation, and care should be taken to select an appropriate distribution to model it (the BRMS package in R offers some distributions that can be useful here).

**In line with standard practice (see Midway and White 2025 for a recent discussion), we focused on whether diagnostics and model convergence were appropriate in our MCMCglmm.**

**We confirm that model diagnostics were fine - we added the following information to the Methods lines 899-905: “For both models (raw and difference score data), to ensure robust convergence diagnostics, we ran three separate chains of the model with different seeds. We assessed the convergence of the MCMC chains using the Gelman-Rubin diagnostic from the coda package [113]. The chains were combined into an mcmc.list object and the Gelman-Rubin diagnostic was calculated, which indicated good convergence for all parameters in the model. For both models, the potential scale reduction factors (PSRF) were all close to 1, with upper confidence intervals also near to 1, suggesting that the chains have likely converged well, providing reliable estimates for the model parameters.”**

**Midway S, White JW. Testing for normality in regression models: mistakes abound (but may not matter). Royal Society Open Science. 2025 Apr 30;12(4):241904**

On the other hand, differences between treatment and control should be log-transformed to make them proportional and less affected by latency magnitude.

**Thank you for this suggestion - in the case of our data, applying this transformation was not appropriate because the data set included negative values. As the model diagnostics are good - we have added information on this to the methods/ statistical analysis section (see below and lines 899-905), we do not see any need for a transformation. Furthermore, in a comparable study in corvids, Miller et al (2022) did not log transform neophobia latency data for analyses.**

**“For both models (raw and difference score data), to ensure robust convergence diagnostics, we ran three separate chains of the model with different seeds. We assessed the convergence of the MCMC chains using the Gelman-Rubin diagnostic from the coda package [113]. The chains were combined into an mcmc.list object and the Gelman-Rubin diagnostic was calculated, which indicated good convergence for all parameters in the model. For both models, the potential scale reduction factors (PSRF) were all close to 1, with upper confidence intervals also near to 1, suggesting that the chains have likely converged well, providing reliable estimates for the model parameters.”**

Furthermore, the order of treatment and control presentation should be included as a fixed effect in the model (see justification below).

**Thank you for this suggestion. We have included this as a new factor “test order” in our models as: control-test, test-control and (in very few cases) both control/test presented on the same day.**

The Bayesian framework employed in the manuscript, specifically the use of MCMCglmm, is a strong methodological choice but has not been fully utilized. Analyzing neophobia at the individual level allows for the estimation of intraclass coefficients, measuring variance across individuals, species, and phylogenetic levels simultaneously. This approach could include covariates such as sex and age, avoid reliance on taxonomic levels to assess phylogenetic effects, and provide direct measures of phylogenetic heritability. It would also help distinguish between variance explained by phylogeny versus species-specific idiosyncrasies, further refining the analysis.

**Thank you for this suggestion. We have now included variance components for individuals, species and phylogeny, but cannot include age, sex and other such covariates as we do not have all of this information for each individual or indeed species (e.g. field sites). Moreover, the inclusion of multiple levels of random effect at Order or Family, starts to obscure the aims of the model. We have, however, made changes according to the suggestions about repeatability made by Reviewer 3. These changes are as follows: we have now calculated repeatability at individual, species, family and order level (see Individual repeatability section in Results and new Supplementary Table), which we think adds another useful perspective.**

The current approach of adding all predictors into a single model also risks obscuring relationships due to collinearity, confounding effects, or varying measurement errors. For example, testing the effects of urban environments and generalist foraging behavior in the same model may yield misleading results if there is a causal relationship between them. If generalist foraging predicts urban success, which in turn influences neophobia, modeling them simultaneously may mask these causal pathways. A better approach would involve constructing directed acyclic graphs (DAGs) to represent the causal relationships among predictors, followed by testing these relationships using phylogenetic path analysis.

**This is an interesting approach and one that we considered. Unfortunately, current phylogenetic path analysis frameworks are based on continuous data (see van der Bijl W. 2018. phylopath: Easy phylogenetic path analysis in R. PeerJ 6:e4718) and are not coded to be compatible with Bayesian mixed models. We think that some of the potential future suggestions (e.g. life history variables), could be well suited with the approach, but are beyond the scope of this current paper.**

Alternatively, the authors could use a model selection approach, testing different combinations of predictors and identifying the models that best explain variation in neophobia.

**The model selection approach was something we wanted to try at the start, but preliminary testing showed that running such large Bayesian models was computationally prohibitive. Furthermore, using a model selection approach hugely increases the potential for cryptic multiple testing and inflated Type I errors (Forstmeier and Schielzeth, 2011). What we have done is (a) run a model selection using a standard LMM (so not phylogenetically corrected) and (b) compared how model variable selection maps on the Bayesian model results. We find that in each case (difference score and raw latency data), that the clear best model (delta AIC<5) was the full model (all parameters included) and that model averaging showed the exact same parameters as significant in the LMM as the Bayesian model. This gives us considerable confidence that even with the addition of phylogeny, we would find the same results. Indeed, it suggests that whilst there is a strong phylogenetic signal, this has no major bearing on the results.**

**Forstmeier W, Schielzeth H. Cryptic multiple hypotheses testing in linear models: overestimated effect sizes and the winner's curse. Behavioral ecology and sociobiology. 2011 Jan;65:47-55.**

Important details of the analytical methods are missing and should be included. For example, the error structures, priors, and any convergence diagnostics used in the MCMCglmm analyses should be described. Latency data, in particular, often do not fit well with Gaussian models, and this limitation should be addressed explicitly.

**Thanks for the reminder. We have included these details in the Methods (lines 899-905) and as additional supplementary material (at the end of this response to reviewers document). In all cases, there were no issues with convergence in the dataset.**

**“For both models (raw and difference score data), to ensure robust convergence diagnostics, we ran three separate chains of the model with different seeds. We assessed the convergence of the MCMC chains using the** **Gelman-Rubin diagnostic from the coda package [113]. The chains were combined into an mcmc.list object and the Gelman-Rubin diagnostic was calculated, which indicated good convergence for all parameters in the model. For both models, the potential scale reduction factors (PSRF) were all close to 1, with upper confidence intervals also near to 1, suggesting that the chains have likely converged well, providing reliable estimates for the model parameters.”**

Finally, certain aspects of the experimental protocol require better justification. If the control condition is intended to equalize feeding motivation, why is it conducted 48 hours apart? Motivation may fluctuate during this interval, introducing potential confounds. Additionally, exposing individuals to a novel object first may have carry-over effects on subsequent feeding motivation, which could inflate repeatability estimates. Including treatment-control order as a covariate could help address this bias.

**We wanted to have some degree of temporal independence of the tests, so the control and test conditions were separated for up to 48 hours (in some cases, for birds that were fed daily, they were separated for 24 hours). Moreover, we tried to maintain time, type and amount of food as similar as possible for both conditions to ensure similar hunger/motivation levels. We have expanded on details relating to this in the Methods section (see below and lines 758-767). We also now include a testing order factor in the models, per your suggestion.**

**“The control trial was conducted within 48 h of the novel object test trial to ensure that hunger/motivation levels were as comparable as possible between the conditions while also accommodating variation in site logistics (e.g. in feeding schedules and subject availability, as some species only fed once per day). Wherever possible, the control trial was conducted on a separate day (except for 36 individuals of 9 species), rather than immediately before or after the novel object test trial, to avoid potential carryover effects such as increased motivation, inhibition or arousal. 85 individuals of 23 species were tested with a longer interval between trials (median: 3 days) due to constraints in the operations of the host facility. However, during this interval, no changes in housing conditions or diet were made to ensure that hunger and motivation levels were as comparable as possible between control and novel object test conditions.”**

In summary, while the manuscript makes a commendable effort to explore neophobia across species, addressing these conceptual and methodological issues will greatly enhance its clarity, rigor, and overall impact. More specific comments are added to the attached pdf version of the manuscript.

**We have addressed all comments provided by the Reviewer with changes marked in the manuscript and in our Response to Reviewers document. We also included responses to the analysis specific comments here below (addressing all other suggested changes/ comments in the manuscript). Once again, thanks very much for your helpful suggestions.**

**ANALYSIS SPECIFIC COMMENTS from Reviewer 2 annotated pdf comments:**

R2: Did you include the control as covariate to model neophobic responses? Otherwise you could be primarily measuring motivation to feed

**We have conducted all analyses with both raw (control, novel object test) and difference score data and we need to see how variables differ between the control and test conditions. However, adding control latencies would not allow this, but rather mirror the difference score analysis. Please note that control and neophobia latencies are independent of one another (as we have clarified in the manuscript, e.g. lines 907-914).**

**“The difference scores are independent of the control latency and comparable between individuals and species. For example, two birds (A, B) may have a similar difference score (e.g. low neophobia), though bird B took longer to approach the familiar food than bird A (longer control latency), reflecting an individual/ species difference in general approach to food (which may be influenced by e.g. hunger, motivation, competition). It does not (necessarily) mean bird B is more neophobic than bird A, therefore both raw and difference score data is informative for the magnitude of difference as well as where this occurs. For example, with the dietary breadth result, both control and test latencies decline at greater breadth, but the declines are greater under test conditions.”**

R2: Why not include site type (field/ captivity)? 13 is not that many, but using site type can still control for confounds.

**Thank you for this suggestion; however, the models used are already large and complex and we do already include “site name” as a random effect (with considerably more variance than site type). We have also added the new factors “social context” and “test order” to help control for confounds, which have also both included more variance (than site type). We assume that the observed effects were not confounded by differences between field/captive studies, though we did not have sufficient power to test this convincingly since n = x and y for wild and captive sites respectively.**

**We also included these discussion points (lines 587-599):**

**“We also provided as many details as possible for each study site (S7 Table), allowing for possible future investigations into the effects of site-specific factors not considered in the present analyses. Nonetheless, in the present study, it was not possible to directly test the effects of captivity on object neophobia due to the relatively small sample of wild species tested (which were also taxonomically skewed). Available evidence suggests that captive bird populations may differ drastically from wild ones regarding various aspects of behaviour and cognition [92], placing an important caveat on studies that, like ours, rely heavily on captive-bred subjects from zoos and laboratories. There is also data suggesting marked differences between species regarding chronic stress in captive settings, which might be pronounced (even if the individuals in question have been raised in captivity) and can be influenced by inconspicuous differences in housing conditions [93-95]. Our results should therefore be considered with these limitations in mind, and we encourage more comparative work explicitly testing effects of captivity on neophobia.”**

R2: single phylogenetic tree: One from the posterior sample or a consensus tree? If the former, you should test the models with several trees to ensure that results are not biased by phylogenetic uncertainty.

**We considered using all 100 generated trees using the multree package. However, multree only allows single random effects, not nested random effects. We used a single consensus tree, and we discuss the potential limitations of this approach in the manuscript (lines 884-889).**

**“For both models, we generated a single consensus phylogenetic tree from birdtree.org [54]; while more updated bird phylogenies exist (e.g. [112]), none of them has yet presented a species-level resolution that can be used with the species in our dataset, causing an issue for the analyses. While differences between newer bird trees and birdtree.org are small, the placement of some orders (most notably Musophagiformes, turacos) do differ; however, the number of potentially ambiguous species (N=3) was small, and our findings were robust to these differences in tree structure.”**

**Given how the LMM model selection performed (see our response to R3 above re: model selection), we are confident that the phylogeny has little impact on the results.**

Model parameters: What about priors and convergence issues?

**We have now included detail of the priors in the methods (lines 875-876) and the convergence tests in Supplementary Materials (end of this document).

“We applied an uninformative prior distribution (*V* = 1, nu = 0.002), and models were run for 100,000 iterations, 1,000 burn-in and a chain thinning of 50.”**

LMMs: I do not understand why you do not use here the same glmm framework used for the main analyses. Also, even if there is interest in assessing differences across species, you still need to control for phylogenetic effects

**We thank the Reviewer for this point and agree there is no need for the LMM model analysis, so we have now carried out only Bayesian mixed model analyses (which control for phylogeny) in the manuscript. Our Methods and Results have been revised to reflect this.**

R2: Obj 2: socio-ecological factors - This does not take into account phylogenetic effects, which is later correctly argued to require correction

**As we state in the Methods (lines 884-889) and text below, we do include consensus phylogeny within the model for the analysis testing ecological factors.**

**“For both models, we generated a single consensus phylogenetic tree from birdtree.org [54]; while more updated bird phylogenies exist (e.g. [112]), none of them has yet presented a species-level resolution that can be used with the species in our dataset, causing an issue for the analyses. While differences between newer bird trees and birdtree.org are small, the placement of some orders (most notably Musophagiformes, turacos) do differ; however, the number of potentially ambiguous species (N=3) was small, and our findings were robust to these differences in tree structure.”**

R2: Obj 2: This was for the first test or also include second and third tests?

**All data was included i.e. all trials and test rounds - we have added this information to the Methods (lines 852-853).**

**“Objective 2 was an avian order comparison of neophobia: that is, we tested whether taxonomic “order” was a predictor of the obtained data (all trials and test rounds available).”**

R2: Obj 1: repeatability - R1: Did you include sex and age?

**We did not know the age and sex of all individuals or were not able to track them within trials - clarified in the Methods section too (lines 847-848).**

**“We did not include sex or age as effects as we did not have this information for all subjects (e.g. zoo/field sites).”**

R2: Obj 1: you can do the same with MCMCglmm. You just need to estimate the intra-class coefficient of individual variation/individual + residual variation

**We have carried out Bayesian mixed model analysis. Whilst we can split the ICC (e.g. for phylogeny), we would need to run a variety of models to account for the levels of repeatability as suggested by Reviewer 3. We think our approach is simpler and easier for the reader to understand.**

R2: This is dangerous. A small species with high metabolic rate will be more motivated to eat and take risk

**Please note that this is a within-species individual-level comparison, so we are not comparing species in this analysis (thus species differences in metabolism should not influence findings). We have added a point in the Discussion regarding body mass as a potential control for species metabolic differences (lines 537-540). Please note there is no body mass effect.**

**“Body mass may also serve as a potential control for motivation as smaller species with higher metabolic rates may need to forage more frequently and thus have shorter latencies for control and novel object test trials. However, we did not find any effect of body mass on neophobia.”**

R2: Flocks coded when couldn’t ID individuals: Was this then considered when modelling neophobic responses, given that being in a group may reduce neophobia?

**Thank you for this suggestion, we have now included this in the model (social context factor).**

Include order variable (control-object; object-control) in model?

**Thank you for this suggestion, we have now included this in the model (test order factor).**

R2: The NT and DN hypotheses both assume that neophobia is a feature of the individual, population or species. If there are no differences in neophobia among individuals, population or species, there is no need to look for explanations. Therefore, I would move the idea that neophobia itself can be plastic after presenting the NTH and DNH, and frame it as a third non-mutually exclusive hypothesis suggesting that neophobia is highly plastic and hence do not show differences among individuals, populations or species. Such plasticity seems to be more connected with the DNH, as the absence of predators or the perception that they do not represent a risk should allow habituation and reduced neophobia. The social effects on neophobia can also be related to the DNH through the dilution effect or other mechanisms.

**We have now expanded the Abstract and Introduction to address these comments. Please note that as per Reviewer 2 suggestion, we re-organised the research objectives, so we are following this order in the Abstract and Introduction too, hence did not move neophobia plasticity after the NTH and DNH section. However, we included your points e.g. in lines 148-151, 170-175, 277-281.**

**Abstract: “We first demonstrated that consistent differences in neophobia existed among individuals, among species and among other taxonomic levels in our dataset, rejecting the null hypothesis that neophobia is highly plastic at all taxonomic levels with no evidence for evolutionary divergence.”**

**Introduction: “However, plasticity in neophobia is also evident, such as when house sparrows (*Passer domesticus*) learn to be less neophobic from conspecifics [17] and rooks (*Corvus frugilegus*) overcome neophobia in groups [18]. Although neophobia can change on a within-individual level, most studies assessing neophobia find that it is fairly consistent within individuals when studied in the same context and developmental stage (but see conflicting data for corvids [18]) and varies widely among different species.”**

**Introduction: “We hypothesised that neophobic responses are consistent across individuals and species; alternatively, responses may be highly plastic, with no consistent differences across individuals, species, or even higher taxonomic groups. We thus tested this hypothesis, noting that species may differ in baseline neophobia despite individual plasticity (i.e. neophobia is a species feature).”**

**Also, regarding sociality and DNH, we have added new point in Introduction (lines 199-203): “Similarly, sociality can also influence neophobia by moderating risk or the dilution effect, as predicted by the Dangerous Niche Hypothesis; for instance, the presence of conspecifics can either enhance or reduce neophobia (e.g. zebra finches, *Taeniopygia guttata,* [12]; common ravens, *Corvus corax* and carrion crows, *Corvus corone,* [33]).”**

**Reviewer #3:**

Review comments on "Evolutionary drivers of neophobia across the avian clade".

This study explored avian species' neophobia across different species by analyzing the effect of phylogenetic variation and ecological drivers that potentially shape species variation of neophobia. The study used a large data set provided by Big Team Science collaborations, which enabled authors to compare neophobia across the species. The results showed that the neophobia varied among species (phylogenetic variation) and species variation of neophobia was explained by some ecological factors such as diet and habitat characteristics. Using such a large dataset including various species is amazing and the results may provide variable insights into our better understanding of ecological and evolutionary mechanisms. I appreciate the authors' huge effort in organizing this big project. At the same time, I also found several serious issues.

First, I don't think this study could correctly explore the evolutionary mechanism of neophobia. Second, the study did not take other important life history traits that may play an important role in shaping species variation of neophobia into account. For these reasons, I think this manuscript needs major fundamental revisions. I will provide major comments rather than minor and point-by-point suggestions because the manuscript may change a lot after addressing comments. I hope these comments will be useful.

**Thank you for your comments, please see our responses below and in the tracked changes manuscript. Please note line numbers correspond with the ‘tracked changes’ manuscript version.**

Major comments;

1.	Evolutionary mechanism was not correctly explored

The main aim of this study is to examine the evolutionary mechanism of neophobia. However, as far as I understand from the manuscript, the study could not examine the evolutionary mechanism of neophobia for a number of reasons. First, this study focuses on phylogeny in objective 1. However, the analysis tested the variation of neophobia at the genus level, while the evolutionary pattern was not correctly analyzed. Variation between the genus does not directly mean the evolution of this trait. I am not a specialist of this type of analysis, but there might be some statistical methods to address this type of question.

**We believe that this Reviewer has misunderstood our analyses as we tested repeatability at the individual level. Phylogeny at the species level was included in the Bayesian mixed model analysis - we are not certain where genus level came from. Phylogenetic signal is calculated from the species-level phylogeny. We have now also included repeatability at the species, family and order level, as suggested below.**

Second, the result from the within-individual repeatability over the shorter period does not fully support the evolutionary mechanism. Repeatability is more likely to be found from a shorter test interval compared to a longer interval (Bell et al. 2009 Animal Behaviour). Since some avian species used in this study may have longer lifespans, so within-few weeks repeatability may reflect their behavioral tendency over the study period. At least, species lifespan should be considered. Heritability of this trait should support some aspect of microevolution, but may not be a between-species level evolution.

**We thank the Reviewer for raising this interesting point. Repeatability does appear to be higher over shorter time spans for some behaviours, but this is not always the case. Repeatability depends on a number of factors, including the behaviours in question and the number of observations in any given time frame (Tkaczynski et al, 2020, RSOS). Lifespan is certainly a valid consideration in addressing this question, although results of some previous studies indicate behaviour to be relatively consistent across different age classes, even in species with vastly differing lifespans.**

**We expand on the repeatability points in lines 441-448:**

**“There may be some groups of birds where responses are less repeatable due to factors such as rapid learning i.e. habituation to novelty, so studies focusing on species within those taxonomic groups may be less likely to find repeatability. However, taxonomic distribution in our data limits testing this with our sample. Variation in neophobia repeatability may also be due to factors such as developmental and social influences [69-71]. Repeatability can also vary temporally. While many studies, including ours, find high repeatability across shorter timespans [56], this is not always the case; variation in results can depend on the behaviour measured and the number and frequency of measures recorded within designated timeframes [72].”**

**Furthermore, please see our response to other comments (including your point below under 2.) regarding the inclusion of other life history variables in this manuscript, including new related points in Introduction and Discussion.**

**Namely - we completely agree that broader/ other life history factors would be an interesting focus and question for a future paper, but in this case, our focus is primarily on ecological factors for this paper (e.g. per pre-registration), hence we refer to these as ecological factors, and we now remove “social” for clarity.**

In addition, the repeatability was calculated using all data, representing overall neophobia repeatability. This did not consider species variation. So, I am not able to understand the biological meaning of this repeatability.

**Thank you for your very useful suggestion. As suggested, we have now calculated repeatability at individual, species, family and order level (see individual repeatability section in Results and new Supplementary Table), which we think adds another useful perspective.**

2.	Other key life history traits were not accounted for.

I understand social, diet, and habitat factors are important drivers for neophobia variation. In addition to these, longevity, developmental speed, reproductive strategies (r-K strategies), and other cognitive characteristics such as innovation may also be factors that can be strongly associated with neophobia. However, these variables were not considered in the analysis. Additionally, brain size may also play an important role in neophobia. The risk-taking behavior measured by flight initiation distance was associated with species' brain size, and I assume neophobia which is also a risk-taking behavior may be associated with species brain size throughout influencing species variation of cognitive mechanisms. I would strongly recommend taking into account these key life history variables.

**We appreciate these suggestions and agree that this would be very interesting. However, it is beyond the scope and focus of this paper, where we primarily focus on ecological drivers. Reviewer 1 suggested that we split this paper into multiple papers, given how much we already cover, though the Editors asked us not to follow this suggestion. We see the testing of other/ broader life history drivers as a potential future study (e.g. potentially with a subset of species from our dataset) and we have included this point in the Discussion (lines 636-645). We also expand on Life History Theory in the Introduction (Table 1 and below; lines 186-192).**

**Introduction: “Neophobia should also be shaped by life history traits. Life History Theory predicts that species with slow life histories (e.g. long-life expectancy, long reproductive lifespans, slower growth) would be more risk-averse and thus neophobic, while species with fast life histories (e.g. short life expectancy, high fecundity, faster growth) would be likely risk-takers [26,27]. This framework may help to explain associations between neophobia and species traits such as body size and domestication status [28,29], although a more thorough test of the predictions of Life History Theory would require comparing neophobia in a wider range of taxa than has been done thus far.”**

**Discussion: “Body mass and domestication status were the only life history-related predictors coded and tested with our full dataset, with no effect found for body mass and domesticated birds showing lower neophobia, as predicted by Life History Theory and previous work on domesticated birds [29]. Although additional variables (e.g. clutch size) could have been added to this analysis, there was a trade-off between maximizing the value of this particular dataset and the risk of finding false positives from running many analyses, especially considering that we were already examining raw data and differences score data for each variable, both with and without domesticated species. Future studies on object neophobia should focus more on the influence of life history factors such as longevity, developmental speed and reproductive strategy (r/K strategies). Furthermore, potential effects of neurological variables may be explored, which we did not consider here due to the ecological focus of this study.”**

3.	Does Neophobia Threshold Hypothesis explain the evolutionary mechanism of neophobia?

The life history variables were selected based on the two main hypotheses in this study: (1) Neophobia Threshold Hypothesis and (2) Dangerous Niche Hypothesis. While the Dangerous Niche Hypothesis explains how predation pressure acts as an evolutionary driver that shapes neophobia, the Neophobia Threshold Hypothesis explains how neophobia is the driver that shapes other life history traits. Therefore, as far as I understand from this manuscript, the Neophobia Threshold Hypothesis may not explain the evolutionary mechanism of neophobia. Although neophobia can be feedback to shaping dietary and habitat preference, this is not explained in the manuscript.

**We have now more explicitly expanded on these three main hypotheses in the Introduction, Discussion and Table 1.**

4.	Flexibility vs consistency

Neophobia was explained as one of the flexible behavioural types. However, this study tested repeatability and found significant within-individual consistency at least over the study period. This means that neophobia is not a flexible behaviour. Is neophobia really flexible behaviour? There is also no explanation in the Discussion section about microevolution and how it is related to evolution at the species scale.

**We note that repeatability is tested and found at the individual level, though neophobia differs between individuals and species. We have expanded on this point in the Discussion as follows (lines 453-454): “****Thus, object neophobia appears to be consistent within individual birds (at least for the short time spans tested in our study), while it is less repeatable among species.” During revisions, we removed reference to microevolution.**

**Further examples where we have clarified this difference between mean neophobia within species reflecting flexibility differences among species vs repeatability, which is negatively correlated to flexibility within a species (Introduction, lines 170-175): “However, plasticity in neophobia is also evident, such as when house sparrows (*Passer domesticus*) learn to be less neophobic from conspecifics [17] and rooks (*Corvus frugilegus*) overcome neophobia in groups [18]. Although neophobia can change on a within-individual level, most studies assessing neophobia find that it is fairly consistent within individuals when studied in the same context and developmental stage (but see conflicting data for corvids [18]) and varies widely among different species. “**

Specific comments;

-Statistical analysis; Response variables for statistical analyses were only "latency to touch familiar food", however, "difference between control and test conditions" may be appropriate because it controlled baseline motivation to food conditioning.

**We used both raw data and difference scores for both objectives. We agree that difference scores help to control for potential baseline motivation. Similarly, objective 1 is a within-individual comparison, thus baseline motivation is expected to be relatively consistent in the 24-48 hour test period, where subjects receive food and testing at the same time per day.**

- In the model for objective 2, what were the fixed effects? I also wonder how the authors tested model validation. Please clarify these in the main text.

**For objective 2: “The response variable was “latency to touch familiar food”, with the fixed effect of taxonomic “order” and the random effect of “individual”.” This model has been changed to a Bayesian model now. As per Reviewer 2 suggestion, we carried out tests for model convergence and included this in the Methods section (see lines 853-855).**

**“For both models (raw and difference score data), to ensure robust convergence diagnostics, we ran three separate chains of the model with different seeds. We assessed the convergence of the MCMC chains using the Gelman-Rubin diagnostic from the coda package [113]. The chains were combined into an mcmc.list object and the Gelman-Rubin diagnostic was calculated, which indicated good convergence for all parameters in the model. For both models, the potential scale reduction factors (PSRF) were all close to 1, with upper confidence intervals also near to 1, suggesting that the chains have likely converged well, providing reliable estimates for the model parameters.” (Discussion, lines 899-905).**

-More seriously, the repeatability model did not include any fixed effect meaning the repeatability value is unadjusted. Ecological factors should be considered for repeatability.

**We have now redone this analysis at individual, species, family and order level (see Results section and Supplementary Materials). As per your suggestion, this now also includes the ecological factors as fixed effects within the analysis (see Results section, lines 328-330).**

**“Furthermore, repeatability was found when all ecological and other factors were included as fixed effects in the models (control: R= 0.48 [0.43/0.52], p<0.001; novel object test: R= 0.61 [0.57/0.65], p<0.001; difference scores: R= 0.27 [0.21/0.32], p<0.001).”**

-L 212; This is not only this part but throughout the manuscript, socio-ecological factors should be reworded. The social factor is one of the ecological factors. On the other hand, "socio-ecological factor" refers to the factors that are related to human society and ecological factors in ecology. I would suggest rewording to life history factors.

**We completely agree that broader/ other life history factors would be an interesting focus and question for a future paper, but in this case, our focus is primarily on ecological factors for this article (and asper our pre-registration), hence we refer to these as ecological factors, and we now remove “social” for clarity. We have also changed the title in this regard (from “evolutionary” to “ecological”).**

L223: This sentence can be more simple and merged with the previous paragraph.

**We agree and have changed it accordingly.**

L287; I am not able to understand why testing short-term repeatability can estimate the microevolution of neophobia. Considering the much longer life span for most avian species, using repeatability at the shorter time scale (~2 weeks) seems not appropriate.

**We removed “microevolution” from the manuscript, given we only refer to it briefly and it is causing confusion.**

**We have also added further clarification to the Introduction, lines 275-281: “For objective 1, we wanted to establish the extent to which there are consistent individual differences in neophobia by measuring repeatability, which sets the upper limit to heritability - an important component of the evolvability of a trait within a population. We hypothesised that neophobic responses are consistent across individuals and species; alternatively, responses may be highly plastic, with no consistent differences across individuals, species, or even higher taxonomic groups. We thus tested this hypothesis, noting that species may differ in baseline neophobia despite individual plasticity (i.e. neophobia is a species feature).”**

**We have expanded in the Discussion section on the timeframe selection and implications (see lines 445-448): “Repeatability can also vary temporally. While many studies, including ours, find high repeatability across shorter timespans [56], this is not always the case; variation in results can depend on the behaviour measured and the number and frequency of measures recorded within designated timeframes [72].”**

**ADDITIONAL INFORMATION FOR REVIEWERS (particularly reviewer 2 in response to comments copied in below):**

R2 comment: “Important details of the analytical methods are missing and should be included. For example, the error structures, priors, and any convergence diagnostics used in the MCMCglmm analyses should be described.”

**Thanks for the reminder. We have included these details in the Methods (lines 899-905) and as additional supplementary material (see below). In all cases, there were no issues with convergence in the dataset.**

**“For both models (raw and difference score data), to ensure robust convergence diagnostics, we ran three separate chains of the model with different seeds. We assessed the convergence of the MCMC chains using the Gelman-Rubin diagnostic from the coda package [113]. The chains were combined into an mcmc.list object and the Gelman-Rubin diagnostic was calculated, which indicated good convergence for all parameters in the model. For both models, the potential scale reduction factors (PSRF) were all close to 1, with upper confidence intervals also near to 1, suggesting that the chains have likely converged well, providing reliable estimates for the model parameters.”**

R2 comment: “Model parameters: What about priors and convergence issues?”

**We have now included detail of the priors in the methods (lines 875-876) and the convergence tests in Supplementary Materials (end of this document).

“We applied an uninformative prior distribution (*V* = 1, nu = 0.002), and models were run for 100,000 iterations, 1,000 burn-in and a chain thinning of 50.”**

Table SM1: (a) Model selection showing models with ∆AIC<10, model weight and log likelihood. Variable numbers are given in Table S2. The full model has highest support. (b) Model-coefficients (conditional average) from the best model subset (∆AICc <10). Significant parameters are shown in bold.

(a)

| **Variables** | **df** | **logLik** | **AICc** | **∆AICc** | **Model weight** |
| --- | --- | --- | --- | --- | --- |
| 1+2+3+4+5+6+7+8+9+10 | 17 | -17483.68 | 35001.63 | 0 | 0.82 |
| 1+2+3+4+5+6+8+9+10 | 16 | -17487.42 | 35007.08 | 5.45 | 0.05 |
| 1+2+4+5+6+7+8+9+10 | 16 | -17487.83 | 35007.89 | 6.26 | 0.04 |
| 1+2+3+4+5+6+7+8+10 | 16 | -17487.92 | 35008.08 | 6.45 | 0.03 |
| 1+2+3+4+5+7+8+9+10 | 16 | -17488.09 | 35008.41 | 6.78 | 0.03 |

(b)

| **Variable no.** | **Variable name** | **Estimate** | **SE** | **z** | **p** |
| --- | --- | --- | --- | --- | --- |
|  | (Intercept) | 407.58 | 221.67 | 1.84 | 0.066 |
| 1 | Urban Habitat Use | -79.33 | 67.21 | 1.18 | 0.238 |
| 2 | Order of presentation (trial second) | -65.23 | 18.39 | 3.55 | **<0.001** |
| 2 | Order of presentation (same day) | -107.06 | 55.18 | 1.94 | 0.052 |
| 3 | Condition | -6.40 | 24.35 | 0.26 | 0.793 |
| 4 | Dietary Breadth | 130.14 | 122.65 | 1.06 | 0.289 |
| 5 | Domestication Status | 73.30 | 46.45 | 1.58 | 0.115 |
| 6 | Habitat Density | -19.79 | 20.01 | 0.99 | 0.323 |
| 7 | Habitat Specialist | -2.08 | 16.67 | 0.13 | 0.901 |
| 8 | log(Body Mass) | -92.56 | 165.41 | 0.56 | 0.576 |
| 9 | Migration (Nomadic) | -163.62 | 76.84 | 2.13 | **0.033** |
| 9 | Migration (Non-Migratory) | 6.17 | 27.03 | 0.23 | 0.819 |
| 10 | Social Context | -133.97 | 94.99 | 1.41 | 0.159 |
| 11 | Territoriality (Seasonal) | -109.84 | 94.01 | 1.17 | 0.243 |
| 11 | Territoriality (Year-Round) | 407.58 | 221.67 | 1.84 | 0.066 |

Table SM2: (a) Model selection showing models with ∆AIC<10, model weight and log likelihood. Variable numbers are given in Table S2. The full model has highest support. (b) model-coefficients (conditional average) from the best model subset (∆AICc <10). Significant parameters are shown in bold.

(a)

| **Variables** | **df** | **logLik** | **AICc** | **∆AICc** | **Model weight** |
| --- | --- | --- | --- | --- | --- |
| 1+2+3+4+5+6+7+8+9+10+11+12+13+14+15+16+17+18+19+20+21 | 31 | -34562.88 | 69188.18 | 0 | 0.9 |
| 1+2+3+4+5+6+7+8+9+10+11+12+13+14+15+16+17+19+20+21 | 30 | -34566.69 | 69193.78 | 5.6 | 0.05 |
| 1+2+3+4+5+6+7+8+9+10+11+12+13+14+15+16+18+19+20+21 | 30 | -34567.46 | 69195.31 | 7.14 | 0.03 |
| 1+2+3+4+5+6+7+8+9+10+11+13+14+15+16+17+18+19+20+21 | 30 | -34567.61 | 69195.6 | 7.43 | 0.02 |

(b)

| **Variable no.** | **Variable name** | **Estimate** | **SE** | **z** | **p** |
| --- | --- | --- | --- | --- | --- |
|  | (Intercept) | 344.92 | 160.62 | 2.15 | **0.032** |
| 1 | Urban Habitat Use | -65.57 | 48.78 | 1.34 | 0.179 |
| 2 | Order of presentation (trial second) | 30.57 | 15.94 | 1.92 | 0.055 |
| 2 | Order of presentation (same day) | -4.32 | 50.21 | 0.09 | 0.932 |
| 3 | Condition | 28.69 | 69.51 | 0.41 | 0.680 |
| 4 | Dietary Breadth | 2.31 | 17.60 | 0.13 | 0.896 |
| 5 | Domestication Status | 37.53 | 87.12 | 0.43 | 0.667 |
| 6 | Habitat Density | -9.85 | 33.77 | 0.29 | 0.771 |
| 7 | Habitat Specialist | 6.82 | 14.57 | 0.47 | 0.640 |
| 8 | log(Body Mass) | -28.43 | 12.06 | 2.36 | **0.018** |
| 9 | Migration (Nomadic) | 266.06 | 120.31 | 2.21 | 0.027 |
| 9 | Migration (Non-Migratory) | -99.71 | 55.79 | 1.79 | 0.074 |
| 10 | Social Context | 181.04 | 23.73 | 7.63 | <0.001 |
| 11 | Territoriality (Seasonal) | -58.89 | 68.67 | 0.86 | 0.391 |
| 11 | Territoriality (Year-Round) | 15.74 | 68.27 | 0.23 | 0.818 |
| 12 | Condition x Urban habitat use | -26.38 | 23.63 | 1.12 | 0.264 |
| 13 | Condition x Test Presentation (trial second) | 7.24 | 25.72 | 0.28 | 0.778 |
| 13 | Condition x Presentation (same day) | 62.84 | 44.32 | 1.42 | 0.156 |
| 14 | Condition x Dietary Breadth | -29.32 | 7.78 | 3.77 | **<0.001** |
| 15 | Condition x Domestication | 126.68 | 24.00 | 5.28 | **<0.001** |
| 16 | Condition x Habitat Density | 41.53 | 17.02 | 2.44 | **0.015** |
| 17 | Condition x Habitat Specialist | 12.00 | 6.39 | 1.88 | 0.061 |
| 18 | Condition x log(Body Mass) | 8.38 | 5.47 | 1.53 | 0.125 |
| 19 | Condition x Migration (Nomadic) | -65.87 | 80.51 | 0.82 | 0.413 |
| 19 | Condition x Migration (Non-migratory) | -45.50 | 27.58 | 1.65 | 0.099 |
| 20 | Condition x Social Context | 156.36 | 20.54 | 7.61 | **<0.001** |
| 21 | Condition x Territoriality (Seasonal) | 39.06 | 33.03 | 1.18 | 0.237 |
| 21 | Condition x Territoriality (Year-Round) | -43.00 | 32.21 | 1.34 | 0.182 |

**Bayesian Model checking and diagnostics (difference model model)**

To ensure robust convergence diagnostics, we ran three separate chains of the model with different seeds. We assessed the convergence of the MCMC chains using the Gelman-Rubin diagnostic from the coda package. The chains were combined into an mcmc.list object and the Gelman-Rubin diagnostic was calculated.

The Gelman-Rubin diagnostic indicated good convergence for all parameters in the model. The potential scale reduction factors (PSRF) were close to 1, with upper confidence intervals also near 1, suggesting that the chains have likely converged well. providing reliable estimates for the model parameters. Density and trace plots are shown below.

**Bayesian Model checking and diagnostics (latency to touch food model)**

To ensure robust convergence diagnostics, we ran three separate chains of the model with different seeds. We assessed the convergence of the MCMC chains using the Gelman-Rubin diagnostic from the *coda* package (). The chains were combined into an *mcmc.list* object and the Gelman-Rubin diagnostic was calculated.

The Gelman-Rubin diagnostic indicated good convergence for all parameters in the model. The potential scale reduction factors (PSRF) were close to 1, with upper confidence intervals also near 1, suggesting that the chains have likely converged well. providing reliable estimates for the model parameters. Density and trace plots are shown below.
