# Supplementary material for: A large-scale study across the avian clade identifies ecological drivers of neophobia
Source: PLoS Biol. 2025 Oct 14;23(10):e3003394. doi: 10.1371/journal.pbio.3003394 (PMC12520376; doi:10.1371/journal.pbio.3003394)
Supplement: Attachment — Submitted filename: Response to Editor .docx [file pbio.3003394.s005.docx]

**PBIOLOGY-D-24-03430R3**

**MB1 revision 2: Due 4th Sept 2025**

**Response to Editor:**

Dear Dr Miller,

Thank you for your patience while we considered your revised manuscript "Ecological drivers of neophobia across the avian clade" for publication as a Initial Research Submission at PLOS Biology. This revised version of your manuscript has been evaluated by the PLOS Biology editors, the Academic Editor and two of the original reviewers.

Based on the reviews, we are likely to accept this manuscript for publication, provided you satisfactorily address the remaining points raised by the reviewers and the following data and other policy-related requests.

IMPORTANT - please attend to the following:

a) Please make your Title more explicit and declarative. We suggest that you change it to "A large-scale study across the avian clade identifies ecological drivers of neophobia"

**Done**

b) Please address the remaining requests from reviewer #1.

**Done**

c) Many thanks for providing the funding information in Table S9. However, these need to be included in the Financial Disclosure statement (I counted maybe 14 funding bodies listed in that table); please do so.

**Done - added to manuscript, submission portal and copied below.**

**“FWF START Project Y 1309 to Alice Auersperg, Consejo Nacional de Investigaciones Científicas y Técnicas (PIP 2022-2024 GI 11220210100521CO) to Raul Gomez, Natural Sciences and Engineering Council of Canada NSERC RGPIN-2019-04733, NSERC Discovery Grant Launch Supplement, University of Alberta Faculty of Science Start-up Grant to Lauren Guillette, National Science Foundation IOS-2237423 to Christine Lattin, Beca Aves Argentinas 2022 to Jimena Lois-Milevicich, Research Support Development Grant, Anglia Ruskin University to Rachael Miller, Swedish Research Council (Vetenskapsrådet) grant 2021-01650 to Stephan Reber, Agencia Nacional de Promoción de la Investigación, el Desarrollo Tecnológico y la Innovación PICT 2019-00381 to Juan Reboreda, National Science Foundation IOS: 2207395 to Christopher Templeton, Marie Skłodowska-Curie Action fellowship ‘UrbanCog’ Project No. 101062662‬ under the European Union’s Horizon Europe Programme (**[**https://marie-sklodowska-curie-actions.ec.europa.eu**](https://marie-sklodowska-curie-actions.ec.europa.eu)**) to Camille Troisi, Swedish Research Council (Vetenskapsrådet) International postdoc grant (2020-00719 ), and DR. J.L. DOBBERKE FOUNDATION (Dobberke/2349/202115) to Utku Urhan, ERC Consolidator grant (European Union’s Horizon 2020 research and innovation programme, grant agreement No. 769595), and Methusalem Project 01M00221 (Ghent University) to Frederick Verbruggen, BOF postdoc fellowship (#BOF.PDO.2021.0035.01) to Alizee Vernouillet and Mitacs Globalink Research Internship 2022 Project ID: 25251 to Karen Yeung.”**

d) Please address my Data Policy requests below; specifically, we need you to supply the numerical values underlying Figs 1 (treefile), 2 (treefile), 3, 4, 5, S1, S2, S3, S4, either as a supplementary data file or as a permanent DOI’d deposition. I note that you already have an associated Figshare deposition, but this seems to mostly contain metadata and raw data. Please could you complete this deposition with the data and code needed to recreate the Figures?

**Done. We have uploaded all relevant values and coding scripts for figures via our Figshare repository and published the link to secure the permanent DOI, as below. Please note that Fig 1 was manually vectorized in Illustrator, then the bird drawings were added, with the bird orders and number of species coming from the final dataset available on Figshare. Fig 6 does not include any values.**

**We updated the data availability text as follows:**

**“The full data set (including species with 600 second trial cut off), final analysis data set (removing species with 600 second trial cut off) and R coding script are available on: Figshare DOI: 10.6084/m9.figshare.27324972. We have also included the numerical values and R coding scripts underlying all relevant figures (available via this Figshare link).”**

**We also added to the Fig 2 legend: “The data underlying this figure can be found in DOI: 10.6084/m9.figshare.27324972”.**

e) Please cite the location of the data clearly in all relevant main and supplementary Figure legends, e.g. “The data underlying this Figure can be found in S1 Data” or “The data underlying this Figure can be found in <https://eur03.safelinks.protection.outlook.com/?url=https%3A%2F%2Ffigshare.com%2FXXXXXXXX&data=05%7C02%7Cr.miller7%40exeter.ac.uk%7Cfa78930fde1540abab6808ddd69e3616%7C912a5d77fb984eeeaf321334d8f04a53%7C0%7C0%7C638902697193290879%7CUnknown%7CTWFpbGZsb3d8eyJFbXB0eU1hcGkiOnRydWUsIlYiOiIwLjAuMDAwMCIsIlAiOiJXaW4zMiIsIkFOIjoiTWFpbCIsIldUIjoyfQ%3D%3D%7C0%7C%7C%7C&sdata=egI91FtdvmJEywauwbMmPNdTecgMv5DEUx28MPISz6U%3D&reserved=0>

**Done. We have added the following text to all relevant main and supplementary figure legends: “The data underlying this figure can be found in DOI: 10.6084/m9.figshare.27324972”.**

f) Please make any custom code available, either as a supplementary file or as part of your data deposition.

**Done. Added to our Figshare link (included above and below). We had also ensured that all figures are uploaded in TIFF format (including supplementary figures) to the submission portal.**

As you address these items, please take this last chance to review your reference list to ensure that it is complete and correct. If you have cited papers that have been retracted, please include the rationale for doing so in the manuscript text, or remove these references and replace them with relevant current references. Any changes to the reference list should be mentioned in the cover letter that accompanies your revised manuscript.

**Done. We have conducted a full check of references list and confirmed there are no retracted papers to remove.**

In addition to these revisions, you may need to complete some formatting changes, which you will receive in a follow up email. A member of our team will be in touch with a set of requests shortly. If you do not receive a separate email within a few days, please assume that checks have been completed, and no additional changes are required.

We expect to receive your revised manuscript within two weeks. EXTENDED DEADLINE TO 4TH SEPT - APPROVED

To submit your revision, please go to <https://eur03.safelinks.protection.outlook.com/?url=https%3A%2F%2Fwww.editorialmanager.com%2Fpbiology%2F&data=05%7C02%7Cr.miller7%40exeter.ac.uk%7Cfa78930fde1540abab6808ddd69e3616%7C912a5d77fb984eeeaf321334d8f04a53%7C0%7C0%7C638902697193305996%7CUnknown%7CTWFpbGZsb3d8eyJFbXB0eU1hcGkiOnRydWUsIlYiOiIwLjAuMDAwMCIsIlAiOiJXaW4zMiIsIkFOIjoiTWFpbCIsIldUIjoyfQ%3D%3D%7C0%7C%7C%7C&sdata=JFKtIRFydEWJEjHV29rX0uF7DN8z10XLHFgD2A24PDs%3D&reserved=0> and log in as an Author. Click the link labelled 'Submissions Needing Revision' to find your submission record. Your revised submission must include the following:

- a cover letter that should detail your responses to any editorial requests, if applicable, and whether changes have been made to the reference list

- a Response to Reviewers file that provides a detailed response to the reviewers' comments (if applicable, if not applicable please do not delete your existing 'Response to Reviewers' file.)

- a **track-changes file** indicating any changes that you have made to the manuscript.

NOTE: If Supporting Information files are included with your article, note that these are not copyedited and will be published as they are submitted. Please ensure that these files are legible and of high quality (at least 300 dpi) in an easily accessible file format. For this reason, please be aware that any references listed in an SI file will not be indexed. For more information, see our Supporting Information guidelines:

<https://eur03.safelinks.protection.outlook.com/?url=https%3A%2F%2Fjournals.plos.org%2Fplosbiology%2Fs%2Fsupporting-information&data=05%7C02%7Cr.miller7%40exeter.ac.uk%7Cfa78930fde1540abab6808ddd69e3616%7C912a5d77fb984eeeaf321334d8f04a53%7C0%7C0%7C638902697193315511%7CUnknown%7CTWFpbGZsb3d8eyJFbXB0eU1hcGkiOnRydWUsIlYiOiIwLjAuMDAwMCIsIlAiOiJXaW4zMiIsIkFOIjoiTWFpbCIsIldUIjoyfQ%3D%3D%7C0%7C%7C%7C&sdata=KF%2F2aw%2BNAXer2oq8YnrUkU2NwBVmbdSZTOynZ2RH%2FPs%3D&reserved=0>

*Published Peer Review History*

Please note that you may have the opportunity to make the peer review history publicly available. The record will include editor decision letters (with reviews) and your responses to reviewer comments. If eligible, we will contact you to opt in or out. Please see here for more details:

<https://eur03.safelinks.protection.outlook.com/?url=https%3A%2F%2Fplos.org%2Fpublished-peer-review-history%2F&data=05%7C02%7Cr.miller7%40exeter.ac.uk%7Cfa78930fde1540abab6808ddd69e3616%7C912a5d77fb984eeeaf321334d8f04a53%7C0%7C0%7C638902697193324933%7CUnknown%7CTWFpbGZsb3d8eyJFbXB0eU1hcGkiOnRydWUsIlYiOiIwLjAuMDAwMCIsIlAiOiJXaW4zMiIsIkFOIjoiTWFpbCIsIldUIjoyfQ%3D%3D%7C0%7C%7C%7C&sdata=X9xfSdOn3dFzpKvqxdDQtN6bjp32lLGQJEtfNbnC9fU%3D&reserved=0>

*Press*

Should you, your institution's press office or the journal office choose to press release your paper, please ensure you have opted out of Early Article Posting on the submission form. We ask that you notify us as soon as possible if you or your institution is planning to press release the article.

**BLURB (500 characters max) - uploaded to submission portal**

“Neophobia is the fear of novelty and has an impact on adaptability and survival. Neophobia varies considerably across species and individuals, but exploring wider evolutionary drivers was hampered by lack of standardised, comparative multi-species studies. Through the ManyBirds Project, we combined resources across 129 collaborators, 77 institutions to test neophobia in 1400 subjects, 136 species across 25 orders. We identified phylogenetic influences and broad ecological drivers of neophobia.”

*Protocols deposition*

To enhance the reproducibility of your results, we recommend that if applicable you deposit your laboratory protocols in protocols.io, where a protocol can be assigned its own identifier (DOI) such that it can be cited independently in the future. Additionally, PLOS ONE offers an option for publishing peer-reviewed Lab Protocol articles, which describe protocols hosted on protocols.io. Read more information on sharing protocols at <https://eur03.safelinks.protection.outlook.com/?url=https%3A%2F%2Fplos.org%2Fprotocols%3Futm_medium%3Deditorial-email%26utm_source%3Dauthorletters%26utm_campaign%3Dprotocols&data=05%7C02%7Cr.miller7%40exeter.ac.uk%7Cfa78930fde1540abab6808ddd69e3616%7C912a5d77fb984eeeaf321334d8f04a53%7C0%7C0%7C638902697193334405%7CUnknown%7CTWFpbGZsb3d8eyJFbXB0eU1hcGkiOnRydWUsIlYiOiIwLjAuMDAwMCIsIlAiOiJXaW4zMiIsIkFOIjoiTWFpbCIsIldUIjoyfQ%3D%3D%7C0%7C%7C%7C&sdata=dMunqwBqvf%2BICZHaYJGhsC%2FsFHIueaqI5ZqbdK0auIE%3D&reserved=0>

Please do not hesitate to contact me should you have any questions.

Sincerely,

Roli Roberts

Roland Roberts, PhD

Senior Editor

rroberts@plos.org

PLOS Biology

------------------------------------------------------------------------

DATA POLICY:

You may be aware of the PLOS Data Policy, which requires that all data be made available without restriction: <https://eur03.safelinks.protection.outlook.com/?url=http%3A%2F%2Fjournals.plos.org%2Fplosbiology%2Fs%2Fdata-availability&data=05%7C02%7Cr.miller7%40exeter.ac.uk%7Cfa78930fde1540abab6808ddd69e3616%7C912a5d77fb984eeeaf321334d8f04a53%7C0%7C0%7C638902697193344336%7CUnknown%7CTWFpbGZsb3d8eyJFbXB0eU1hcGkiOnRydWUsIlYiOiIwLjAuMDAwMCIsIlAiOiJXaW4zMiIsIkFOIjoiTWFpbCIsIldUIjoyfQ%3D%3D%7C0%7C%7C%7C&sdata=upFvw2xLjARKnNlKZHlhuddJ0uOb6cLEv9%2BolYTyqGo%3D&reserved=0>. For more information, please also see this editorial: <https://eur03.safelinks.protection.outlook.com/?url=http%3A%2F%2Fdx.doi.org%2F10.1371%2Fjournal.pbio.1001797&data=05%7C02%7Cr.miller7%40exeter.ac.uk%7Cfa78930fde1540abab6808ddd69e3616%7C912a5d77fb984eeeaf321334d8f04a53%7C0%7C0%7C638902697193353856%7CUnknown%7CTWFpbGZsb3d8eyJFbXB0eU1hcGkiOnRydWUsIlYiOiIwLjAuMDAwMCIsIlAiOiJXaW4zMiIsIkFOIjoiTWFpbCIsIldUIjoyfQ%3D%3D%7C0%7C%7C%7C&sdata=V88PgSeiuIrn7ymt7HcydmcG3r0EPn5hTJKVY4t%2B9j8%3D&reserved=0>

Note that we do not require all raw data. Rather, we ask that all individual quantitative observations that underlie the data summarized in the figures and results of your paper be made available in one of the following forms:

**1) Supplementary files (e.g., excel). Please ensure that all data files are uploaded as 'Supporting Information' and are invariably referred to (in the manuscript, figure legends, and the Description field when uploading your files) using the following format verbatim: S1 Data, S2 Data, etc. Multiple panels of a single or even several figures can be included as multiple sheets in one excel file that is saved using exactly the following convention: S1_Data.xlsx (using an underscore).**

**Done - All of the relevant data files are available via our published DOI figshare link (15 files). We refer to this link in several places in the manuscript for clarity, including the data availability section and in all relevant figure legends.**

2) Deposition in a publicly available repository. Please also provide the accession code or a reviewer link so that we may view your data before publication.

**Done - already available via Figshare link, copied below from manuscript, now published so we have a permanent DOI and have removed the private link.**

**“The full data set (including species with 600 second trial cut off), final analysis data set (removing species with 600 second trial cut off) and R coding script are available on: Figshare DOI: 10.6084/m9.figshare.27324972”**

Regardless of the method selected, please ensure that you provide the individual numerical values that underlie the summary data displayed in the following figure panels as they are essential for readers to assess your analysis and to reproduce it: Figs 1 (treefile), 2 (treefile), 3, 4, 5, S1, S2, S3, S4. NOTE: the numerical data provided should include all replicates AND the way in which the plotted mean and errors were derived (it should not present only the mean/average values).

**Done - please see related response above**

IMPORTANT: Please also ensure that figure legends in your manuscript include information on where the underlying data can be found, and ensure your supplemental data file/s has a legend.

Please ensure that your Data Statement in the submission system accurately describes where your data can be found.

**Done**

------------------------------------------------------------------------

CODE POLICY

Per journal policy, if you have generated any custom code during the course of this investigation, please make it available without restrictions. Please ensure that the code is sufficiently well documented and reusable, and that your Data Statement in the Editorial Manager submission system accurately describes where your code can be found.

Please note that we cannot accept sole deposition of code in GitHub, as this could be changed after publication. However, you can archive this version of your publicly available GitHub code to Zenodo. Once you do this, it will generate a DOI number, which you will need to provide in the Data Accessibility Statement (you are welcome to also provide the GitHub access information). See the process for doing this here: <https://eur03.safelinks.protection.outlook.com/?url=https%3A%2F%2Fdocs.github.com%2Fen%2Frepositories%2Farchiving-a-github-repository%2Freferencing-and-citing-content&data=05%7C02%7Cr.miller7%40exeter.ac.uk%7Cfa78930fde1540abab6808ddd69e3616%7C912a5d77fb984eeeaf321334d8f04a53%7C0%7C0%7C638902697193363286%7CUnknown%7CTWFpbGZsb3d8eyJFbXB0eU1hcGkiOnRydWUsIlYiOiIwLjAuMDAwMCIsIlAiOiJXaW4zMiIsIkFOIjoiTWFpbCIsIldUIjoyfQ%3D%3D%7C0%7C%7C%7C&sdata=zg6RE2Z%2B0rhEn7OeraCe0h3hvglxMC%2FsK7FFUf8j7tc%3D&reserved=0>

------------------------------------------------------------------------

DATA NOT SHOWN?

- Please note that per journal policy, we do not allow the mention of "data not shown", "personal communication", "manuscript in preparation" or other references to data that is not publicly available or contained within this manuscript. Please either remove mention of these data or provide figures presenting the results and the data underlying the figure(s).

------------------------------------------------------------------------

**REVIEWERS' COMMENTS:**

**Reviewer #1:**

The authors have worked hard to address my and the other reviewers' comments and it's great to see a very much improved version of this ms. Table 1 is a great addition, thank you!

**We thank this Reviewer#1 for their re-reviewing of our manuscript and further helpful comments. We have implemented these requests and believe they have helped in ameliorating the revised version of this manuscript.**

The expanded conclusion section (pasted below) will make it easier for readers to identify advances in understanding that result from the study but it is still written so that the only way to make sense of it is to refer to other sections of the paper. I suggest changing statements about "affects" to specific statements about what the effect is….(as highlighted below).

**We agree and thank the Reviewer for pointing this out. We have clarified our findings further in the Conclusion text (see the made changes below).**

**"… our results revealed robust support for predictions of the Neophobia Threshold Hypothesis and Dangerous Niche Hypothesis, in that both dietary breadth and migratory habits were predictors of neophobia.**

**Done - added in “Specifically, feeding generalists were less neophobic than feeding specialists and migratory species were more neophobic than non-migratory species.”**

What do "migratory habits" refer to? Are migratory birds more or less likely to be neophobic?

**Done - please see below.**

**Migratory habits represent expected avian movement through various environments; for the purpose of analysis, we divided these movement categories into ‘migratory’ (whereby a significant part of the species’ populations regularly travels beyond their breeding areas, following a predictable timing and specific routes each season), nomadic (whereby populations are not resident but may constantly move following the availability of sporadic [in time and distribution] resources, and in contrast to migratory species, routes and timing of movements cannot be adequately predicted in the long term), and non-migratory (whereby movement patterns do not comply to definitions outlined above, resident populations). Migratory birds are, according to the Dangerous Niche Hypothesis, more likely to be neophobic than the non-migratory species as they evolved to manoeuvre diverse environments but with limited opportunity to become familiar with local conditions.**

**We refer to the distinction in migratory habits in Table 1, and to predictions regarding neophobia of migratory and non-migratory species in text L296, and we have added further clarification in text L296 (copied below).**

***“Specifically, we tested for differences in neophobia between species varying in migratory habits (i.e. migratory, nomadic and non-migratory species) and habitat exploitation, including feeding and habitat generalists versus specialists. Following the Dangerous Niche Hypothesis, we expected non-migratory species to be less neophobic than migratory ones, as the latter evolved to manoeuvre diverse environments but with limited opportunity to become familiar with local conditions [19,23] (Table 1).”***

**We have also added some clarifications regarding our predictions as well as findings to the Conclusions text (L657-662).**

***“Across four models drawing on both raw data and difference scores as well as data subset for non-domesticated species, our results revealed robust support for predictions of the Neophobia Threshold Hypothesis and Dangerous Niche Hypothesis, in that both dietary breadth and migratory habits were, as expected, predictors of neophobia. Specifically, feeding generalists were less neophobic than feeding specialists and migratory species were more neophobic than non-migratory species.”***

We also found some evidence for additional effects of territoriality, habitat density and diversity, and domestication impacting neophobic responses.

How? In what direction?

**Done - please see below.**

**We agree that this part of the Conclusion was not clear enough in a previous version of our manuscript, and have thus added further clarifications to the text, see L662-667.**

***“*We also found some evidence for additional effects of territoriality, habitat complexity and diversity, and domestication impacting neophobic responses. Namely, less territorial species showed lower object neophobia than more territorial species, species with less complex habitats and those species considered as more habitat specialists were more neophobic than species with more complex habitats and those considered as more habitat generalists, and domesticated species were less neophobic than non-domesticated species.*”***

**Reviewer #2:**

The new version of the manuscript represents a significant improvement over the previous version, both in terms of clarity, theoretical background and scientific rigor. The authors have addressed many of the concerns of the reviewers and editors, and I believe the current version may now be suitable for publication.

While I disagree with certain specific responses provided by the authors, I do not consider these disagreements to be fundamental or detrimental to the overall conclusions of the study. These points mainly reflect differences in interpretation or emphasis rather than flaws in the methodology or reasoning, and they do not undermine the central findings presented in the manuscript.

This study is significant not only for advancing our understanding of neophobia—a key behavioral response to novelty—but also for pioneering an ambitious collaborative research model that investigates cognition through an individual-based, experimentally driven comparative framework.

**Done - please see below**

**We thank the Reviewer#2 for taking the time to re-review our manuscript and for their overall positive evaluation of our manuscript.**

__________________________________________________

In compliance with data protection regulations, you may request that we remove your personal registration details at any time. (Use the following URL: <https://eur03.safelinks.protection.outlook.com/?url=https%3A%2F%2Fwww.editorialmanager.com%2Fpbiology%2Flogin.asp%3Fa%3Dr&data=05%7C02%7Cr.miller7%40exeter.ac.uk%7Cfa78930fde1540abab6808ddd69e3616%7C912a5d77fb984eeeaf321334d8f04a53%7C0%7C0%7C638902697193372654%7CUnknown%7CTWFpbGZsb3d8eyJFbXB0eU1hcGkiOnRydWUsIlYiOiIwLjAuMDAwMCIsIlAiOiJXaW4zMiIsIkFOIjoiTWFpbCIsIldUIjoyfQ%3D%3D%7C0%7C%7C%7C&sdata=frC3aKaxfkQ1HxcWFP1Vdu6ejQZ6hl3SkNmimypDgT0%3D&reserved=0)>. Please contact the publication office if you have any questions.

Ref: Ecological drivers of neophobia across the avian clade

PBIOLOGY-D-24-03430R2

Dear Dr Miller,

Thank you for your patience while we performed technical checks on your submission. Please see below for a list of requirements for you to address while conducting your revisions.

Journal Requirements:

1) Please amend your detailed Financial Disclosure statement. This is published with the article. It must therefore be completed in full sentences and contain the exact wording you wish to be published.

1. State what role the funders took in the study. If the funders had no role in your study, please state: “The funders had no role in study design, data collection and analysis, decision to publish, or preparation of the manuscript.”

2. If any authors received a salary from any of your funders, please state which authors and which funders.

**Done - added all 14 listed funders to the financial disclosure statement and the role of funders is clarified (The funders had no role in study design, data collection and analysis, decision to publish, or preparation of the manuscript).**

**We have checked if any authors received salary from any of the funders and have now included this information in the Financial Disclosure statement. We have also copied the new text in below.**

**“The authors who received a salary from one of the funders are donated with an * and listed below. Raúl O. Gómez and Juan C. Reboreda are Research Fellows of the Consejo Nacional de Investigaciones Científicas y Técnicas (CONICET), from which they receive their salaries. Christine Lattin received 1 month of summer salary from National Science Foundation IOS-2237423. Jimena Lois-Milevicich was supported by a postdoctoral fellowship from CONICET, through which she received a stipend. Rachael Miller received a salary as staff at Anglia Ruskin University. Stephan Reber received a salary from the Swedish Research Council. Christopher Templeton received a salary from the National Science Foundation. Camille Troisi’s salary was paid via ERC Consolidator grant (European Union’s Horizon 2020 research and innovation programme, grant agreement No. 769595). Alizee Vernouillet [LM2] received a salary through a BOF postdoc fellowship (#BOF.PDO.2021.0035.01) from the University of Ghent.”**

2) Please refer to the following article for PLOS Biology's best-practice recommendations for data presentation: <https://eur03.safelinks.protection.outlook.com/?url=https%3A%2F%2Fjournals.plos.org%2Fplosbiology%2Farticle%3Fid%3D10.1371%2Fjournal.pbio.1002128&data=05%7C02%7Cr.miller7%40exeter.ac.uk%7C411f63fc26dc4900a74b08ddd7bb76d3%7C912a5d77fb984eeeaf321334d8f04a53%7C0%7C0%7C638903922332973410%7CUnknown%7CTWFpbGZsb3d8eyJFbXB0eU1hcGkiOnRydWUsIlYiOiIwLjAuMDAwMCIsIlAiOiJXaW4zMiIsIkFOIjoiTWFpbCIsIldUIjoyfQ%3D%3D%7C0%7C%7C%7C&sdata=O%2B9e5b8Ka9tulTMCYgcRWd2TD%2BWhzHSQn3w8T4OSyJY%3D&reserved=0>.

**Done**

Guidelines for statistical reporting, if relevant, may be found here: <https://eur03.safelinks.protection.outlook.com/?url=https%3A%2F%2Fjournals.plos.org%2Fplosbiology%2Fs%2Fbest-practices-in-research-reporting%23loc-statistical-reporting&data=05%7C02%7Cr.miller7%40exeter.ac.uk%7C411f63fc26dc4900a74b08ddd7bb76d3%7C912a5d77fb984eeeaf321334d8f04a53%7C0%7C0%7C638903922332988130%7CUnknown%7CTWFpbGZsb3d8eyJFbXB0eU1hcGkiOnRydWUsIlYiOiIwLjAuMDAwMCIsIlAiOiJXaW4zMiIsIkFOIjoiTWFpbCIsIldUIjoyfQ%3D%3D%7C0%7C%7C%7C&sdata=fxVY2aWYqrxoXZb4vqwWIIAsDRP3T2sEFHJgzJAzJlI%3D&reserved=0>.

If you have any questions or require assistance, please don't hesitate to get in touch via return email. To help us minimize inbox volumes, we kindly ask that you not reply to this email simply to confirm you have resubmitted your revisions.

Kind regards,

Hanan Al-Mandouh

Publications Assistant

PLOS Biology

plosbiology@plos.org

At this stage, we recommend referring to our author guidelines and policies on Data Availability and Copyright to prevent delays at resubmission. Links have been provided below for your ease:

Author guidelines: <https://eur03.safelinks.protection.outlook.com/?url=https%3A%2F%2Fjournals.plos.org%2Fplosbiology%2Fs%2Fsubmission-guidelines&data=05%7C02%7Cr.miller7%40exeter.ac.uk%7C411f63fc26dc4900a74b08ddd7bb76d3%7C912a5d77fb984eeeaf321334d8f04a53%7C0%7C0%7C638903922332997437%7CUnknown%7CTWFpbGZsb3d8eyJFbXB0eU1hcGkiOnRydWUsIlYiOiIwLjAuMDAwMCIsIlAiOiJXaW4zMiIsIkFOIjoiTWFpbCIsIldUIjoyfQ%3D%3D%7C0%7C%7C%7C&sdata=wwLTh%2FsKPt597UVq7FHHJaR2i4Tju9iRLw5Z4Bldjk4%3D&reserved=0>

Revising your manuscript: <https://eur03.safelinks.protection.outlook.com/?url=https%3A%2F%2Fjournals.plos.org%2Fplosbiology%2Fs%2Frevising-your-manuscript&data=05%7C02%7Cr.miller7%40exeter.ac.uk%7C411f63fc26dc4900a74b08ddd7bb76d3%7C912a5d77fb984eeeaf321334d8f04a53%7C0%7C0%7C638903922333262413%7CUnknown%7CTWFpbGZsb3d8eyJFbXB0eU1hcGkiOnRydWUsIlYiOiIwLjAuMDAwMCIsIlAiOiJXaW4zMiIsIkFOIjoiTWFpbCIsIldUIjoyfQ%3D%3D%7C0%7C%7C%7C&sdata=ORTElWYSxV5jK8Uwq2stbY9VVdzUhMBTYz3kzCuQm04%3D&reserved=0>

Data Policy: <https://eur03.safelinks.protection.outlook.com/?url=https%3A%2F%2Fjournals.plos.org%2Fplosbiology%2Fs%2Fdata-availability&data=05%7C02%7Cr.miller7%40exeter.ac.uk%7C411f63fc26dc4900a74b08ddd7bb76d3%7C912a5d77fb984eeeaf321334d8f04a53%7C0%7C0%7C638903922333276195%7CUnknown%7CTWFpbGZsb3d8eyJFbXB0eU1hcGkiOnRydWUsIlYiOiIwLjAuMDAwMCIsIlAiOiJXaW4zMiIsIkFOIjoiTWFpbCIsIldUIjoyfQ%3D%3D%7C0%7C%7C%7C&sdata=d822NqJ%2Fgx%2FFyRcyAj1XP4jV8aHgg%2BhLJe%2B4ffYsnzw%3D&reserved=0>

Copyright: <https://eur03.safelinks.protection.outlook.com/?url=https%3A%2F%2Fjournals.plos.org%2Fplosbiology%2Fs%2Flicenses-and-copyright&data=05%7C02%7Cr.miller7%40exeter.ac.uk%7C411f63fc26dc4900a74b08ddd7bb76d3%7C912a5d77fb984eeeaf321334d8f04a53%7C0%7C0%7C638903922333285835%7CUnknown%7CTWFpbGZsb3d8eyJFbXB0eU1hcGkiOnRydWUsIlYiOiIwLjAuMDAwMCIsIlAiOiJXaW4zMiIsIkFOIjoiTWFpbCIsIldUIjoyfQ%3D%3D%7C0%7C%7C%7C&sdata=%2B5ag6A8oQbUw%2BUBjSYqRZ1Ehond%2F8nsCi5DFPhq1S%2BM%3D&reserved=0>
